# The *Lingula* genome provides insights into brachiopod evolution and the origin of phosphate biomineralization

**DOI:** 10.1038/ncomms9301

**Published:** 2015-09-18

**Authors:** Yi-Jyun Luo, Takeshi Takeuchi, Ryo Koyanagi, Lixy Yamada, Miyuki Kanda, Mariia Khalturina, Manabu Fujie, Shin-ichi Yamasaki, Kazuyoshi Endo, Noriyuki Satoh

**Affiliations:** 1 Marine Genomics Unit, Okinawa Institute of Science and Technology Graduate University, Onna, Okinawa 904-0495, Japan; 2 DNA Sequencing Section, Okinawa Institute of Science and Technology Graduate University, Onna, Okinawa 904-0495, Japan; 3 Sugashima Marine Biological Laboratory, Graduate School of Science, Nagoya University, Toba, Aichi 517-0004, Japan; 4 Department of Earth and Planetary Science, Graduate School of Sciences, University of Tokyo, Bunkyo-ku, Tokyo 113-0033, Japan

## Abstract

The evolutionary origins of lingulid brachiopods and their calcium phosphate shells have been obscure. Here we decode the 425-Mb genome of *Lingula anatina* to gain insights into brachiopod evolution. Comprehensive phylogenomic analyses place *Lingula* close to molluscs, but distant from annelids. The *Lingula* gene number has increased to ∼34,000 by extensive expansion of gene families. Although *Lingula* and vertebrates have superficially similar hard tissue components, our genomic, transcriptomic and proteomic analyses show that *Lingula* lacks genes involved in bone formation, indicating an independent origin of their phosphate biominerals. Several genes involved in *Lingula* shell formation are shared by molluscs. However, *Lingula* has independently undergone domain combinations to produce shell matrix collagens with EGF domains and carries lineage-specific shell matrix proteins. Gene family expansion, domain shuffling and co-option of genes appear to be the genomic background of *Lingula*'s unique biomineralization. This *Lingula* genome provides resources for further studies of lophotrochozoan evolution.

## Abstract (web summary)

Lingulid brachiopods possess calcium phosphate shells. Here, the authors sequence the genome of *Lingula anatine* to show that *Lingula* is evolutionary close to molluscs, but distant from annelids, and identify the genomic background of *Lingula's* unique biomineralization mechanism.

## Graphical abstract

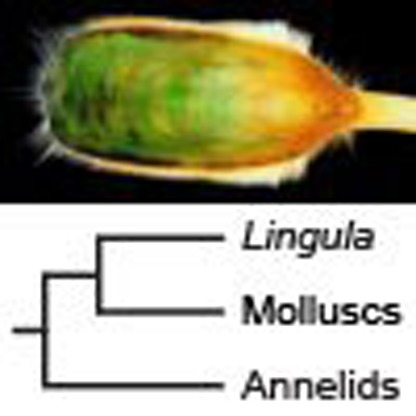

Brachiopods are marine invertebrates with calcium phosphate or carbonate shells. Abundant in the fossil record, Darwin first referred to lingulid brachiopods as ‘living fossils,' because their shell morphology has changed little since the Silurian^1^. Based on molecular phylogeny, brachiopods comprise three subphyla, Linguliformea, Craniiformea and Rhynchonelliformea^2^. The Linguliformea, including the extant genus, *Lingula*, is recognized as the most primitive group, with a fossil record dating back to the early Cambrian and coinciding with the innovation of animal biomineralization^3^. Their shells are composed of calcium phosphate and collagen fibres, characters shared only by evolutionarily distant vertebrates^1^,^4^. Morphologically, brachiopods and bivalves superficially resemble each other. However, lingulid brachiopods exhibit several unique features that distinguish them from molluscs. These include hingeless shells that grow along the dorsal–ventral axis, chitinous chaetae, ciliated lophophores and a tail-like pedicle^1^,^5^. Since the Permian extinction, bivalves have greatly increased their diversity, but the basic body plan of brachiopods has been constrained^6^, which is still a mystery of metazoan evolution.

It has been proposed that *Lingula* might have used calcium phosphate, because the phosphorus concentration in seawater was high in the Cambrian^7^. In fact, some Cambrian arthropods, tommottids and various other problematica also used calcium phosphate for their exoskeletons, whereas other extant invertebrates such as corals, molluscs and echinoderms use calcium carbonate. Studies of mollusc mantle transcriptomes and shell proteomes suggest that gene sets responsible for formation of calcium carbonate-based biominerals such as calcite or aragonite have evolved rapidly. Therefore, mineral homology among molluscs could simply represent parallel evolution^8^. In contrast to mollusc shells and other invertebrate calcified tissues, *Lingula* shells comprises calcium phosphate, laminated, flexible and rich in organic materials^1^. Despite their palaeontological importance, the evolutionary origin of *Lingula* shells is still unclear.

More interestingly, although *Lingula* is a protostome, its embryogenesis exhibits radial cleavage and enterocoelic coelom formation, typical of basal deuterostomes^9^. Despite such unique features, the phylogeny of brachiopods is under debate. Before the 1980s, brachiopods were classified as deuterostomes, based on their mode of development. Then they were grouped within protostomes following an analysis of 18S ribosomal RNAs^10^. This classification was further supported by an analysis of *Hox* genes in brachiopods and priapulids^11^. However, the phylogenetic position of brachiopods is still controversial, in spite of intensive palaeontological^12^ and molecular phylogenetic studies (Supplementary Note 2). For example, whether brachiopods are monophyletic or polyphyletic^2^,^13^ and whether Brachiopoda is close to Phoronida, Nemertea, Mollusca, Annelida or other lophotrochozoan phyla, remains to be resolved^14^,^15^,^16^.

Here we present the first brachiopod genome of the lingulid, *L. anatina.* Our whole-genome phylogenetic analyses support a close relationship between *Lingula* and molluscs. Unexpectedly, we find that contrary to its reputation as a ‘living fossil,' the *Lingula* genome has been actively evolving, with a disorganized Hox cluster and recently expanded gene families. In addition, we show that although *Lingula* shares shell formation-related genes and mechanisms with molluscs, such as chitin synthase (CHS) and bone morphogenetic protein (BMP) signalling, it uses several domain combinations to produce lineage-specific shell matrix collagens, alanine-rich fibres and novel shell matrix proteins (SMPs). We propose that gene family expansion, domain shuffling and co-option of genes appear to comprise the genomic basis of *Lingula*'s unique biomineralization. Together with embryonic and adult tissue transcriptomes, as well as a shell proteome, our comparative genomic analyses provide insights into the evolutionary history of this lophotrochozoan and the origin of phosphate biomineralization.

## Results

### Genome sequencing and assembly

**Figure 1:**
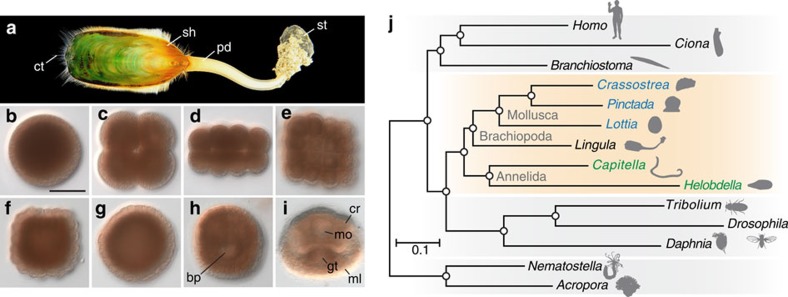
Deuterostomic development of the brachiopod, *L. anatina*, and its close relationship to molluscs. (**a**) Adult (shell length ∼4–5 cm). (**b**–**i**) Embryogenesis: egg (**b**), embryos at 4-cell (**c**), 16-cell (**d**), 32-cell (**e**) and 128-cell stages (**f**), blastula (**g**), late gastrula (**h**) and 2-pair cirri larva (**i**). Scale bar, 50 μm. bp, blastopore; cr, cirri; ct, chaeta; gt, gut; ml, mantle lobe; mo, mouth; pd, pedicle; sh, shell; st, stone. (**j**) Phylogenetic position of *Lingula* among lophotrochozoans (orange box; molluscs are blue; annelids are green). The tree was constructed using the maximum likelihood method with 150 one-to-one orthologues (46,845 amino-acid positions) with LG+Γ4 model. Circles at all nodes indicate 100% bootstrap support.

We sequenced the 425-Mb genome of *L. anatina* (Fig. 1a–i) with ∼226-fold coverage using four next-generation sequencers (that is, Roche 454 GS FLX+, Illumina MiSeq and HiSeq 2500, and PacBio RS II). This effort yielded an assembly with a scaffold N50 size of 294 kb, comparable to those of other lophotrochozoan genomes^17^,^18^,^19^ (Supplementary Note 1, Supplementary Figs 1–3 and Supplementary Tables 1–3). The *Lingula* genome exhibits comparatively high heterozygosity (1.6%) and a low level of repetitive sequences (22.2%) (Supplementary Table 16). Together with a large quantity of transcriptome data from adult tissues and embryonic stages (Supplementary Fig. 4 and Supplementary Table 4), we estimated that *Lingula* contains 34,105 protein-coding gene models, 91% of which are supported by transcriptomes. The mean size of the *Lingula* genes is 6.7 kb with an average of 6.6 introns per gene. These numbers are closer to those of the sea snail, *Lottia gigantea*, than to the leech, *Helobdella robusta*, or the polychaete, *Capitella teleta*^17^. A BLAST top-hits search against the NCBI non-redundant (nr) database shows that 28% of *Lingula* genes are most similar to mollusc genes, but only 12% to annelids, whereas 21% of the genes show no similarity to any known sequence, suggesting that these are unique to the brachiopod lineage (Supplementary Fig. 5). A genome browser, genome and transcriptome assemblies, and related annotation files are available at http://marinegenomics.oist.jp.

### Phylogenetic position of brachiopods

To resolve the phylogenetic position of brachiopods, we carried out phylogenetic analyses (Supplementary Note 2, Supplementary Figs 6–8 and Supplementary Table 5). Analysis based on 150 one-to-one orthologues with 46,845 amino-acid positions from 15 metazoan genomes supports the assertion that *Lingula* is closer to Mollusca than to Annelida (Fig. 1j). Comparative analyses of lineage-specific domain losses among *Lingula*, molluscs and annelids also show that *Lingula* is closely related to molluscs. There are nearly 20 annelid lineage-specific domain losses, which include chordin, haem-binding protein and Death-associated protein domains (Supplementary Fig. 9 and Supplementary Table 6). In addition, microsyntenic analyses showed that *Lingula* and *Lottia* share conservation of a large number of microsyntenic blocks (Supplementary Fig. 10 and Supplementary Tables 7–9), supporting the close phylogenetic relationship between brachiopods and molluscs. Furthermore, intron structures also show similarities between *Lingula* and molluscs, but not annelids (Supplementary Fig. 11 and Supplementary Table 10). Therefore, it may be concluded that Brachiopoda is closer to Mollusca than Annelida, although the phylogenetic relationships of Brachiopoda to Phoronida and Nemertea remain to be resolved.

### The evolving *Lingula* genome

**Figure 2:**
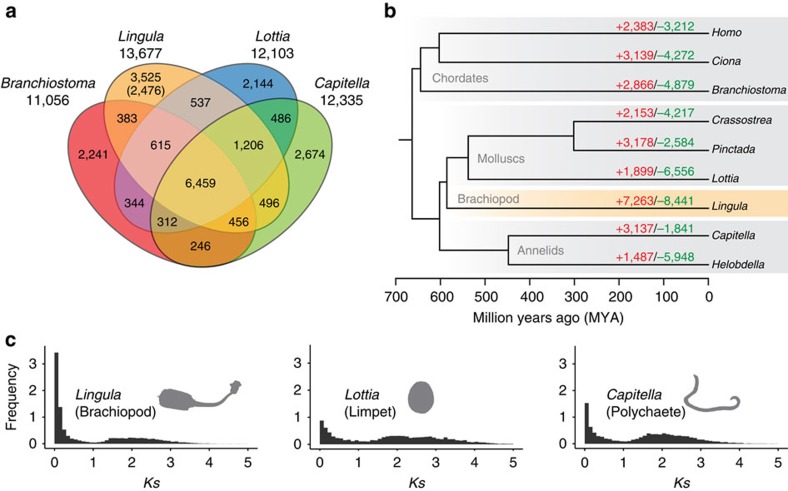
Evolution of the *Lingula* genome is revealed by comparative genomics of lophotrochozoan gene families. (**a**) Venn diagram of shared and unique gene families in four metazoans. Gene families were identified by clustering of orthologous groups using OrthoMCL. The number in parentheses shows unique gene families compared among 22 selected metazoan genomes. (**b**) Gene family history analyses with CAFE. Divergence times were estimated with PhyloBayes using calibration based on published fossil data. Gene families expanded or gained (red), contracted or lost (green). (**c**) Frequency of pair-wise genetic divergence calculated with synonymous substitution rate (*Ks*) among all possible paralogous pairs in the *Lingula*, *Lottia* and *Capitella* genomes.

An abundance of *Lingula* fossils from the Silurian, with morphology very similar to that of extant species, inspired Darwin with the idea of ‘living fossils.' Nevertheless, shells of fossilized and living lingulids show considerable diversity in chemical structure^20^,^21^. Similarly, soft tissue fossils from the Chengjiang fauna reveal morphological changes among lingulid brachiopods^22^. Those findings suggest that lingulid brachiopods have been rapidly evolving. On the other hand, protein-coding genes of the coelacanth, another ‘living fossil,' are reported to be evolving significantly more slowly than those of other tetrapods^23^. Interestingly, we found that *Lingula* genes associated with basic metabolism, such as ribonucleoprotein complex biogenesis and RNA processing, show the slowest evolutionary rate among lophotrochozoans (Fig. 1j). However, we also found a high degree of changes in the genomic structure and gene families (Supplementary Note 3). The *Lingula* genome contains a disorganized Hox cluster. It is divided into two regions, and *Lox2* and *Lox4* are missing (Supplementary Fig. 12). Comparison of gene families shared by amphioxus *Branchiostoma floridae*^24^, *Capitella* and *Lottia* show that *Lingula* has 3,525 unique gene families (Fig. 2a). Further analyses show that the *Lingula* genome contains 7,263 gains and 8,441 losses of gene families. The turnover rate of gene families in *Lingula* is the highest among bilaterians (Fig. 2b).

To better understand evolution of *Lingula* gene families, we further examined the age distribution of duplicated paralogous genes by estimating their non-synonymous substitution rates (*Ks*). Within the youngest duplicated genes (*Ks*<0.1), we found that *Lingula* genes duplicate at a rate approximately two to four times faster than those of other lophotrochozoans (Fig. 2c). A large portion of these young duplicated genes are undergoing negative selection, suggesting a functional constraint on them. We also found that genes related to extracellular matrix are experiencing positive selection (Supplementary Fig. 13), indicating an adaptive need to acquire new functions. These results suggest that the *Lingula* genome has a unique evolutionary history. Decoupling of molecular and morphological evolution has been also reported in the buthid scorpion, *Mesobuthus martensii*^25^. We propose that the morphological constraint upon *Lingula* shells is not due to slow genetic changes. Despite these genomic features, *Lingula* contains genes for transcription factors (Supplementary Table 11) and signalling molecules (Supplementary Table 12) comparable to those of molluscs.

### Expansion of gene families and CHSs

**Figure 3:**
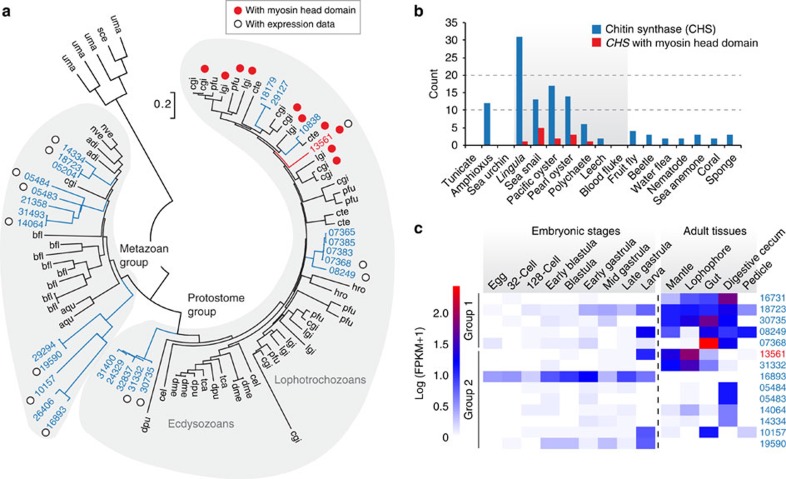
Expansion and expression of *Lingula* chitin synthase genes indicate roles in shell formation and digestion. (**a**) Phylogenetic analysis of chitin synthase (*CHS*) genes using the neighbour-joining method with the JTT model (90 genes, 358 amino acids and 1,000 bootstrap replicates). Three-letter code: adi, coral (*Acropora digitifera*); aqu, sponge (*Amphimedon queenslandica*); bfl, amphioxus (*B. floridae*); cel, nematode (*Caenorhabditis elegans*); cgi, Pacific oyster (*Cr. gigas*); cte, polychaete (*Ca. teleta*); dme, fly (*Drosophila melanogaster*); dpu, water flea (*Daphnia pulex*); hro, leech (*H. robusta*); lgi, sea snail (*L. gigantea*); nve, sea anemone (*Nematostella vectensis*); pfu, pearl oyster (*Pinctada fucata*); sce, baker's yeast (*Saccharomyces cerevisiae*); tca, beetle (*Tribolium castaneum*); uma, corn smut fungus (*Ustilago maydis*). Numbers are *Lingula* gene IDs. (**b**) *CHS* genes detected with BLASTP among 17 selected metazoan genomes. It is noteworthy that *CHS* genes with myosin head domains are only present among lophotrochozoans (grey area). (**c**) The expression of *Lingula CHS* genes in embryonic stages and adult tissues (separated by a vertical dashed line). FPKM, fragments per kilobase of transcript per million mapped reads.

We found lineage-specific expansions of protein domains (Supplementary Table 13) and gene families (Supplementary Table 14). Five of the 20 most expanded families have possible functions in shell formation, including 31 copies of *CHS* genes and 30 copies of carbohydrate sulfotransferase genes (Supplementary Fig. 14). Chitin, a long-chain polymer of *N*-acetylglucosamine, is a characteristic component of arthropod exoskeletons and mollusc shells. Molecular phylogeny shows that nine *Lingula CHS* genes are included in the lophotrochozoan clade (Fig. 3a). In addition, we found that *CHS* genes of lophotrochozoans contain a myosin head domain (MHD) (Fig. 3a,b). It has been proposed that an MHD might have fused to the *CHS* genes during evolution of lophotrochozoans^26^, the only group in which these occur. We found that there is a greater expansion of MHD-containing *CHS* genes in molluscs than in *Lingula* or annelids (Fig. 3a,b). In molluscs, an MHD-containing *CHS* gene is expressed specifically in cells that are in close contact with the larval shell^27^ and that are probably involved in shell formation^28^. Its high expression level during larval shell formation and in adult mantle further suggests a role in mollusc shell formation^19^.

Transcriptome analysis shows that *Lingula CHS* genes are expressed in all adult tissues and in larvae (Fig. 3c). The MHD-containing *CHS* gene is highly expressed in the larval stage and in mantle, suggesting that it may also play a role in *Lingula* shell formation (Fig. 3c). In addition, *CHS* genes are highly expressed in the gut and digestive caecum, indicating that a chitinous peritrophic matrix may also be present in the *Lingula* midgut (Fig. 3c). The expansion of *CHS* genes in the *Lingula* genome and their different expression profiles suggest that chitins participate in brachiopod biomineralization and digestion.

### Comparative genomics of biomineralization-related genes

**Figure 4:**
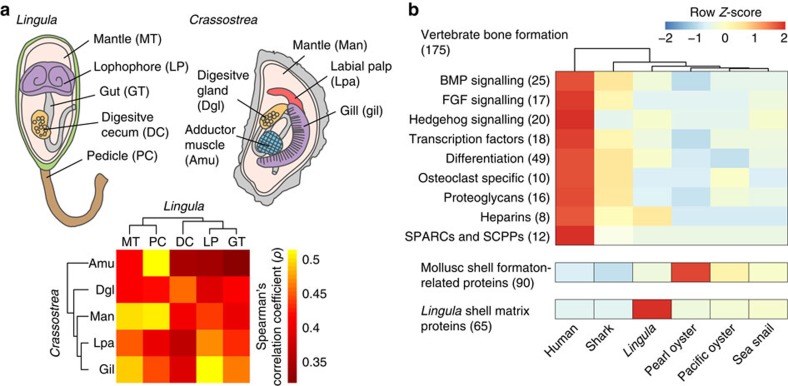
Comparative transcriptomics and genomics reveal different origins of biomineralization-related genes. (**a**) Spearman's correlation coefficient (*ρ*) and hierarchical clustering analyses of transcriptome data from adult tissues of the brachiopod, *Lingula*, and Pacific oyster, *Crassostrea*, in which 6,315 orthologous gene pairs were identified. An adult *Lingula* is shown with the dorsal shell removed and the anus opening to the right. (**b**) Genes involved in formation of vertebrate bone, mollusc shell and *Lingula* shell are compared in biomineralization-capable metazoans. Hierarchical clustering was performed in vertebrate bone formation-associated genes. Numbers of genes analysed are indicated in the parentheses. Shark, *C. milii*; pearl oyster, *Pinctada fucata*; sea snail, *L. gigantea*. BMP, bone morphogenetic protein; FGF, fibroblast growth factor; SCPPs, secreted calcium-binding phosphoproteins; SPARCs, secreted proteins acidic and rich in cysteine.

Animals make hard tissues for protection, support and feeding, mostly in the form of calcified minerals containing carbonate or phosphate^4^,^29^ (Supplementary Note 4 and Supplementary Tables 17–31). Although the shells of *Lingula* and molluscs differ in composition, given that the mantle is the place of shell formation both in brachiopods and molluscs^30^, we first characterized the molecular nature of the *Lingula* mantle. We found that 2,724 genes are specifically expressed in mantle, including those for signal receptors, adhesion molecules and metabolic processes (Supplementary Fig. 15). This suggests that the *Lingula* mantle is responsible for extracellular matrix secretion. Next, we performed comparative transcriptome analyses between *Lingula* and the Pacific oyster, *Crassostrea gigas*^19^ by calculating Spearman's (*ρ*) and Pearson's (*r*) coefficients (Supplementary Fig. 16). Our analyses show that the *Lingula* mantle is related to the *Crassostrea* mantle, indicating a functional similarity between these two organs (Fig. 4a, MT versus Man; Supplementary Fig. 17). We further found that the expression profiles of genes involved in ribosomal machinery are most similar, while those of genes related to chromosome and cell cycle regulation are diverse (Supplementary Fig. 18). Genes related to membrane trafficking are expressed in highly similar ways in *Lingula* and *Crassostrea* mantles, suggesting that the functional similarity comes mainly from genes involved in secretory machinery. However, it is worth noting that the mantle similarity between *Lingula* and *Crassostrea* revealed by our comparative transcriptomics may be the result of sharing common secretory cell types. Whether these two organs share the same evolutionary origin requires more careful examination, although some genes associated with mollusc shell formation, such as calmodulin, calponin and mucin, are also highly expressed in the *Lingula* mantle (Supplementary Table 19).

To gain further insights into the evolution of biomineralization, we conducted comparative genomics and hierarchical cluster analyses, to examine biomineralization-associated genes among vertebrates^31^, molluscs^19^ and *Lingula*. Given that *Lingula* and vertebrates share the use of calcium phosphate, we first examined 175 genes associated with bone formation. We found that the number of *Lingula* homologues to vertebrate bone formation genes is similar to those in other marine invertebrates. There is no specific similarity between *Lingula* and humans (Fig. 4b and Supplementary Table 20). The innovation of the acidic, secretory, calcium-binding phosphoprotein gene family is essential for vertebrate bone formation^31^. However, we failed to find orthologues of secretory, calcium-binding phosphoprotein genes in the *Lingula* genome, although it contains an orthologue of the secreted protein, acidic, cysteine-rich gene (Supplementary Fig. 19). These analyses show that many of the genes involved in bone formation are derived from genome duplication events in the vertebrate lineage^31^. Transcriptome analysis of *Lingula* genes that are associated with bone formation in vertebrates shows that most of these genes are expressed ubiquitously during embryogenesis and in adult tissues, suggesting that they have multiple roles, not limited to biomineralization (Supplementary Tables 21 and 22).

On the other hand, a comparison of 90 genes that are associated with shell formation in molluscs indicates that most of them are shared by bilaterians, whereas mollusc shells contain several lineage-specific proteins (Supplementary Fig. 20). In addition, transcriptome analysis of *Lingula* adult tissues shows that expression of the shared genes is not limited to the mantle. These results suggest that many mollusc shell formation genes have been co-opted independently in mollusc lineages, while they carry out different functions in other bilaterians (Supplementary Table 23). Notably, genes shared between *Lingula* and molluscs, such as calcium-dependent protein kinase and CHS, exhibit high expression in larvae and mantle, indicating that they may also be involved in *Lingula* shell formation (Supplementary Table 24).

### Conserved molecular mechanisms in biomineralization

Given that genes associated with biomineralization have diverse functions and have been co-opted in different species, we next tested whether there is a conserved upstream mechanism for this process. We focused on one of the ancient metazoan signalling pathways, BMPs. Previous studies have demonstrated that BMP signalling plays key roles in biomineralization in both molluscs^32^ and vertebrates^33^. To explore the possible role of BMP signalling during embryogenesis, we first annotated BMP ligands and receptor-regulated Smad. *Lingula* has orthologues for one *Bmp2/4*, one *Bmp5-8* and one *Smad/1/5/9*. Our embryonic transcriptome showed that *Bmp5-8* and *Smad1/5/9* are expressed maternally, whereas *Bmp2/4* is expressed after the early blastula stage (Supplementary Fig. 21).

**Figure 5:**
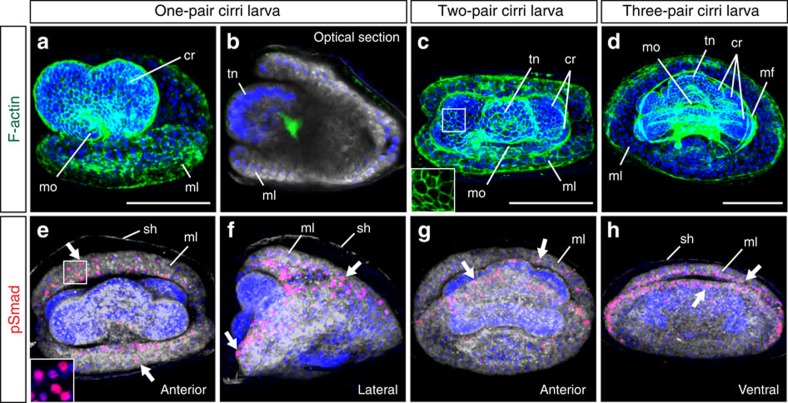
BMP signalling may be involved in larval shell formation. (**a**–**h**) Confocal images of *Lingula* larvae from one-pair cirri to three-pair cirri stages. (**a**–**d**) Filamentous actin (F-actin) staining shows the cellular structure of larvae. Cytoplasmic membranes and nuclei are labelled with CellMask (grey) and DAPI (blue), respectively. Inset in **c** shows the cell boundary at higher magnification. (**e**–**h**) Activation of BMP signalling is monitored by nuclear signals of phosphorylated Smad1/5/9 (pSmad, red). Inset in **e** shows nuclear pSmad signals at higher magnification. It is noteworthy that signals are localized at the margin of mantle lobes (arrows). Orientation of embryos is indicated at the bottom-right corner of each panel. cr, cirrus (cirri); mf, muscle fiber; ml, mantle lobe; mo, mouth; sh, embryonic shell; tn, tentacle. Scale bars, 50 μm.

To visualize activation sites of BMP signals, we employed immunostaining of nuclear phosphorylated Smad1/5/9, an activated mediator. In *Lingula*, embryonic shells are formed on mantle lobes beginning at the one-pair cirri larval stage^12^. Interestingly, we discovered that BMP signalling is activated at the anterior margin of the mantle lobe during *Lingula* larval shell formation (Fig. 5 and Supplementary Fig. 21). This suggests that there may be a conserved mechanism for initiating biomineralization in brachiopods and molluscs. Further functional analyses will provide more rigorous testing of this hypothesis.

### SMPs and fibrillar collagens

Our proteomic analyses of *Lingula* shells identified a total of 65 SMPs (Supplementary Figs 22 and 23). These SMP genes are highly expressed in the mantle (Supplementary Fig. 24). Using comparative genomics, we showed that the composition of *Lingula* SMPs share the highest similarities with those of amphioxus and molluscs (Supplementary Fig. 25). Through an examination of amino acid composition, one of the main characteristics of *Lingula* shells compared with other articulate brachiopods or molluscs is that their SMPs contain a large amount of glycine and alanine^1^. We provided here the first molecular evidence that glycine-rich SMPs are collagens (Supplementary Table 25). In addition, we also found that many novel SMPs are alanine-rich and have low molecular weights (that is, amino-acid length ∼100–200) (Supplementary Fig. 23 and Supplementary Table 26). Pfam analysis of *Lingula* SMPs shows that the most abundant domains are cadherin and collagen, whereas the most abundant proteins contain von Willebrand factor type A and epidermal growth factor (EGF) domains (Supplementary Fig. 23 and Supplementary Table 27). The domain composition suggests that the shell matrix is derived from the extracellular matrix. We further examined the expression profile of these SMPs. We found that 26 SMPs are expressed ubiquitously in all adult tissues, indicating that they have functions other than shell formation (Supplementary Fig. 24). On the other hand, 20 SMPs exhibited specific expression in the mantle. These include collagen, chitinase, glutathione peroxidase, hephaestin, hemicentin and peroxidasin (Supplementary Fig. 24 and Supplementary Table 28). Many of these genes function as extracellular enzymes and ion-binding sites in humans, suggesting that they are probably co-opted in *Lingula* for shell formation. Furthermore, it has been reported that secreted acidic proteins play an important role during the calcification process in mollusc shells^30^ and coral skeletons^34^. We failed to find secreted acidic proteins among *Lingula* SMPs (Supplementary Tables 25 and 26). Instead, we found that there are novel alanine-rich SMPs with a three-helix bundle structure that may confer elastic properties on the *Lingula* shell (Supplementary Fig. 26).

**Figure 6:**
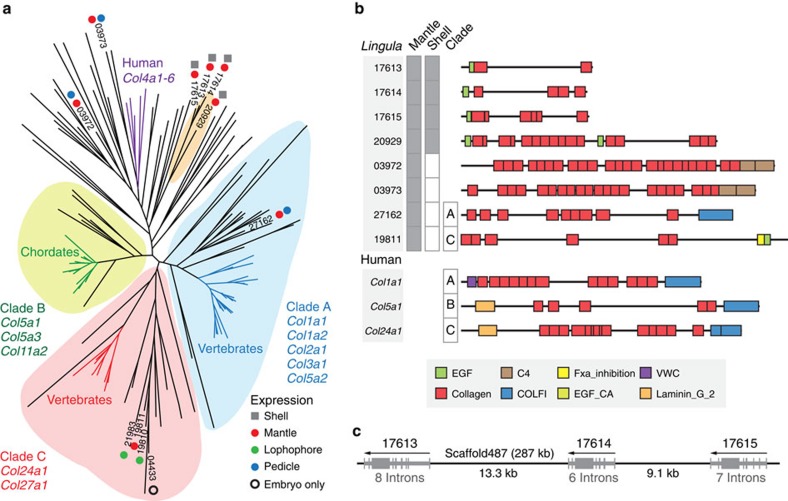
Fibrillar collagens in *Lingula* and vertebrates have different origins. (**a**) Phylogenetic analysis of the collagen triple helix region using the maximum likelihood method with the LG model (159 genes, 542 amino acids, 100 bootstrap replicates). Expression of gene models is supported by the shell proteome (square) and transcriptome (circle). Numbers indicate *Lingula* gene IDs. (**b**) Domain structure of selected collagens. Expression of proteins is shown in grey boxes. C4, type IV collagen C4 domain; COLFI, fibrillar collagens C-terminal domain; EGF, epidermal growth factor-like domain; EGF_CA, calcium-binding EGF domain; Fxa_inhibition, coagulation Factor Xa inhibitory site; VWC, von Willebrand factor type C domain. (**c**) Genomic organization of tandem-duplicated collagen genes expressed in mantle and shell. Arrows indicate the direction of transcription. Grey boxes denote exons.

As the formation of vertebrate bone and *Lingula* shell rely on mineral deposition on fibrillar collagens^1^,^35^, we further examined the evolution of fibrillar collagens. Vertebrate fibrillar collagens are subdivided into three major clades carrying COLFI domains^36^ (Fig. 6a,b). However, our shell proteomic analyses failed to detect fibrillar collagens with COLFI domains in *Lingula* SMPs. Instead, further domain combination analyses show that *Lingula* shell-associated collagens fall into a new group with an EGF domain, which is different from collagens of vertebrate bone (Fig. 6a,b). Intriguingly, some fibrillar collagens probably originated by tandem duplication (Fig. 6c). In addition, we found that *Lingula* contains the highest number of proteins having both EGF and collagen domains among bilaterians (Supplementary Fig. 27 and Supplementary Tables 29 and 30). These findings suggest that EGF-domain shuffling has occurred more frequently in the *Lingula* lineage and may result in new types of collagens with novel domain combinations.

### *Lingula* shell formation and evolution of biomineralization

Mollusc phylogenomic and shell proteomic studies show that mollusc shells may have different origins^8^,^37^,^38^. Although all molluscs use calcium carbonate, different modes of biomineralization have been adapted among brachiopods. Only the Linguliformea makes shells with calcium phosphate^1^. We have shown that *Lingula* used its own gene set for calcium phosphate biominerals, which is different from those used by vertebrates. Given that mineralized vertebrate bone first appeared (∼450 MYA, late Ordovician)^31^ much later than lingulid shells (∼520 MYA, early Cambrian)^22^, it is perhaps not surprising that vertebrate bone and *Lingula* shell have different genetic origins. Although downstream biomineralization-related genes are diverse, we speculate that the metazoan ancestor might use a core set of ancient signalling proteins, such as BMPs and their downstream mediators, to initiate the biomineralization process. We found many calcium-binding and extracellular matrix proteins in *Lingula* shell and mantle. Those proteins have also been reported to participate in bone and shell formation (Supplementary Table 31). This suggests that metazoan biomineralization probably originated from a calcium-regulated extracellular matrix system. Furthermore, we also discovered that Hox4, tyrosinase, CHS, perlucin, chitinase, peroxidasin, mucin and von Willebrand factor type A protein are common shell formation-associated components shared by *Lingula* and molluscs, suggesting that this fundamental gene set was used by their common ancestor (Supplementary Table 31). There are several *Lingula* SMPs encoding enzymes such as glutathione peroxidase, hephaestin and hemicentin, which have no reported function in shell or bone formation. However, interestingly, hephaestin and hemicentin are found in the coral skeletal organic matrix^34^,^39^. This suggests that these extracellular ion-binding proteins in the biomineral matrix may either have been lost in vertebrate bone and mollusc shell, or that they arose independently in *Lingula* and corals.

**Figure 7:**
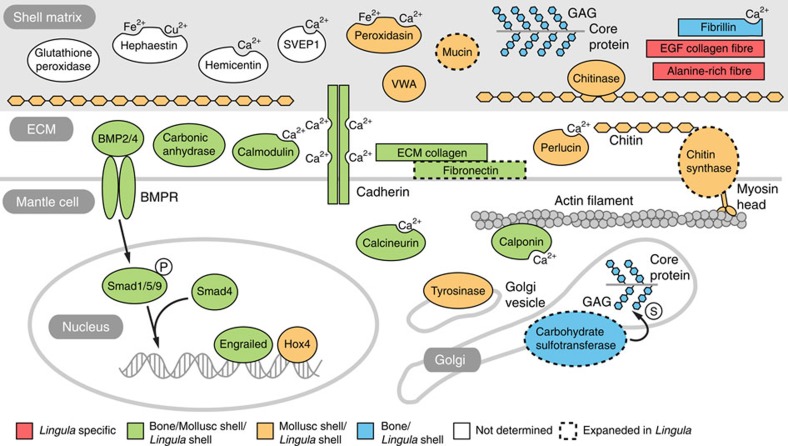
Genes related to biomineralization expressed during *Lingula* shell formation. A schematic illustration of genes involved in *Lingula* biomineralization identified in the present study. Genes are coloured by their known functions in shell or bone formation in molluscs and vertebrates, respectively. Dashed outlines indicate gene families expanded specifically in *Lingula*. BMPR, bone morphogenetic protein receptor; ECM, extracellular matrix; GAG, glucosaminoglycan; SEVP1, Sushi von Willebrand factor type A, EGF and pentraxin domain-containing protein 1; WVA, von Willebrand factor type A domain containing protein. Proteins with ion-binding domains are labelled with Ca^2+^, Fe^2+^ or Cu^2+^. P and S in white circles indicate phosphate and sulfate groups.

In light of the close phylogenetic relationship between *Lingula* and molluscs, we hypothesized evolutionary scenarios for the primitive mode of biomineralization in common ancestors of brachiopods and molluscs. By comparing chemical and molecular features, we present three possible primitive modes (that is, Ca-phosphate primitive, Ca-carbonate primitive and chitin scaffold hypotheses; Supplementary Fig. 28). Given different chemistry and genetic components of their shells, we argue that the calcification process might be a derived feature in brachiopods and molluscs. Instead, chitin localized in epithelial cells may be the primitive character, predating biomineralization. A chitinous scaffold may provide the organic framework for interactions between extracellular matrix and mineral ions^28^. In summary, we propose a possible mechanism for *Lingula* shell formation (Fig. 7). First, the interaction of myosin head-containing CHSs and actin filaments may translate the cytoskeleton organization into an extracellular chitin scaffold. Chitinase in the shell matrix then possibly remodels the chitin scaffold to facilitate the interaction of chitin and chitin-binding proteins. Calcium-binding proteins probably regulate the calcium concentration in the shell matrix and initiate calcium phosphate deposition, together with other structural proteins, such as EGF domain-containing fibrillar collagens and alanine-rich proteins.

## Discussion

We have decoded the first brachiopod genome. We show that the *Lingula* genome has been evolving, instead of remaining static, as one would expect in a genuine ‘living fossil'. Combining transcriptomic and proteomic data, we also show that *Lingula* has a unique system for calcium phosphate-based biomineralization. Perhaps one of the mysteries in animal evolution is the use of calcium phosphate and fibrillar collagens in the formation of biominerals by the evolutionarily distant lingulid brachiopods and vertebrates^1^,^4^. All data presented in this study indicate that *Lingula* and bony vertebrates have adapted different mechanisms for hard tissue formation. Vertebrates probably evolved calcium phosphate-based biomineralization independently, by duplication and neofunctionalization of related genes, while extensive expansion of mineralization-related gene families occurred in the *Lingula* genome.

Indeed, many examples of parallel evolution have been reported. For example, studies on collagen evolution among vertebrates and basal chordates show that three different fibrillar collagen clades occurred independently, a co-option in which collagen was used for biomineral formation of chordates^40^. Similarly, studies of biomineralization genes in sea urchins and molluscs show that there are extensive differences in their expressed gene sets. As molluscs, brachiopods, echinoderms and vertebrates contain different sets of biomineralization genes, biomineral proteins must have arisen independently among metazoans on several occasions^8^,^41^. Taken together, our genomic, transcriptomic and proteomic analyses of *Lingula* biomineralization show similar patterns to those in molluscs^8^ and corals^34^, where co-option, domain shuffling and novel genes are the fundamental mechanisms for metazoan biomineralization. Finally, although we present data, suggesting that Brachiopoda is closer to Mollusca than to Annelida, the phylogenetic position of brachiopods related to other lophotrochozoans remains to be elucidated. The decoded *Lingula* genome provides information essential for such future studies.

## Methods

### Biological materials

Gravid *L. anatina* adults were collected during July and August in Kasari Bay, Amami Island (28.440583 N 129.667608 E; Supplementary Fig. 1). Mature male gonads were dissected for genomic DNA extraction. Maturation of oocytes was induced by injection of 30 μl of 40 mM dibutyryl-cAMP in PBS into the gonad^42^. Artificial spawning was performed by elevating the temperature to 29 °C for 2–6 h, followed by cold shock back to room temperature (∼25 °C)^43^.

### Genome sequencing and assembly

The *Lingula* genome was sequenced using next-generation sequencing technology with a hybrid approach involving four different platforms: Roche 454 GS FLX+, Illumina (MiSeq and HiSeq 2500) and PacBio RS II. Sequencing quality was checked with FastQC (v0.10.1). Raw Illumina reads were quality filtered and trimmed with Trimmomatic (v0.30)^44^. Raw mate pair reads were filtered with DeLoxer^45^ or NextClip (v0.8)^46^ depending on library preparation. Genome assembly was conducted using Newbler (v2.9) with a hybrid assembly approach using data from 454 and Illumina^47^. First, 17 runs of a 1,750-bp library were sequenced using a Roche GS FLX+. This generated 9.6 Gb data with an average read length of 520 bp (Supplementary Figs 1 and 2, and Supplementary Table 2). Second, taking advantage of the enhancement of the read length in Illumina technology, libraries in size ranging from 500 to 620 bp were prepared and sequenced using an Illumina MiSeq. This generated 32.5 Gb of 250 bp long paired-end data (Supplementary Fig. 2 and Supplementary Table 2). To overcome repetitive regions of the genome, mate pair libraries with 1.5–3 kb lengths were prepared using the Cre-Lox recombination approach^45^. In addition, to produce a long mate pair library, the BluePippin system was applied to prepare 5–17 kb DNA fragments and libraries were constructed using Nextera technology^48^. The long mate pair libraries were sequenced to obtain 45.5 Gb of mate pair data using Illumina MiSeq and HiSeq 2500 platforms (Supplementary Table 2).

Finally, Illumina mate pair reads together with 8.5 Gb of PacBio extra-long reads (7–38 kb) were used for scaffolding. Scaffolding was accomplished by mapping paired-end and mate pair reads (1.5–17 kb) from Illumina using SSPACE (v3.0)^49^. PacBio long reads (>7 kb) were mapped to the scaffolds generated by Newbler using BLASR (v20141001)^50^ and upgraded scaffolds were produced with SSPACE-LongRead (v1-1)^51^ (Supplementary Table 2). Gaps in the scaffolds were filled using GapCloser (v1.12-r6) from the SOAPdenovo2 package (r240)^52^ (Supplementary Fig. 2). Redundancy of final scaffolds was removed by calculating BLASTN alignment length and identity using a custom Perl script^47^. Regions of repetitive sequences were identified with RepeatScout (v1.0.5)^53^ and then masked with RepeatMasker (v4.0.3). The genome size was estimated by flow cytometry (Supplementary Fig. 1) as well as by K-mer analysis using SOAPec (v2.01) and Genomic Character Estimator (GCE; v1.0.0) from the SOAPdenovo package^52^. K-mer analysis was also conducted using Jellyfish (v2.0.0)^54^ and a custom Perl script. Completeness of the genome assembly was assessed by searching for the set of 248 core eukaryotic genes using CEGMA (v2.4.010312)^55^.

### Gene model prediction

To obtain high-quality gene models, messenger RNA sequencing (RNA-seq) was performed to obtain transcript information (Supplementary Figs 2 and 4). RNA-seq data (369 million read pairs) from embryos and adult tissues were obtained using an Illumina HiSeq 2500 (Supplementary Fig. 4 and Supplementary Table 4). Transcripts assembled *de novo* with Trinity (r2013_08_14)^56^ were used as expression evidence for predicting gene models (Supplementary Fig. 2). Gene models were predicted with trained AUGUSTUS (v3.0.2) using hints from spliced alignment of transcripts to the masked genome assembly produced with BLAT and PASA (r20130907) (Supplementary Fig. 2).

### Gene family analyses

To analyse gene family evolution in lophotrochozoans, all-to-all BLASTP analysis was performed followed by Markov clustering, to identify orthologous gene groups with OrthoMCL (v2.0.9)^57^, according to the standard protocol using a default inflation number of 1.5. Gene family birth and death was estimated by computing the orthologous gene using CAFE (Computational Analysis of gene Family Evolution; v3.1)^58^. Important transcription factors and signalling components were annotated with Pfam domain searches using HMMER. To identify genes related to specific pathways, which are interesting topics for lineage-specific evolution, the KEGG pathway database was used. Non-synonymous (*Ka*) and synonymous (*Ks*) substitution rates of paired-wise paralogues were calculated with KaKs_Calculator (v2.0)^59^.

### Phylogenetic analyses

To identify robust phylogenetic markers, two strategies were applied. First, OrthoMCL was used to cluster orthologous gene groups from 22 selected metazoan proteomes (Supplementary Table 1, asterisks) and then orthologues with one-to-one orthologous relationships were selected for further analyses using custom Perl scripts. Second, homology searches using a bidirectional best hits (BBH) approach with BLASTP and custom Bash scripts were used to identify the best orthologous pairs among many-to-many orthologous relationships. Alignments of orthologues were performed with MAFFT (v7.130b)^60^. Unaligned regions were trimmed with TrimAl (v1.2rev59)^61^. The maximum likelihood method with LG+Γ4 and GTR+Γ4 models was used to construct phylogenetic trees with RAxML (v8.0.5)^62^. Bayesian trees were constructed with PhyloBayes (v3.3f)^63^ using LG+ Γ4 and GTR+Γ4 models with the first 500 trees as a burn-in. After a run time of ∼20 days (with ∼4,000 generations), convergence of the tree topology was post analysed by sampling every 10 trees.

### Transcriptome analyses

To make the transcriptome more accessible for downstream analysis, transcript assemblies that contained computation errors, expressed at extremely low levels and expressed with highly similar isoforms were eliminated. After RNA-seq assembly, raw reads from each embryonic stage and from adult tissue were mapped back to transcript assemblies using Bowtie (v2.1.0)^64^. Transcript abundance was estimated using RSEM (v1.2.5)^65^. Transcripts expressed at less than one fragments per kilobase of transcript per million mapped reads and isoform representing <5% of a given transcript were filtered. In addition, redundant isoforms were removed with CD-HIT (v4.6)^66^ using 95% identity as a criterion. Next, three sets of criteria were applied to select transcripts with annotated biological functions. First, open-reading frames of transcripts were extracted with the programme getorf in the EMBOSS package (v6.6.0.0). Transcripts with open-reading frames longer than 70 amino acids were retained. Next, the transcriptome was searched against the Pfam database (Pfam-A 27.0) with HMMER (v3.1b1) and against UniProtKB database with BLASTP, respectively. The final representative ‘best' assembly is the union of the three sets of transcripts. To assess the quality of the transcriptome assembly, full-length transcript analysis was applied using a bundled Perl script ‘analyze_blastPlus_topHit_coverage.pl' in the Trinity package^56^. Venn diagram was plotted with jvenn^67^. Gene Ontology enrichment analysis was conducted with DAVID^68^ and PANTHER^69^.

### Comparative transcriptomics

To compare with molluscs, RNA-seq raw reads of selected adult tissues from the Pacific oyster *C. gigas*, which are comparable to those of *Lingula*, were downloaded from OysterDB (http://oysterdb.cn/) and reassembled with Trinity^56^. Orthologues were identified using a BBH approach. To identify transcriptomic similarities between *Lingula* and *Crassostrea* tissues, we calculated Spearman's (*ρ*) and Pearson's (*r*) correlation coefficients using custom Bash and Perl scripts.

### Comparative genomics

Using recent published resources on bone evolution in elephant shark, *Callorhinchus milii*^31^, shell formation in molluscs^19^ and silk genes in two spiders, *Stegodyphus mimosarum* and *Acanthoscurria geniculata*^70^, comparative analyses of biomineralization genes associated with bone, shell and silk formation were conducted. The BBH approach was used to identify orthologous relationships. Genomic scale comparisons of these genes using genomes of humans (*Homo*), sharks (*Callorhinchus*), *Lingula* and molluscs (pearl oyster, *Pinctada*, Pacific oyster, *Crassostrea*, and sea snail, *Lottia*) were made. The heatmap and clustered matrix were created using R (v3.0.2; http:/www.R-project.org/) with the package Bioconductor (v3.0) and pheatmap (v0.7.7).

### Proteomic analyses

SMPs were extracted from adult *Lingula* shells. Dissected shells were ground and cleaned with NaClO. The shell powder was decalcified with acetic acid, reduced and resolved in a 10%–20% gradient gel. Protein bands were excised, in-gel digested with trypsin and analysed with a capillary liquid chromatography system (Dionex, UltiMate 3000) connected to a mass spectrometer (Thermo Scientific, LTQ-XL). Peptide fragments were analysed against *Lingula*-predicted gene models using SEQUEST and MASCOT (v2.3.2) with a false discovery rate of 0.05.

### Immunostaining and F-actin labelling

Embryos were fixed with 4% paraformaldehyde, dehydrated in cold methanol and stored at −20 °C. For antibody staining, embryos were rehydrated in PBST (PBS with 0.1% Tween-20) for 10 min and permeabilized in PBSTX (PBST with 0.1% Triton X-100) for 30 min. Afterwards, embryos were blocked in 3% BSA in PBST for at least 1 h followed by incubation in the primary antibody, rabbit anti-phosphorylated Smad1/5/9 (1:200; Cell Signaling, 9511S) or BODIPY FL phallacidin (1:50; Invitrogen, B607) in 3% BSA in PBST at 4 °C overnight. It is noteworthy that for phallacidin staining, embryos were from batches without methanol treatment. Alexa Fluor goat anti-rabbit secondary antibody (1:400; Invitrogen, A-11037) was used to visualize signals. Nuclei were counterstained with 4,6-diamidino-2-phenylindole (1:1,000; Dojindo, 340–07971) and cytoplasmic membranes were labelled with CellMask Deep Red (1:2,000; Invitrogen, C10046). Embryos were imaged using a Zeiss LMS 780 inverted confocal system.

## Additional information

**How to cite this article:** Luo, Y. -J. *et al*. The *Lingula* genome provides insights into brachiopod evolution and the origin of phosphate biomineralization. *Nat. Commun.* 6:8301 doi: 10.1038/ncomms9301 (2015).

## Supplementary Material

Supplementary Information Supplementary Figures 1-28, Supplementary Tables 1-31, Supplementary Notes 1-4 and Supplementary References
