# Supplementary material for: The Lingula genome provides insights into brachiopod evolution and the origin of phosphate biomineralization
Source: Nat Commun. 2015 Sep 18;6:8301. doi: 10.1038/ncomms9301 (PMC4595640; doi:10.1038/ncomms9301)
Supplement: Supplementary Information — Supplementary Figures 1-28, Supplementary Tables 1-31, Supplementary Notes 1-4 and Supplementary References [file ncomms9301-s1.pdf]

Supplementary Figures

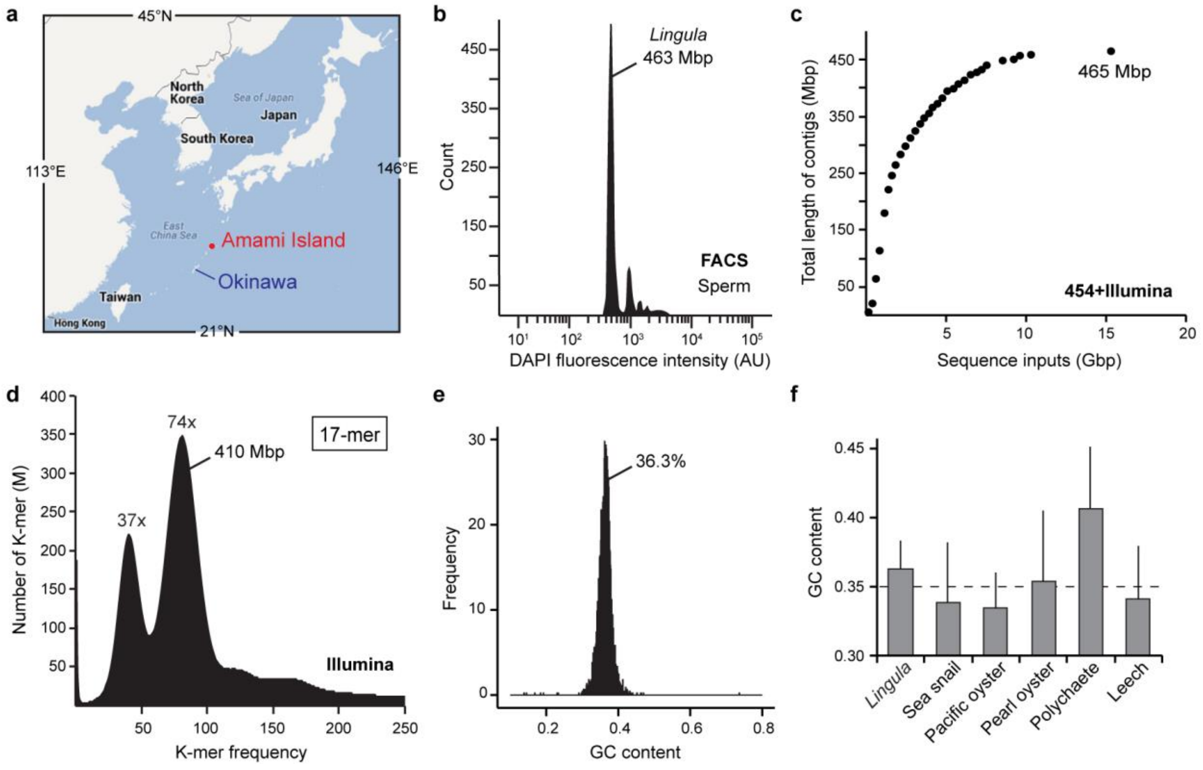

Supplementary Figure 1 | Sampling locality, genome size estimation, and GC content. (**a**) Sampling locality in Amami Island (i.e., Amami Oshima, Japan) and its relative location to Okinawa are shown with coordinates (adapted from Google Maps). (**b**) Sperm cells collected from gravid male gonads were stained with DAPI and subjected to fluorescence-activated cell sorting (FACS) flow cytometry analysis. Sperm with known genome size from zebrafish (*Danio rerio*) were used as an internal standard to estimate the *Lingula* genome size. (**c**) The analysis of stepwise assembly shows that the saturation point is achieved when input sequences reach 10 Gbp from 454 and Illumina reads. (**d**) K-mer analysis (17-mer) using Illumina reads shows two peaks, in which the homozygous peak coverage is twice the heterozygous peak. The estimated heterozygosity rate calculating the ratio of the peaks, is 1.6%. (**e**) Distribution of GC content calculated from 3,830 scaffolds. (**f**) Comparison of GC content in selected lophotrochozoans. Error bars, standard deviation.

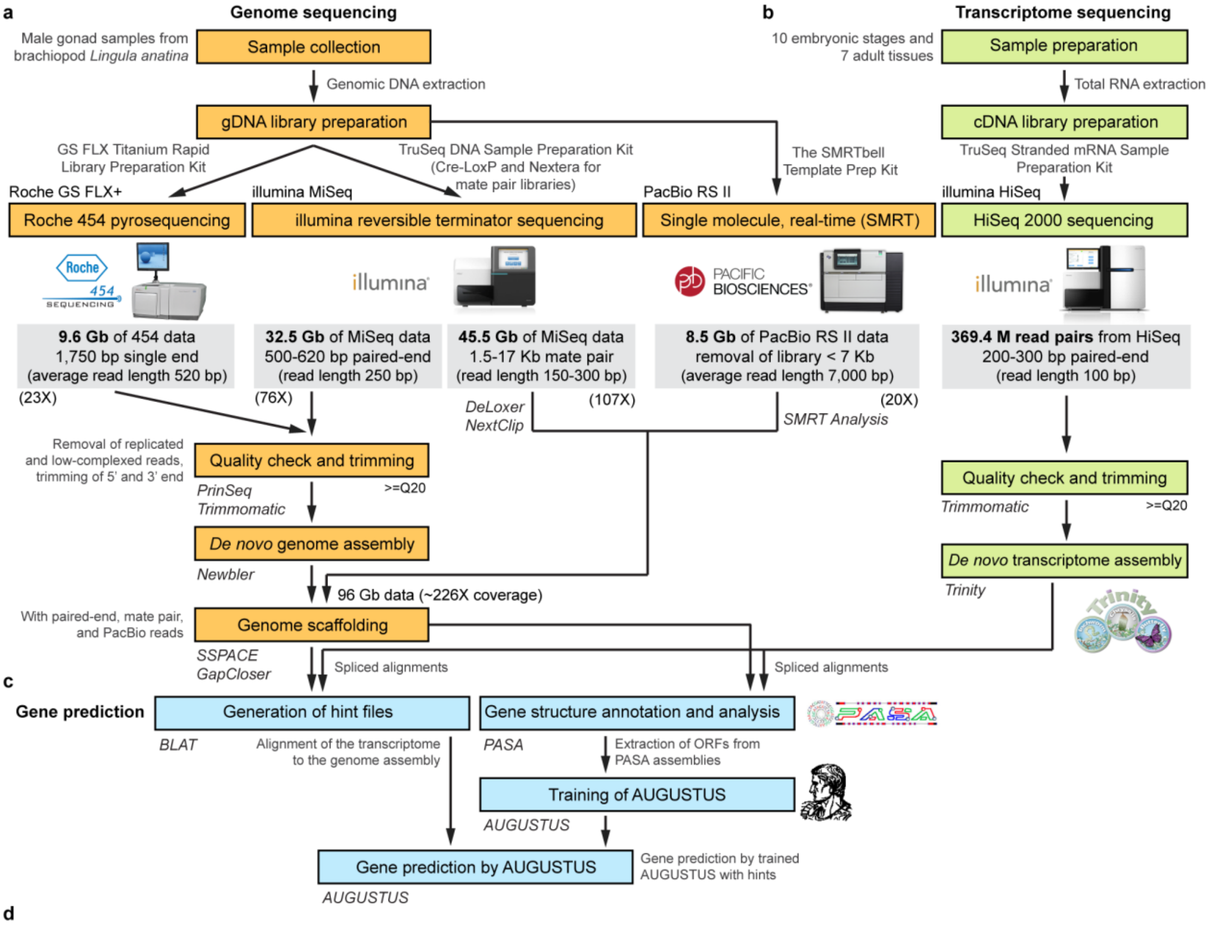

d

Genome size (Mbp)	GC (%)	Repeats (%)	Gaps (Mbp)	Gap (%)	Number of contigs	Number of scaffolds	Contig N50 (kbp)	Scaffold N50 (kbp)	Number of genes	Mean gene size (bp)	Mean transcript size (bp)	Mean intron per gene	Mean intron size (bp)
425	36	22	17	4	14,865	3,830	56	294	34,105	6,669	1,425	6.6	787

Supplementary Figure 2 | Schematic flow of sequencing and assembly of the *Lingula* genome.

(**a**) Genomic DNA from a male gonad was extracted for genome sequencing using Roche 454, Illumina, and PacBio platforms. A total of 96-Gb of data was obtained with approximately 226-fold coverage of the 425-Mb *Lingula* genome. (**b**) Ten embryonic stages from egg to larva and seven adult tissues were collected for RNA-seq and reads were assembled *de novo* using Trinity. (**c**) Transcript information from RNA-seq was used to generate hints by spliced alignment with PASA and BLAT. Gene models were predicted with trained AUGUSTUS. (**d**) Summary of the *Lingula* genome assembly and annotation. Programs used here, such as DeLoxer, NextClip, SMRT Analysis, PrinSeq, Trimmomatic, Newbler, SSPACE, GapCloser, Trinity, BLAT, PASA, and AUGUSTUS are marked in italic.

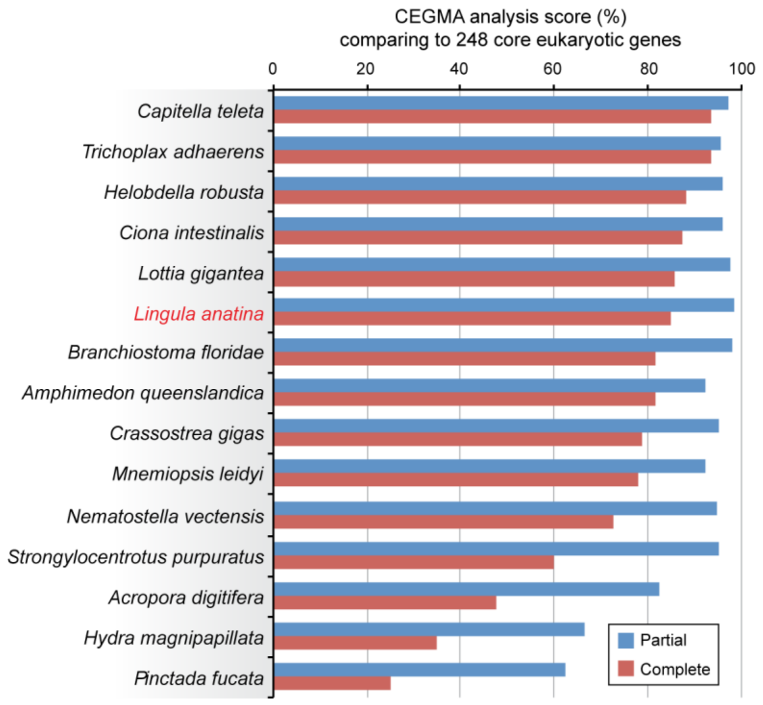

Supplementary Figure 3 | CEGMA completeness analysis.

Completeness of the *Lingula* genome assembly was estimated using Core Eukaryotic Genes Mapping Approach (CEGMA) analysis by searching for 248 core eukaryotic genes against the assembly. The *Lingula* genome (labeled red) was compared with those of selected marine and fresh water invertebrates. They were sorted by degree of completeness (i.e., full length versus partial gene models). This analysis indicates the *Lingula* genome assembly is comparable to those of well-assembled invertebrate genomes.

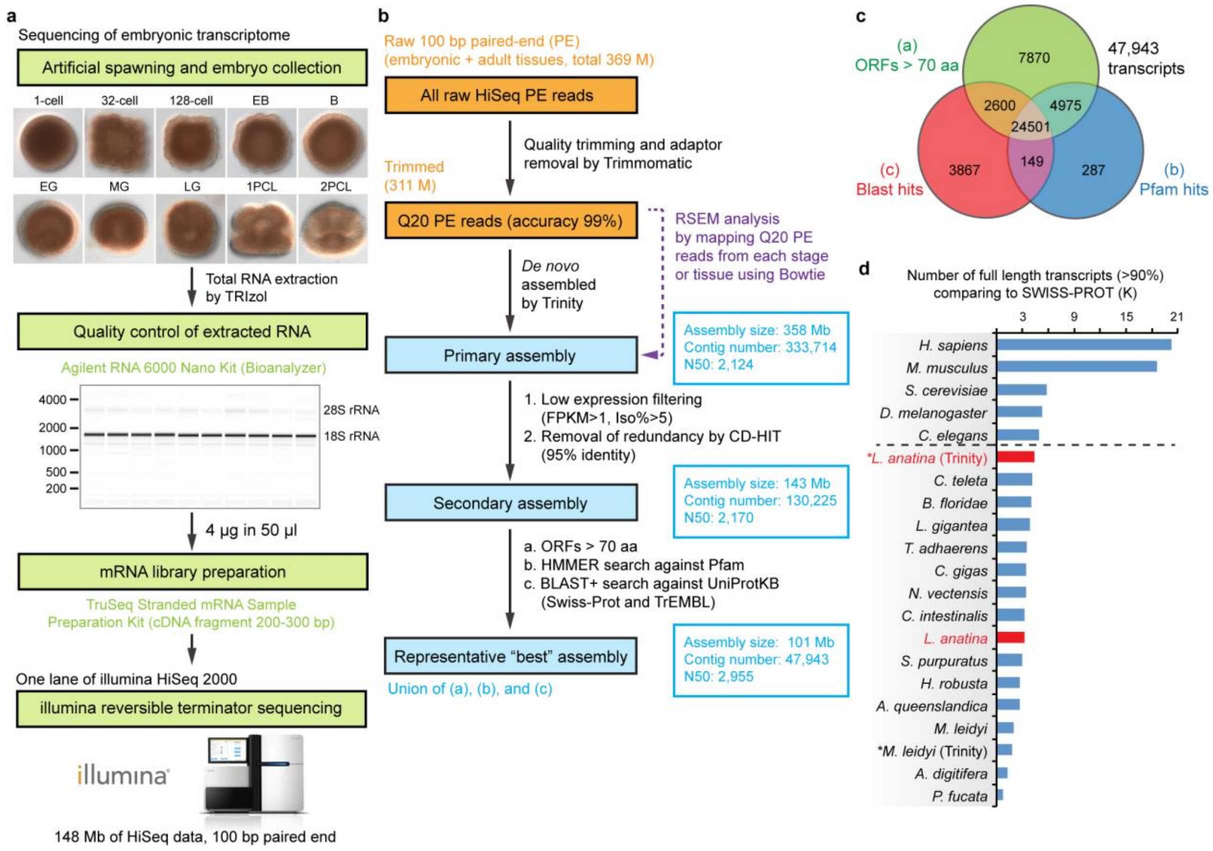

Supplementary Figure 4 | Transcriptome sequencing, assembling, and analyses.
(**a**) Flow chart of transcriptome sequencing with embryonic samples as an example. Extracted RNA is quality checked with a Bioanalyzer to be sure there is no RNA degradation. Note that expression level of 28S rRNA is extremely low in *Lingula*. After mRNA library preparation, samples were subjected to HiSeq sequencing. (**b**) Procedures for assembling the transcriptome. Summary of assembly statistics is given in blue boxes. FPKM, fragments per kilobase of transcript per million mapped reads. ORFs, open reading frames. (**c**) Venn diagram for the final transcriptome assembly containing 47,943 transcripts obtained from three sets of filtering criteria. (**d**) Transcript completeness analysis. Selected gene models predicted from genomes and transcripts assembled with Trinity (marked by asterisks) were mapped to the Swiss-Prot database to estimate the completeness of the given transcripts by checking their sequence alignment rate. Dashed line separates the well-annotated organisms from the others. The *Lingula* gene models and transcriptome are labeled in red.

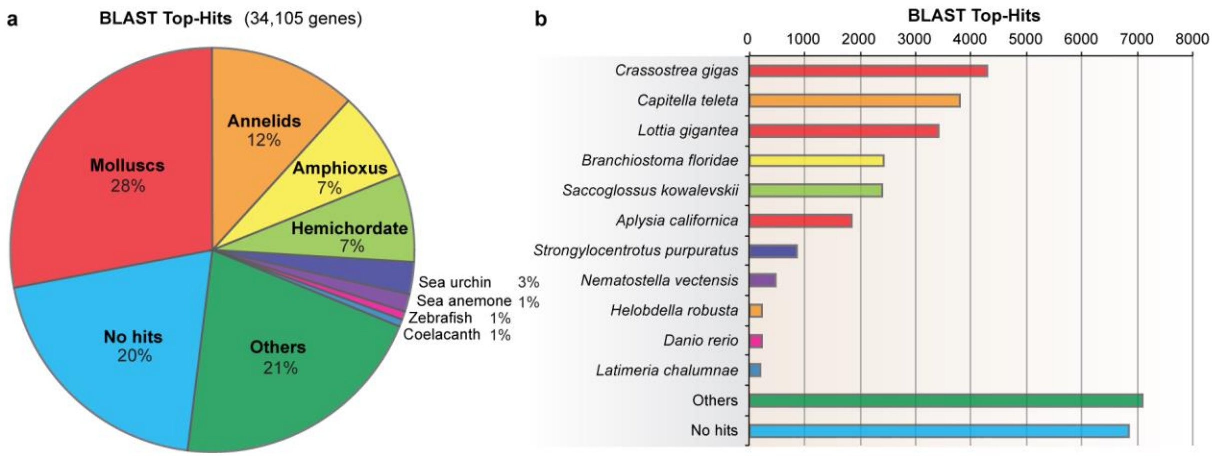

Supplementary Figure 5 | BLAST top-hits analysis against the NCBI nr database.
(**a**) Pie chart of top-hits results among 34,105 gene models in the current *Lingula* genome assembly. *Lingula* has the highest gene similarity to molluscs (28%). A large number of gene models (20%) cannot be assigned to any known sequences. (**b**) More detailed categories for species where the top-hits are distributed. The color code is the same as that of the pie chart. The top-hit species is the Pacific oyster, *Crassostrea gigas* (~4,300 genes). Note that many top-hits are to amphioxus and hemichordate (~5,000 genes). BLAST search was conducted with an e-value cutoff of 1e^{-5}.

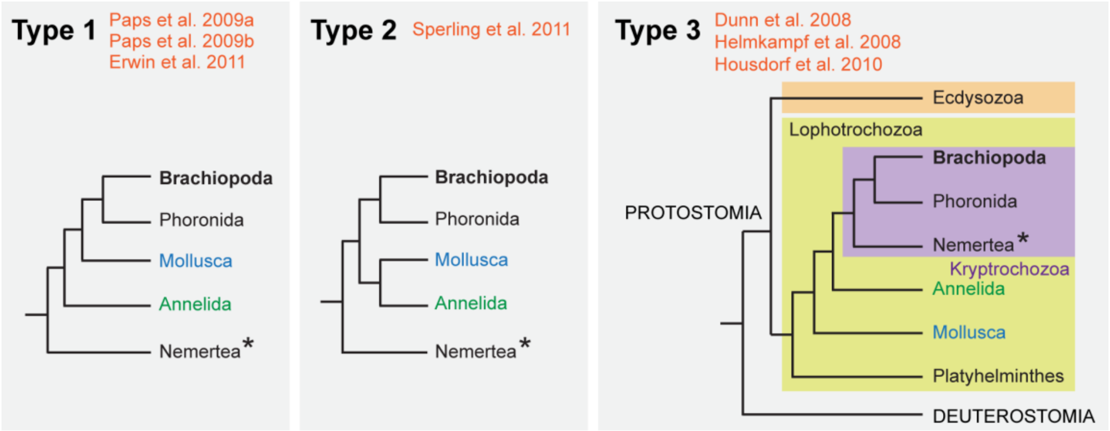

Supplementary Figure 6 | Current hypotheses on the phylogenetic relationship of brachiopods among lophotrochozoans.

Topology type 3 suggests that Brachiozoa (i.e., Brachipoda+Phoronida) is a sister group to Nemertea. In addition, a close relationship between Brachiopod and Nemertea is supported by Bourlat et al. (2008)[1] and Hejnol et al. (2009)[2], whereas a close relationship between Brachiopod and Mollusca is supported by Struck et al. (2014)[3]. The group Kryptrochozoa contains Brachiopoda, Phoronida, and Nemertea (purple box). In contrast, topologies type 1 and 2 show that Brachiozoa is more distant from Nemertea, and closer to Mollusca and a group of Mollusca and Annelida, respectively. Asterisks indicate the position of Nemertea. See Supplementary Table 5 for further information.

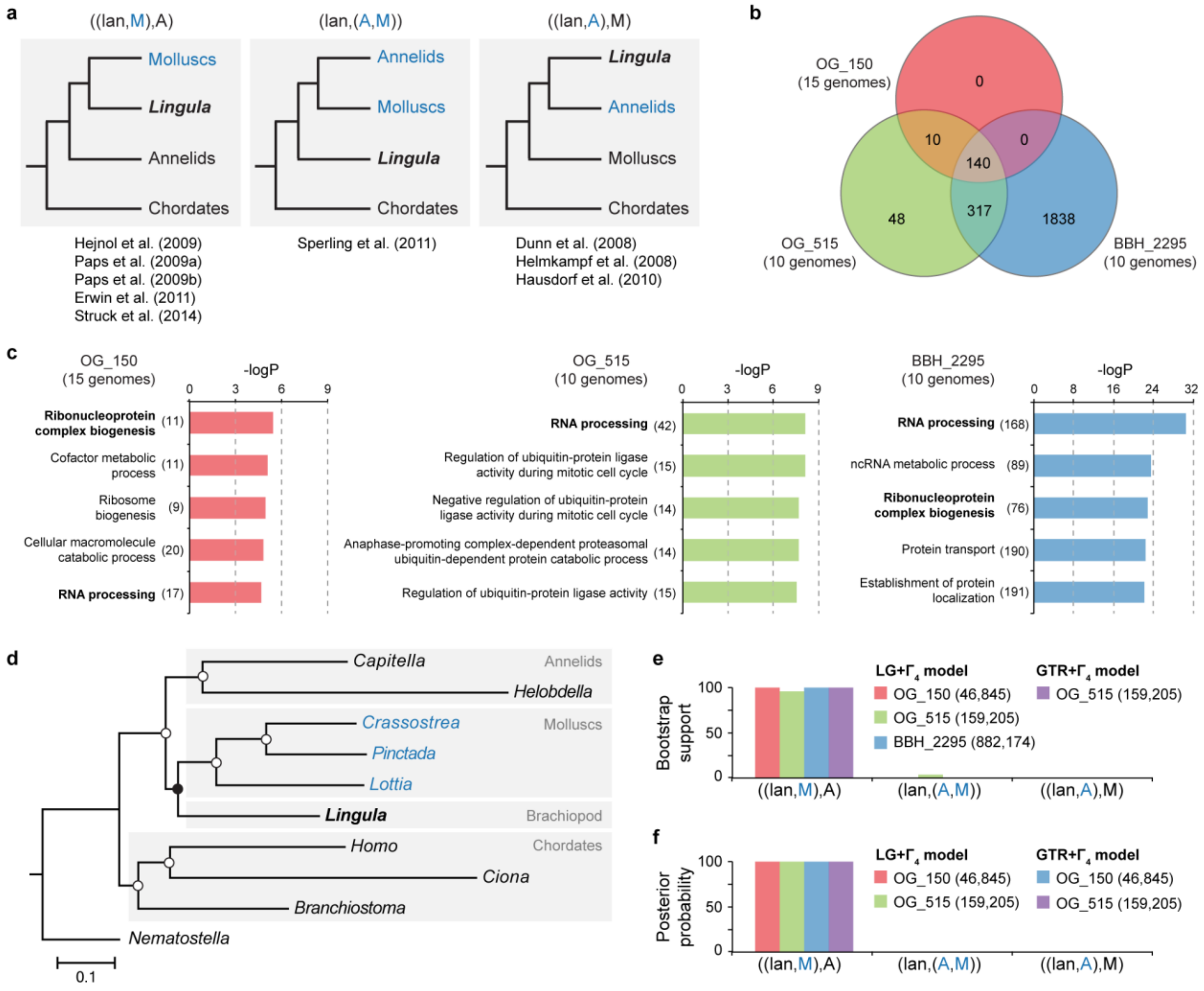

Supplementary Figure 7 | Phylogenetic analyses with three sets of phylogenetic markers.

(**a**) Simplified version of current hypotheses on the possible phylogenetic position of *Lingula* (abbreviated as lan in Newick format) to molluscs (M) and annelids (A). See Supplementary Table S5 for references. (**b**) Venn diagram of three sets of phylogenetic markers detected from the *Lingula* genome. OG, orthologs identified by orthologous grouping; BBH, orthologs identified by bidirectional best hits. (**c**) Gene ontology enrichment analysis of biological process for three sets of phylogenetic markers. Numbers of genes are indicated in parentheses. (**d**) Phylogenetic tree generated from 10 genomes using three sets of phylogenetic markers. Open circles at the nodes indicate 100% bootstrap support from all three sets analyzed using the maximum likelihood (ML) method. Values for the topology of the *Lingula*/Mollusca grouping obtained (solid circle) are shown with bootstrap support from (**e**) an ML analysis and posterior probability from (**f**) a Bayesian analysis. Parentheses show the amino acid positions used for the test.

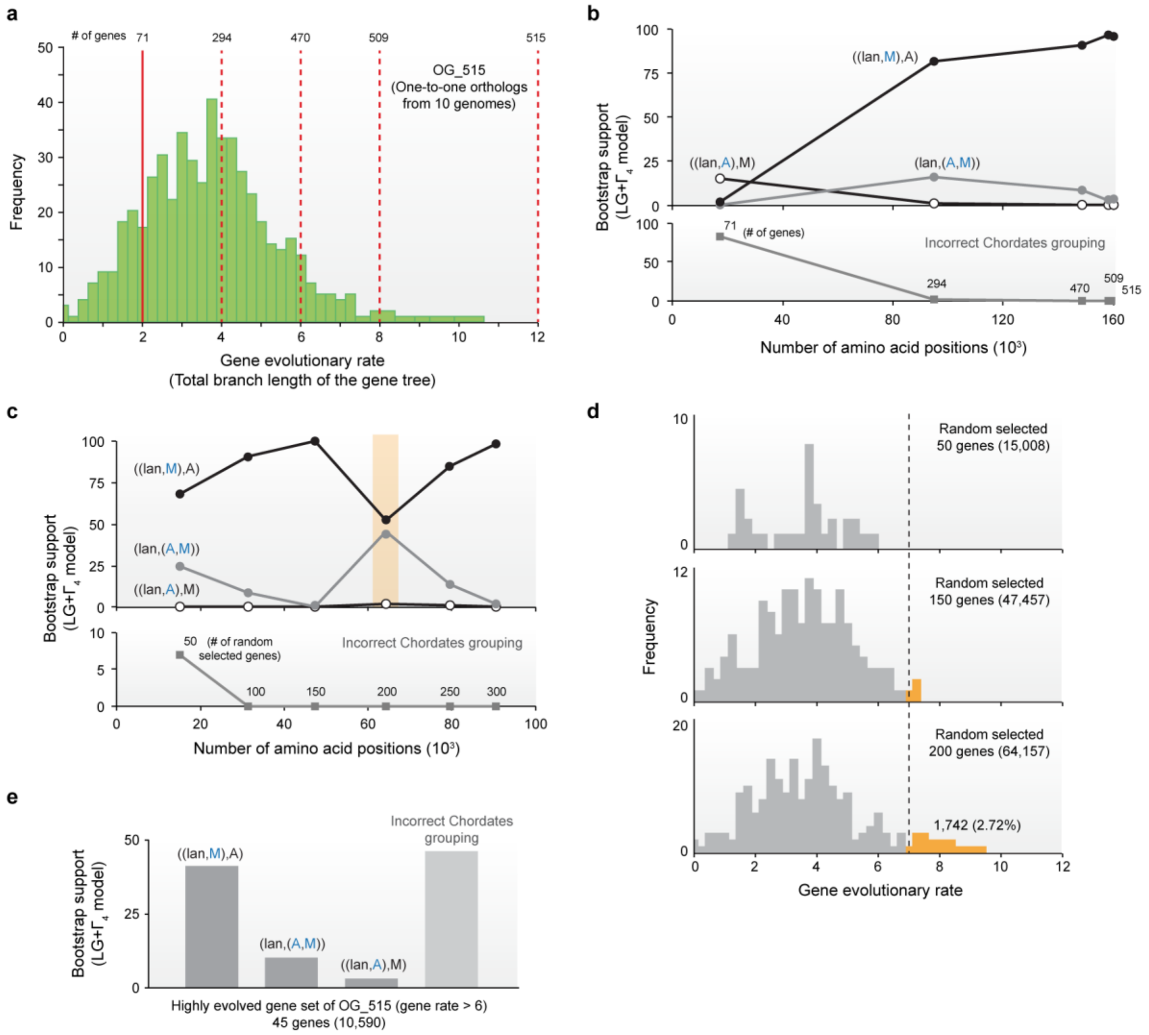

Supplementary Figure 8 | Effects on sampling different phylogenetic markers.

(**a**) Distribution of gene evolutionary rates of 515 one-to-one orthologs from 10 selected metazoan genomes, which was categorized into 5 sets by red lines (where solid line denotes the slowest evolving genes and dashed lines for the others). (**b**) Bootstrap analysis of sequential selected marker sets with evolutionary rate from slowest to fastest. Abbreviation for Newick format: lan, *Lingula*; M, molluscs; and A, annelids. ((lan,M),A), back line with solid circles; ((lan,A),M), black line with open circles; (lan,(A,M)), grey line. Incorrect grouping within the chordates is shown with grey lines with squares in the bottom of the panel. (**c**) Bootstrap analysis of random selected marker sets with incremental sampling size of 50 genes. Orange box indicates the gene set with relatively high evolution rates compared to the others. (**d**) Distribution of evolutionary rates of gene sets shown in (c). Highly evolving genes are labeled in orange. (**e**) Bootstrap analysis of the highly evolving gene set with a gene rate larger than six.

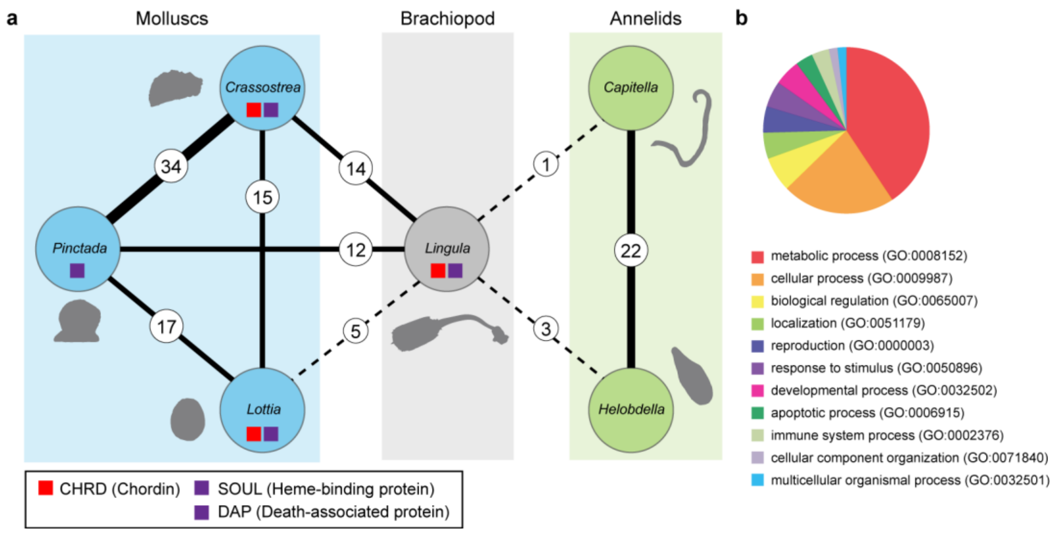

Supplementary Figure 9 | Pairwise comparison of lineage-specific domain loss. Pairwise comparison of lineage-specific domain loss among *Lingula*, molluscs, and annelids. (**a**) Aanalysis of pairwise lineage-specific domain loss. Numbers of pairwise lineage-specific domain losses are indicated in the circles. Thickened solid lines connecting given pairs are proportional to the value of the loss numbers. Dashed lines indicate low lineage-specific-domain losses between the pairs. CHRD (CHRD domain, PF07452) domain is lost in the pearl oyster (*Pinctada*) and annelids. SOUL (heme-binding protein, PF04832) and DAP (Death-associated protein, PF15228) domains are lost in annelids. (**b**) Functional classification of human genes containing 22 domains lost in annelids, based on GO biological process.

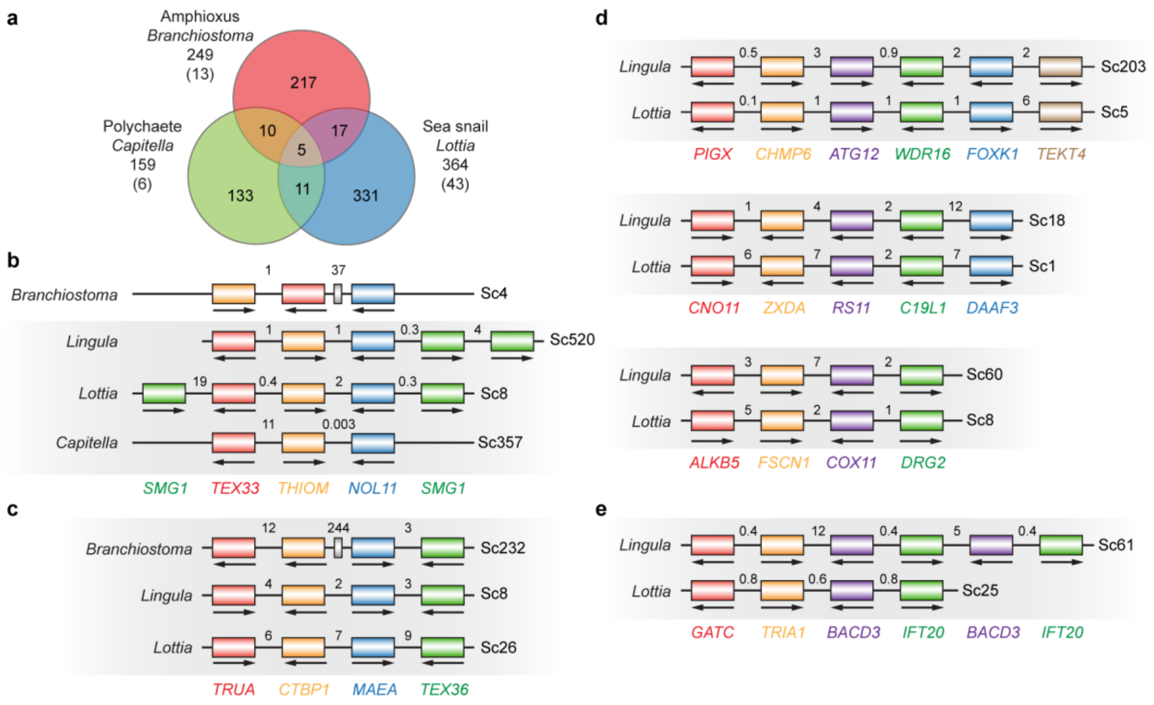

Supplementary Figure 10 | Microsynteny analyses of tightly linked microsyntenic blocks in *Lingula* compared to those of amphioxus, sea snails, and polychaetes.

(**a**) Venn diagram of the numbers of microsyntenic blocks (genes>2) shared by *Lingula* and *Branchiostoma* (amphioxus), *Lottia* (sea snail), and *Capitella* (polychaete), respectively. Numbers of longer blocks (genes>4) are shown in parentheses. (**b**) An example of very short (genes=3) neighboring tightly-linked blocks (NTBs; <20 kbp) shared by all four bilaterian genomes, where inversions and insertions are found in *Branchiostoma* and gene *SMG1* is not found in *Capitella*. Genes with the same color code are members within the same ortholog group. Gene names are given by human UniProt entry name. Grey box denotes that there are other genes in that region. Numbers indicate genomic distance (kbp). Sc, scaffold. (**c**) An example of short (genes=4) NTBs shared by *Lingula*, *Branchiostoma*, and *Lottia*, but not in *Capitella*. A gene insertion is found in only in *Branchiostoma*. (**d**) Examples of long (genes=4-6) NTBs only shared by *Lingula* and *Lottia*. (**e**) An example of long (genes=4) NTBs only shared by *Lingula* and *Lottia*, where two genes (*BACD3* and *IFT20*) tandem duplicated in *Lingula*. See Supplementary Tables 7-9 for full lists.

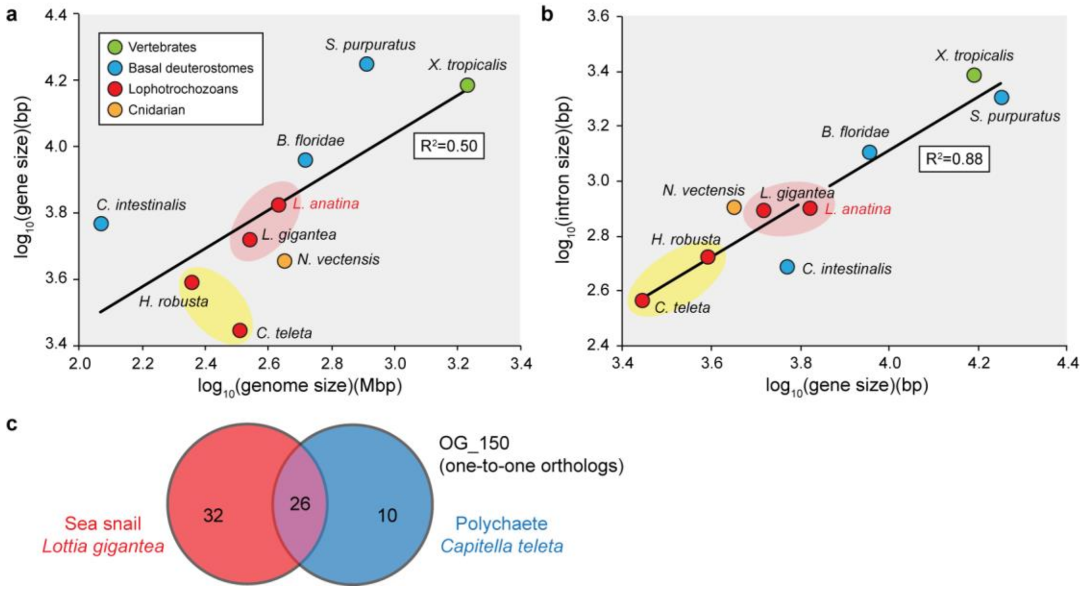

Supplementary Figure 11 | Comparison of intron structure in selected metazoan genomes.

(**a**) Regression analysis of gene size and genome size. R^2, correlation coefficient. (**b**) Regression analysis of intron size and gene size. Close relationships between *Lingula* and sea snails (*Lottia gigantea*) and annelids are circled in red and yellow, respectively. (**c**) Analysis of conserved intron numbers using 150 one-to-one core metazoan gene sets between *Lingula*, *Lottia* and *Capitella*.

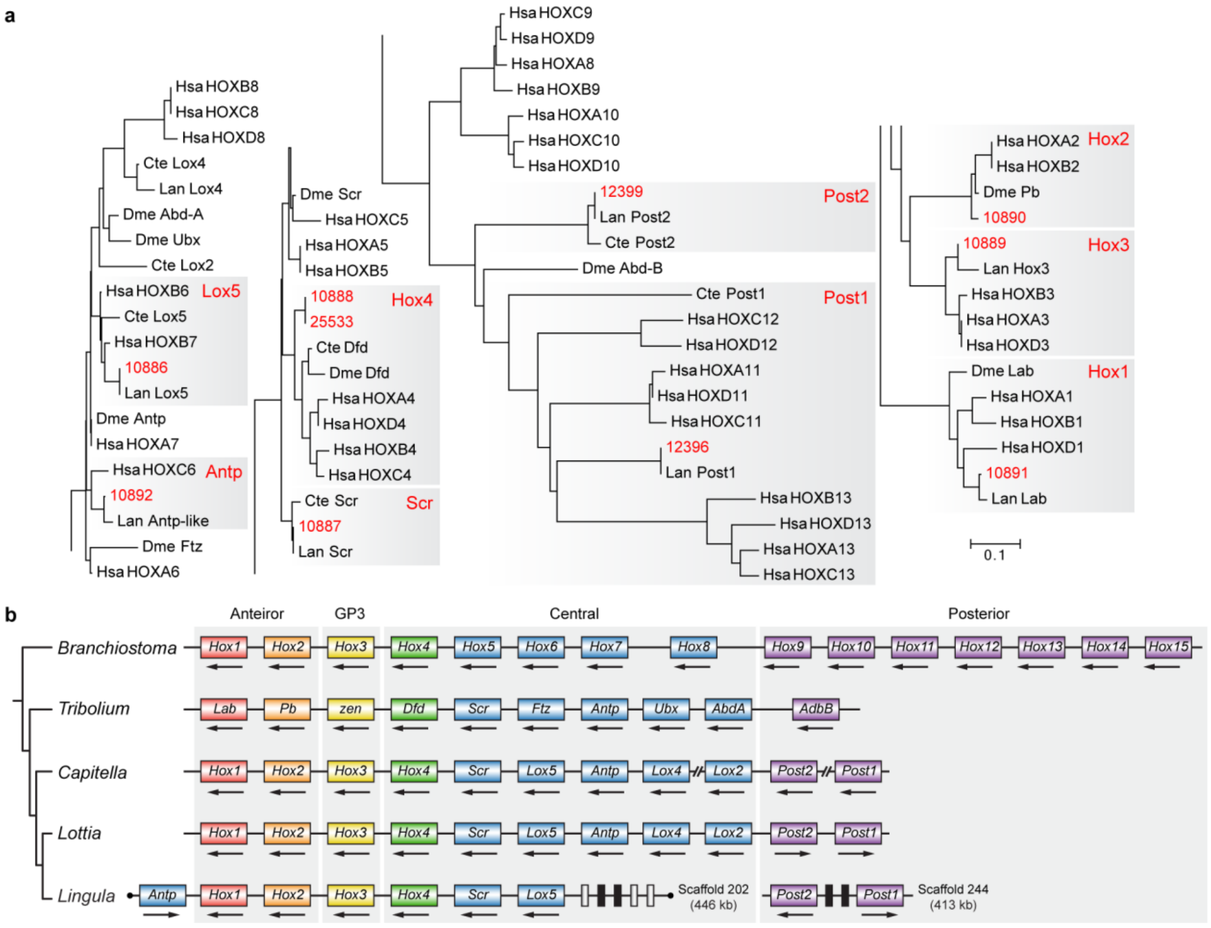

Supplementary Figure 12 | Hox genes in the *Lingula* genome.

(**a**) Phylogeny of Hox genes. The tree was built with the homeobox domain (54 positions) of 82 Hox genes from humans (Hsa), *Drosophlia* (Dme), *Capitella* (Cte), and *Lingula* (Lan) using the neighbor-joining method with the JTT model and 1,000 bootstrap replications. No *Lox2* and *Lox4* homologs can be found among the *Lingula* gene models. *Lingula* gene models are labeled in red. (**b**) The Hox cluster in the *Lingula* genome is disorganized, with *Antp* connected to *Hox1*. Black dots indicate the end of the scaffold. Double slashes signify non-continuous linkage between two genes. Arrows denote the direction of the transcript. Grey boxes represent non-Hox gene models with homology in UniProt. Black boxes represent non-Hox gene models without detectable homology in UniProt. *Branchiostoma* (amphioxus), *Tribolium* (beetle), *Capitella* (polychaete), and *Lottia* (sea snail).

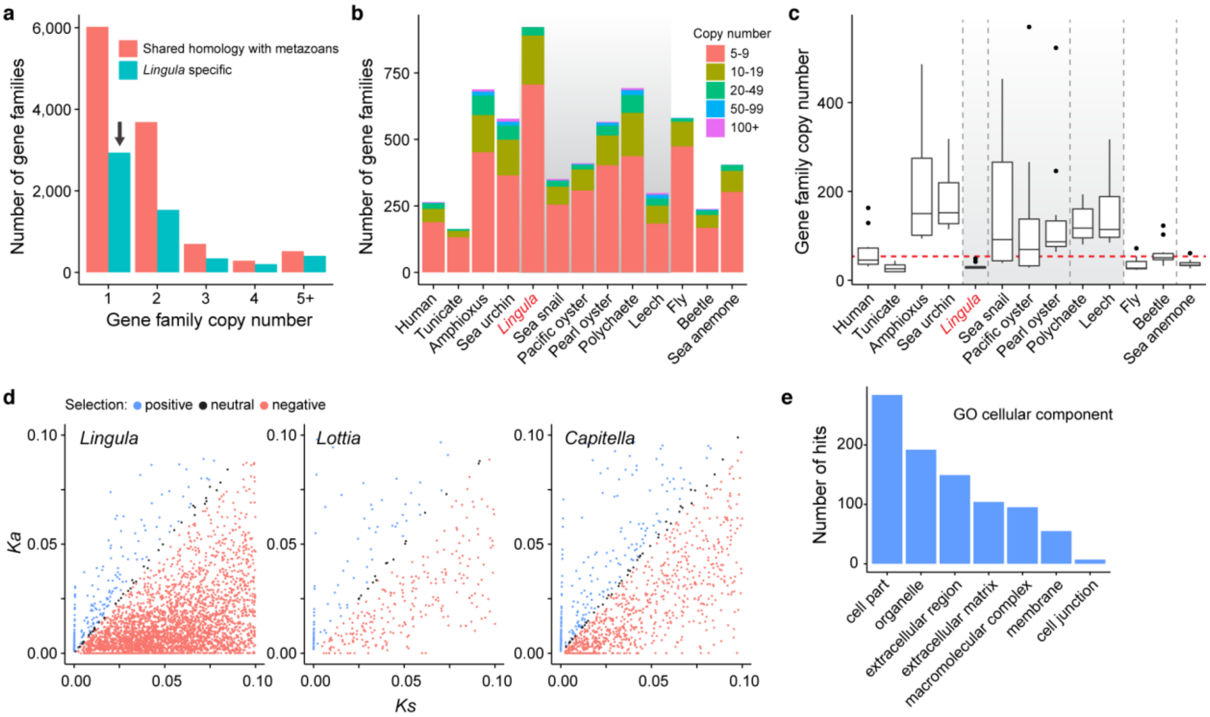

Supplementary Figure 13 | Evolution of *Lingula* gene families.

(**a**) Distribution of shared and *Lingula*-specific gene family sizes. *Lingula*-specific genes with no homology and comprising only one copy are considered orphan genes (arrow). (**b**) Distribution of gene family sizes among metazoans. (**c**) Boxplot of the 10 most expanded gene families among metazoans. Grey area denotes lophotrochozoans. Vertical grey dashed lines mark each animal group, and the horizontal red dashed line shows the upper limit of copy number among *Lingula* gene families. (**d**) Scatter plots of non-synonymous (*Ka*) and synonymous substitution rates (*Ks*) on a fine scale (≤ 0.1) among three lophotrochozoans. $Ka/Ks > 1.05$, positive selection (blue); Ka/Ks = 0.95-1.05, neutral selection (black); $Ka/Ks < 0.95$, negative selection (red). (**e**) Young positively selected genes (with $Ks \leq 0.1$) annotated by GO cellular component.

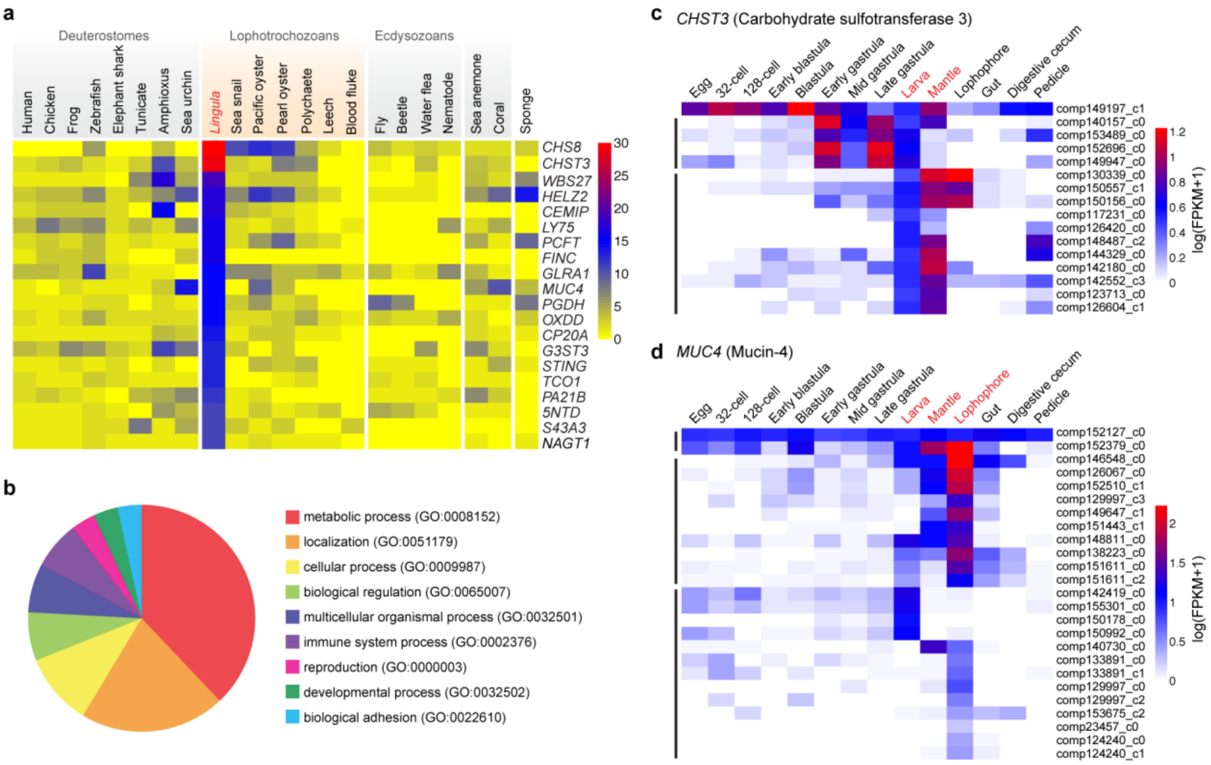

Supplementary Figure 14 | Expansion of *Lingula* gene families.

(**a**) The 20 most expanded gene families in *Lingula* with detectable homology and functional annotation compared to 21 selected metazoan genomes. Gene names are given from the best hits to the human proteome (HUMAN) from UniProt, except of that *CHS8*, which is from the corn smut fungus, *Ustilago maydis* (USTMA). (**b**) Functional classification of expanded gene families (>10) based on GO biological process. (**c**) Heat map of an expanded gene family, carbohydrate sulfotransferase 3 (*CHST3*), highly expressed in larvae and mantle tissue. Embryonic stages or adult tissues with high expression levels are labeled in red. Transcript IDs are shown on the right side. Vertical lines indicate clustering groups. FPKM, fragments per kilobase of transcript per million mapped reads. (**d**) Heat map of expanded gene family, mucin-4 (*MUC4*), highly expressed in larvae, mantle, and lophophore.

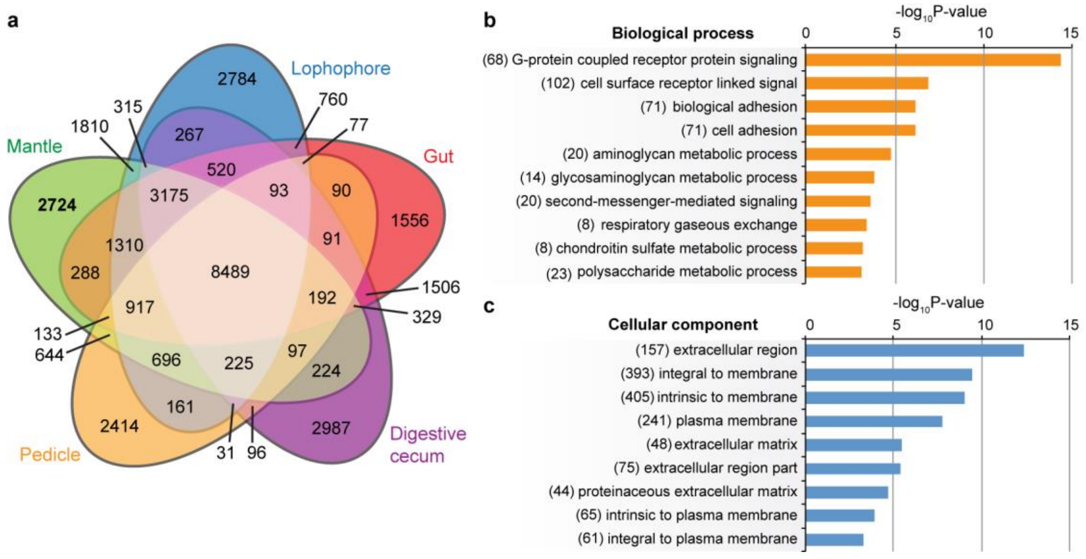

Supplementary Figure 15 | Gene sets specifically expressed in *Lingula* mantle tissue.

(**a**) Venn diagram of gene expression patterns among five different adult tissues, including the mantle, lophophore, gut, digestive cecum, and pedicle. (**b**) Top 10 biological process categorized by GO enrichment analysis. (**c**) Top nine cellular components categorized by GO enrichment analysis. Numbers of genes are indicated in parentheses.

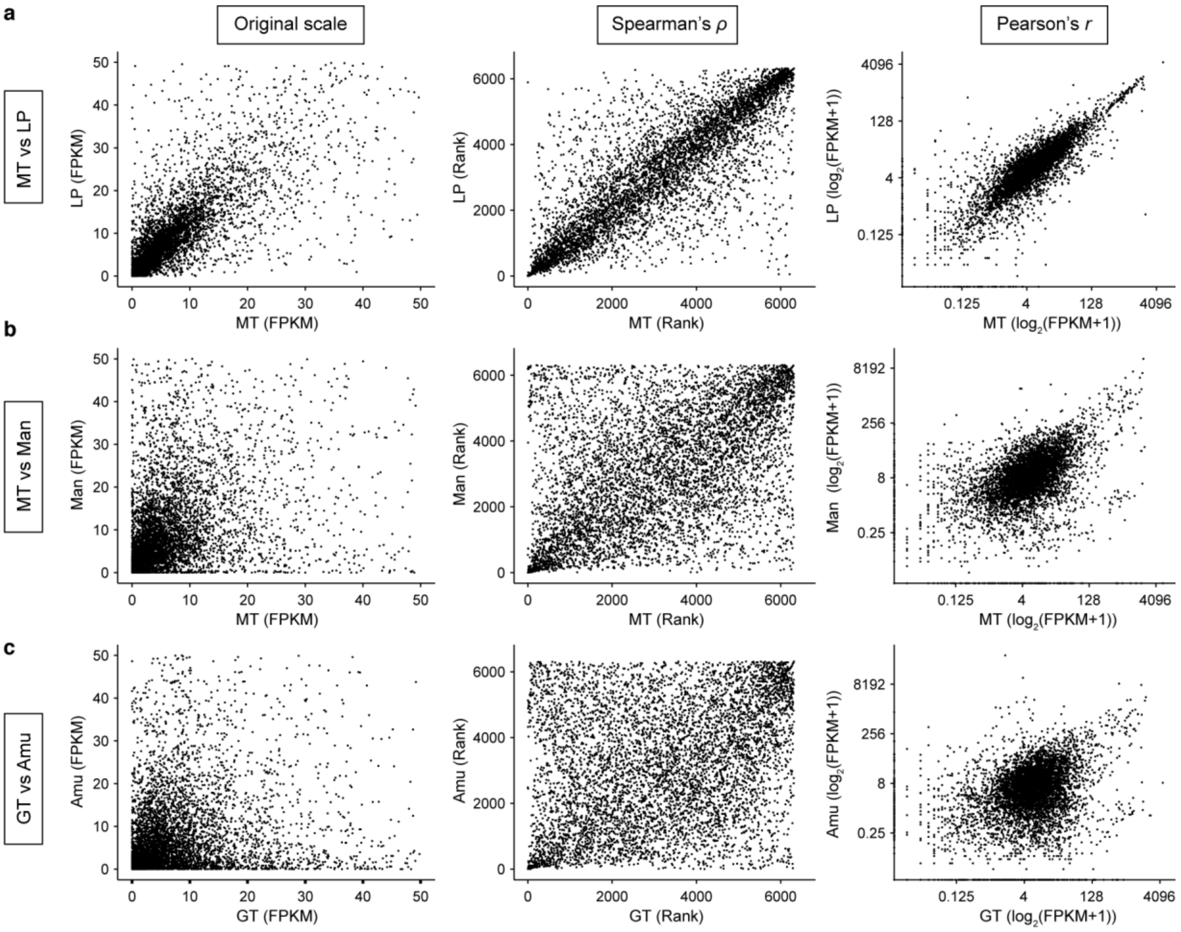

Supplementary Figure 16 | Comparison of Spearman's (*ρ*) and Pearson's (*r*) correlation coefficients.

Comparison of expression level of transcripts was plotted on the scale of fragments per kilobase of transcript per million mapped reads (FPKM) between two given tissues (original scale). Data set were transformed into rank value for Spearman's *ρ* and log transformed into $\log_2$(FPKM+1) for Pearson's *r*. Both Spearman's *ρ* and Pearson's *r* show the same correlation trend. (**a**) High correlation (*ρ*, 0.84; *r*, 0.87) of intra-species comparisons between *Lingula* mantle (MT) and lophophore (LP). (**b**) Moderate correlation (*ρ*, 0.50; *r*, 0.53) of interspecific comparisons between *Lingula* (MT) and *Crassostrea* (Man) mantles. (**c**) Low correlation (*ρ*, 0.32; *r*, 0.36) of interspecific comparison betweens *Lingula* gut (GT) and *Crassostrea* adductor muscle (Amu).

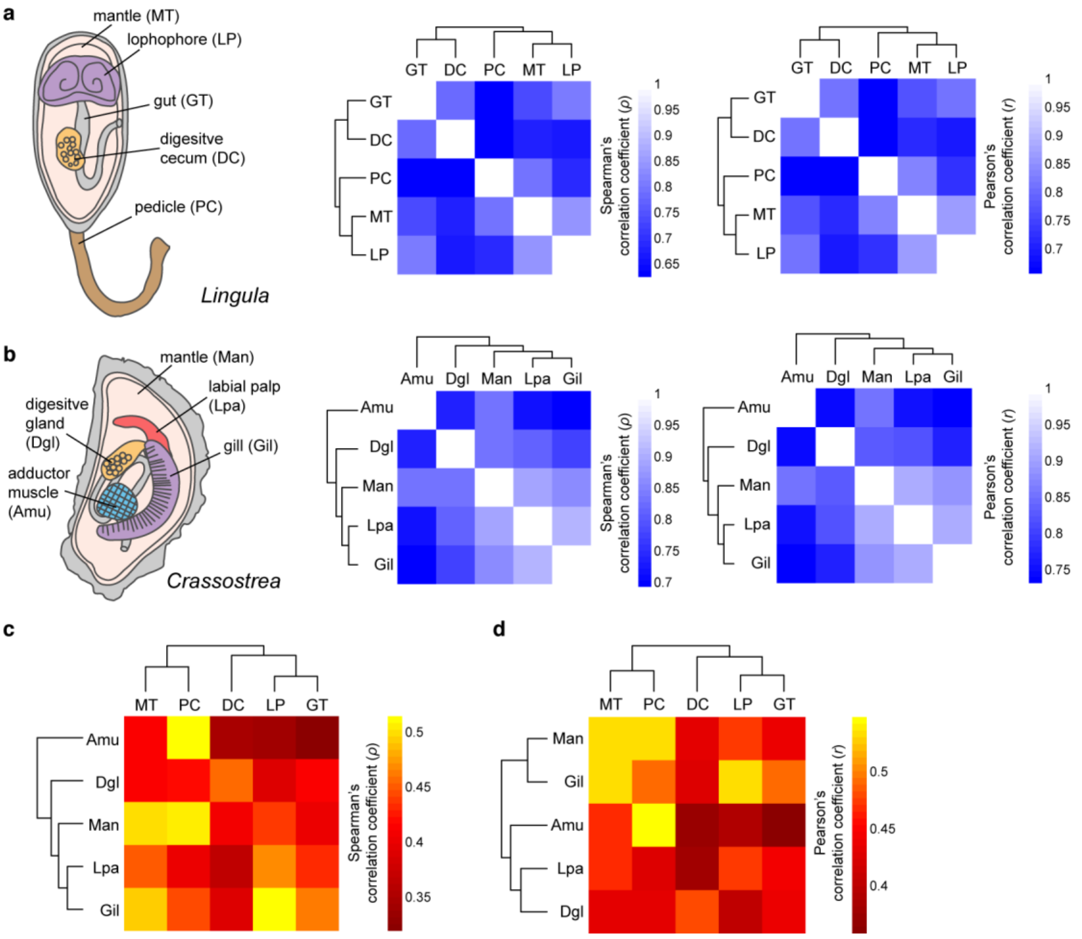

Supplementary Figure 17 | Transcriptome similarities in *Lingula* and *Crassostrea*. Intraspecific transcriptome similarities shown by Spearman's (ρ) and Pearson's (r) correlation coefficients within (**a**) *Lingula* and (**b**) *Crassostrea* tissues, respectively. *Lingula* adult is shown with the dorsal shell removed, and the anus opening to the right side. Interspecific transcriptome data analyzed by Spearman's ρ (**c**) and Pearson's r (**d**) among *Lingula* and *Crassostrea* adult tissues, in which total numbers of 6,315 orthologous gene pairs were identified.

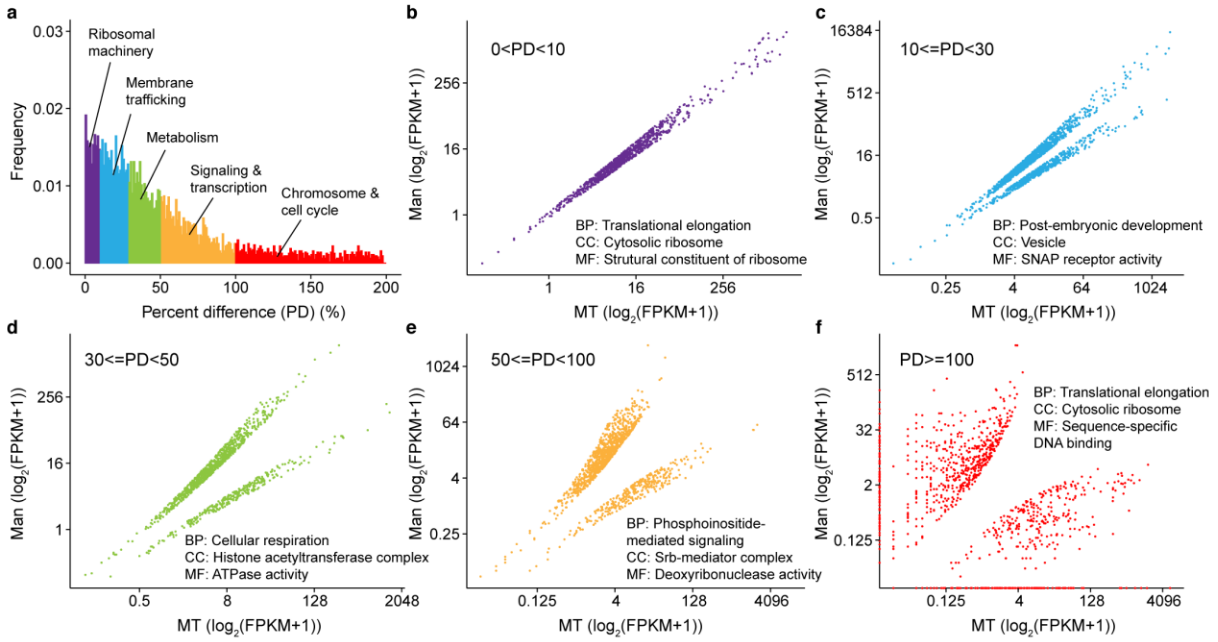

Supplementary Figure 18 | Classification of transcriptome similarities between *Lingula* and *Crassostrea* mantles.

(**a**) Distribution of percent difference (PD) of transcript expression level between *Lingula* (MT) and *Crassostrea* (Man) mantles. Groups of orthologous gene pairs in different PD ranges are classified functionally. (**b-f**) Comparison of log-transformed expression levels with different range of PD. Top one GO terms for biological process (BP), cellular component (CC), and molecular function (MF) by GO enrichment analyses are shown.

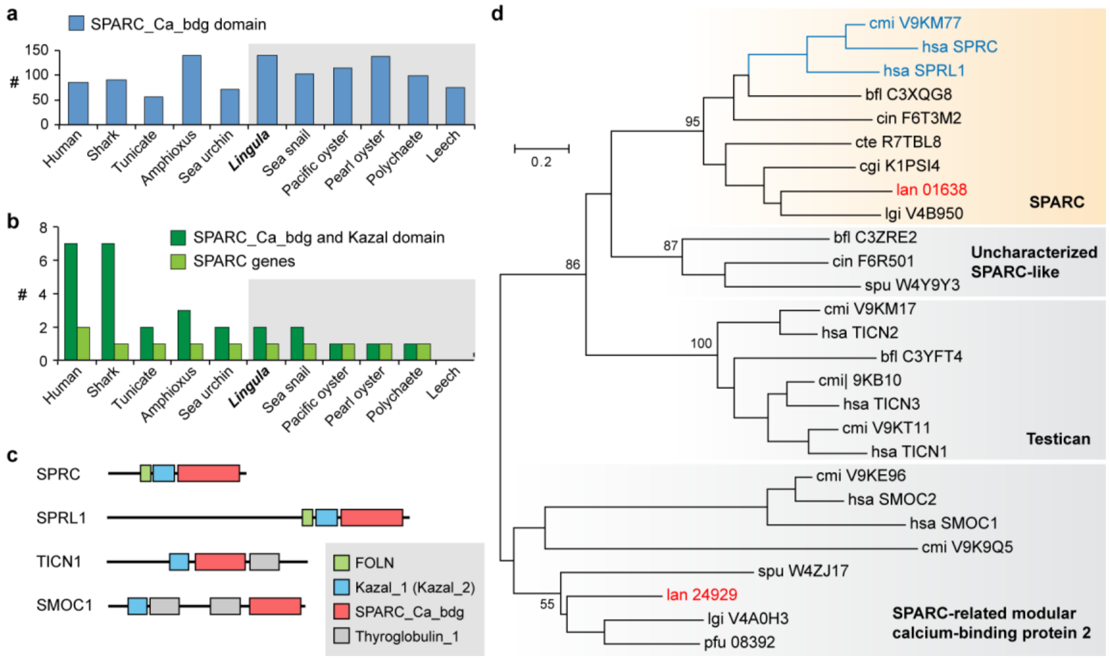

Supplementary Figure 19 | Evolution of SPARC-related genes in *Lingula*.

(**a**) Number of genes with secreted acidic proteins rich in cysteine Ca-binding region domains (SPARC_Ca_bdg, PF10591) in metazoan genomes. Grey box denotes lophotrochozoans. (**b**) Number of proteins with a combination of SPARC_Ca_bdg and Kazal-type serine protease inhibitor domains (Kazal_1, PF00050) (dark green). Number of SPARC genes identified with the BBH approach (light green). (**c**) Domain composition of SPARC-related genes. UniProt ID: SPRC, SPARC; SPRL1, SPARC-like protein 1; TICN1, Testican-1; SMOC1, SPARC-related modular calcium-binding protein 1. Pfam domain: FOLN, Follistatin/Osteonectin-like EGF domain (PF09289); Thyroglobulin_1, Thyroglobulin type-1 repeat (PF00086). (**d**) Phylogeny of SPARC-related genes constructed with 27 genes, Kazal and SPARC_Ca_bdg domains (160 amino acids) using the neighbor-joining method with the JTT model (1,000 bootstrap replicates). Vertebrate lineage with a duplication event of the SPARC gene is labeled in blue. Numbers at the nodes indicate bootstrap support values. Three-letter code: hsa, humans (*Homo sapiens*); cmi, elephant shark (*Callorhinchus milii*); cin, tunicate (*Ciona intestinalis*); bfl, amphioxus (*Branchiostoma floridae*); spu, sea urchin (*Strongylocentrotus purpuratus*); lan, brachiopod (*Lingula anatina*); lgi, sea snail (*Lottia gigantea*); cgi, Pacific oyster (*Crassostrea gigas*); pfu, pearl oyster (*Pinctada fucata*); and cte, polychaete (*Capitella teleta*).

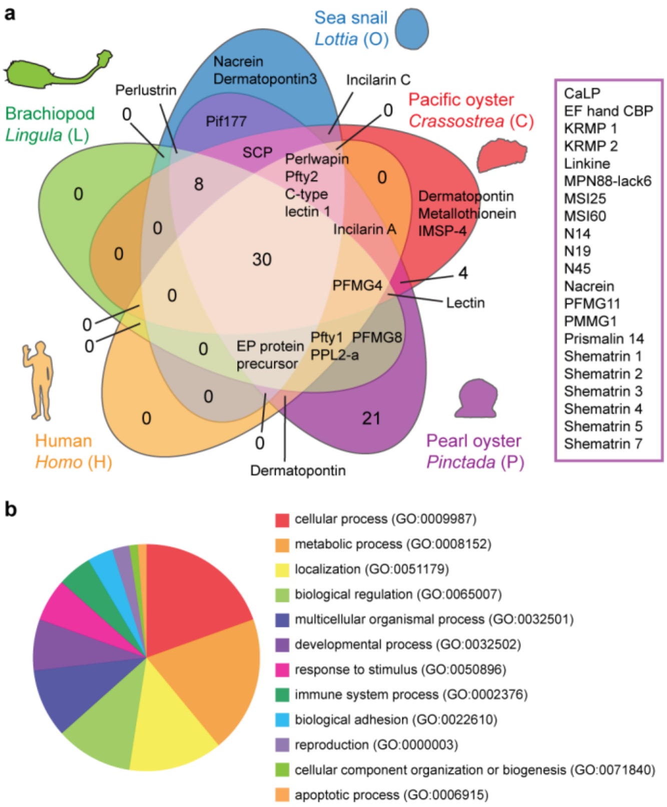

Supplementary Figure 20 | Comparison of gene sets involved in mollusc shell formation. (**a**) Known shell formation-related genes in selected bilaterians compared in a Venn diagram. Most of the genes can be found in both *Lingula* and humans, suggesting that they have general functions other than shell formation. Most of the known shell formation genes came from studies of the pearl oyster. The 22 pearl oyster specific shell formation genes are listed in the purple box. (**b**) Functional classification of GO biological process of 30 genes shared by all selected genomes. These are mainly involved in cellular and metabolic processes and with other diverse functions not limited to biomineralization, suggesting that these genes may have been co-opted independently in each mollusc lineage. See Supplementary Tables 23 and 24 for the detailed list of 45 genes found in *Lingula* and their expression profiles.

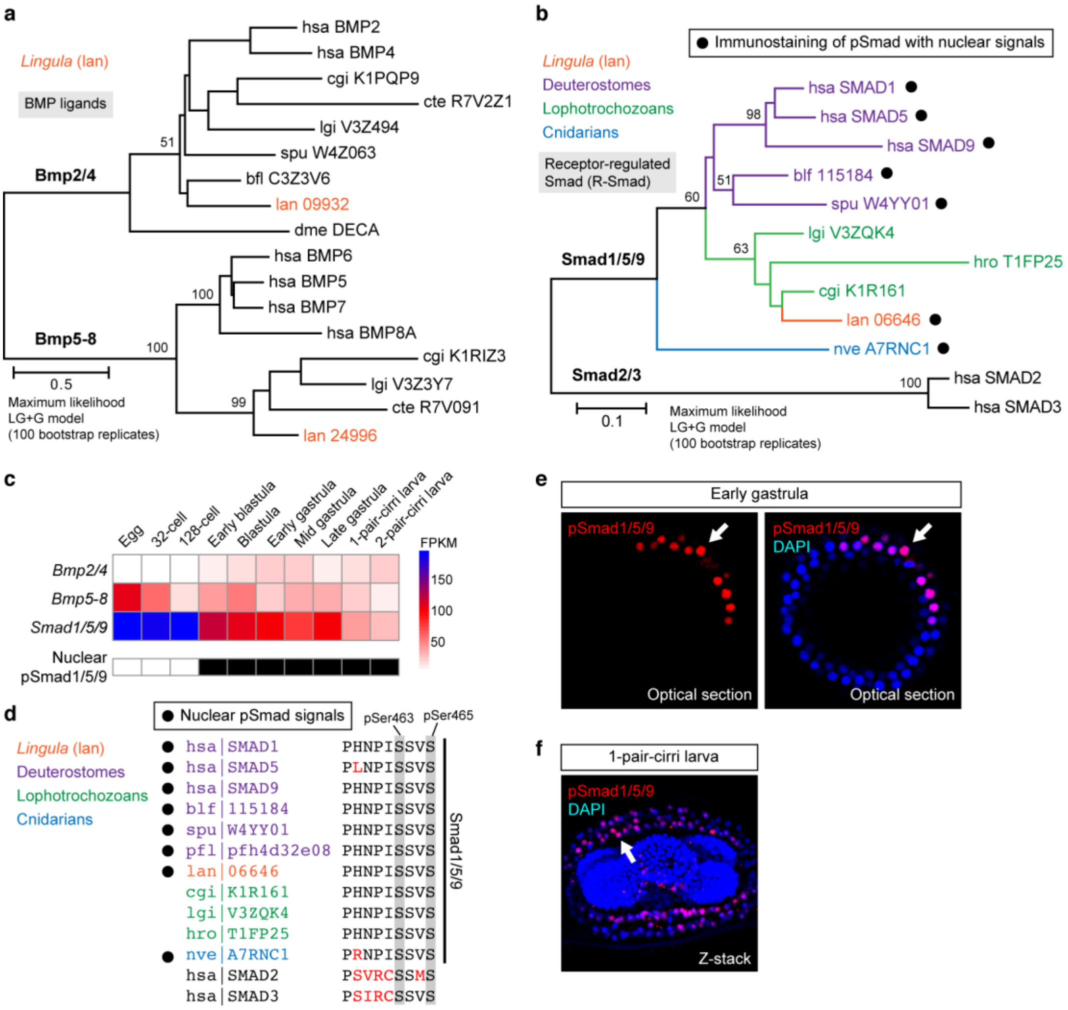

Supplementary Figure 21 | BMP signaling components in *Lingula*.

(**a**) Phylogeny of BMP ligands using 17 genes (364 amino acids). Three-letter code: hsa, humans (*Homo sapiens*); bfl, amphioxus (*Branchiostoma floridae*); spu, sea urchin (*Strongylocentrotus purpuratus*); lgi, sea snail (*Lottia gigantea*); cgi, Pacific oyster (*Crassostrea gigas*); cte, polychaete (*Capitella teleta*); dme, fruit fly (*Drosophila melanogaster*). Proteins are identified by their UniProt IDs. Numbers at the nodes indicate bootstrap support values. (**b**) Phylogeny of receptor-regulated Smad constructed with 12 genes (431 amino acids). The amphioxus sequence is from JGI. hro, leech (*Helobdella robusta*); nve, sea anemone (*Nematostella vectensis*). (**c**) Expression profiles of BMP signaling ligands and mediators. Appearance of nuclear phosphorylated Smad1/5/9 (pSmad) signals is shown in black rectangles. (**d**) Alignment of C-terminus of Smad proteins. Phosphorylated sites of Ser463/465 in human SMAD5 are shaded in grey. Different amino acids compared to SMAD1 are labeled in red. pfl, hemichordate (*Ptychodera flava*; EST ID)[4]. (**e**) Immunostaining of pSmad in early gastrula shows signals with asymmetrical nuclear localization (arrows). Nuclei are labeled with DAPI. (**f**) Nuclear signals of pSmad (arrow) in 1-pair-cirri larva from Fig. 5e without CellMask staining.

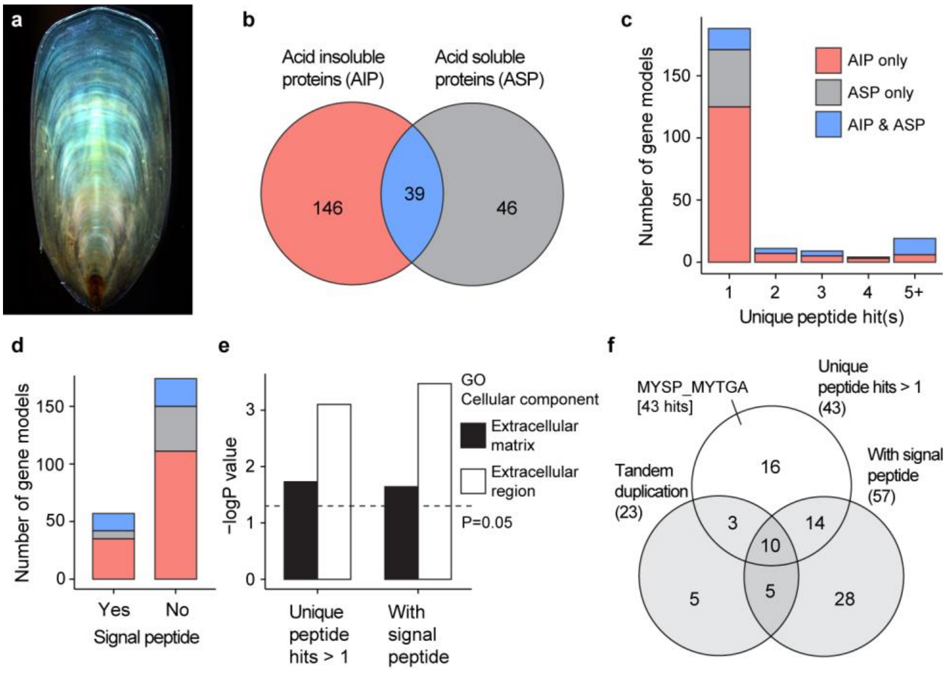

Supplementary Figure 22 | Classification and identification of *Lingula* shell matrix proteins (SMPs).

(**a**) The dissected Lingula shell with tissues removal. Note the growth rings and transparent texture. (**b**) The number of putative SMPs recovered from the acid insoluble or soluble fractions. (**c**) The number of putative SMPs with unique peptide hit(s). (**d**) The number of putative SMPs with signal peptides. (**e**) Statistical overrepresentation test of GO cellular component by PANTHER. (**f**) Selection of the final SMP set, those having multiple unique peptide hits, containing signal peptides, and showing tandem duplication on the scaffold (grey area, 65 genes). Note that a mollusc paramyosin gene (MSYP_MYTGA with 43 unique peptide hits) and cytoplasmic genes are included in the white area. These are considered as contaminants and are excluded from the final SMP set.

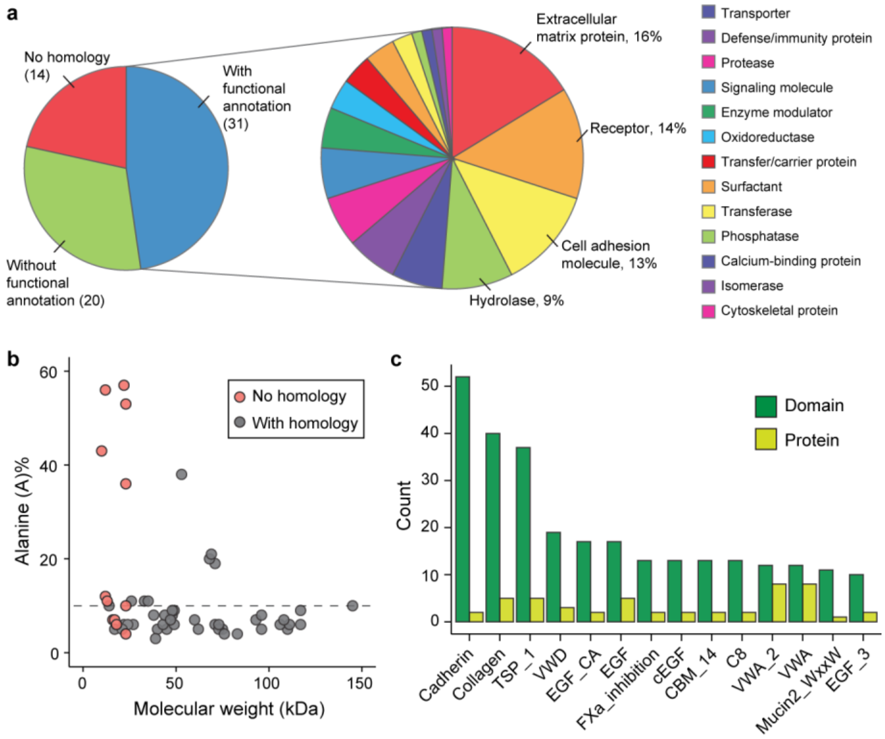

Supplementary Figure 23 | Characterization of *Lingula* SMPs.

(**a**) Distribution of functional classifications of 65 SMPs. Biological processes are shown for the 31 SMPs that have functional annotation data. (**b**) Distribution of alanine composition and molecular weight of *Lingula* SMPs. Seven SMPs with molecular weights greater than 150 kDa are not shown here. The dashed line indicates the 10% in terms of alanine content. (**c**) Top 20% domain distribution of SMPs with significant Pfam hits. Dark green, total number of a detected domains in the SMPs; light green, number of SMPs with that domain shown below. TSP_1, thrombospondins 1; VWD, von Willebrand factor type D domain; EGF_CA, calcium-binding EGF domain; CBM, carbohydrate-binding module; C8, 8 conserved cysteine residues; VWA, Von Willebrand factor type A domain.

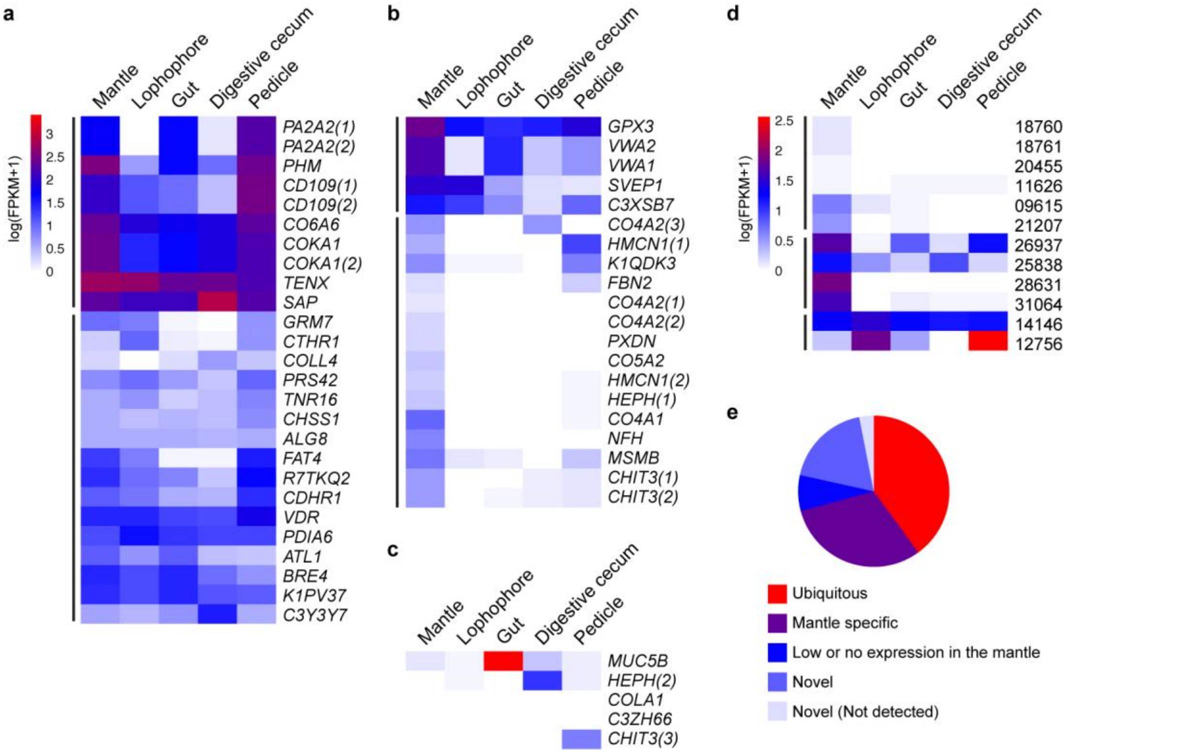

Supplementary Figure 24 | Expression of SMPs in the adult tissues.

(**a**) Expression profile of SMPs with detectable homology, expressed ubiquitously in adult tissues. Vertical lines, clustered groups based on expression pattern. Paralogs are marked by parentheses by number according to the listed order in Supplementary Table 25. (**b**) Expression profile of SMPs with detectable homology, expressed highly or specifically in mantle tissue. (**c**) Expression profiles of SMPs with detectable homology, expressed weakly in mantle tissue. (**d**) Expression profiles of SMPs without detectable homology (novel) shown among *Lingula* gene models. (**e**) Summary of the expression of SMPs. FPKM, fragments per kilobase of transcript per million mapped reads. Gene names are the human entry names in UniProt.

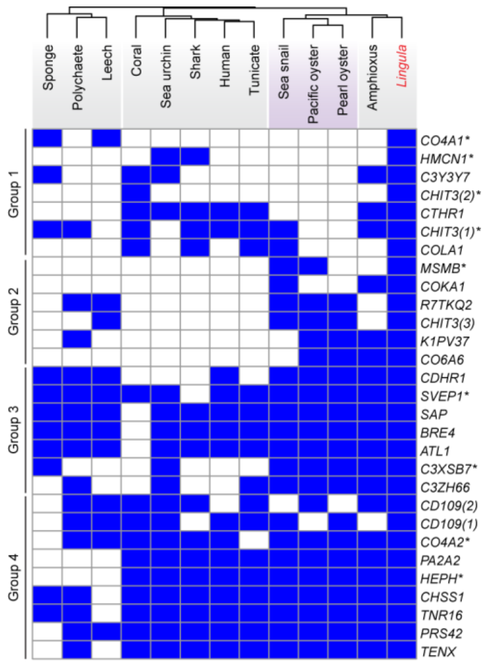

Supplementary Figure 25 | Comparative genomics of *Lingula* SMP genes.

Genomic scale comparative matrix among thirteen metazoan genomes with hierarchical clustering indicating the presence (blue) or absence (white) of 29 *Lingula* SMPs obtained with a BBH approach. Numbers of paralogs are shown in parentheses. Fourteen SMPs without detectable homology, eleven SMPs without BBH correspondence, and eleven SMPs shared by all metazoans were removed from this analysis. The closely related group by clustering is highlighted in purple. Genes highly or specifically expressed in mantle are labeled with asterisks.

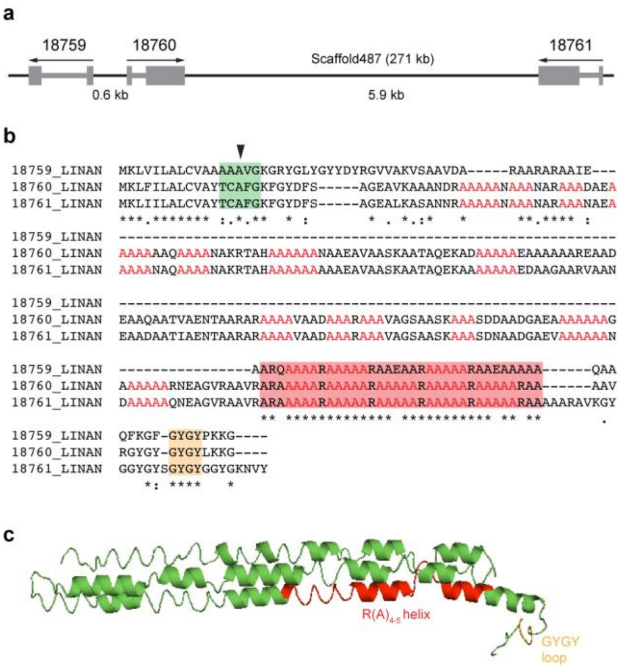

Supplementary Figure 26 | A tandem duplication of novel genes for SMPs.

(**a**) An example of tandem duplicated SMPs. The gene orientation (arrows) and the distance among genes in scale on the scaffold are shown. Grey boxes, exons. (**b**) Multiple alignments were conducted with Clustal Omega. Conserved poly-alanine (>3) is colored in red. Green box, signal peptide predicted by SiganlP where the arrowhead indicates the cleavage site. Red box, conserved $R(A)_{4\text{-}5}$ domain. Orange box, conserved GYGY motif. Asterisks, fully conserved; colons, strongly similar; periods, weakly similar. (**c**) Predicted three-helix bundle structure of gene model 18761_LINAN by I-TASSER (estimated TM-score, 0.4; RMSD, 12.2 Å) with a TM-score 0.795 to computationally designed three helix bundle (PDB ID: 4TQL). A TM-score >0.5 indicates a model of correct topology not coming from a random similarity. Conserved $R(A)_{4\text{-}5}$ helix and GYGY loop are colored in red and orange, respectively.

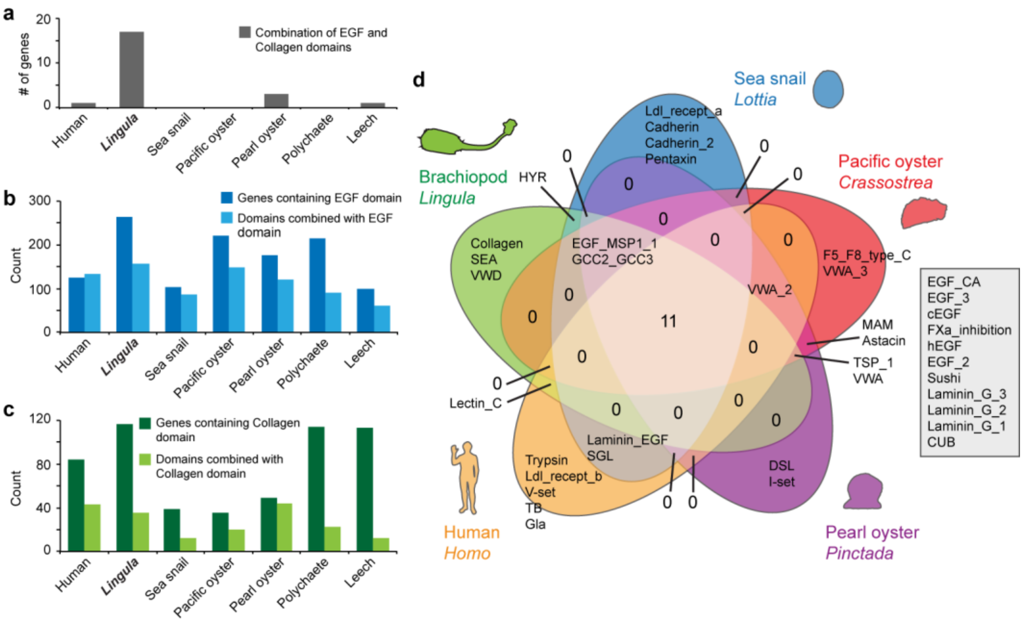

Supplementary Figure 27 | Domain shuffling of EGF and collagen domains in bilaterians.

(**a**) The number of genes with EGF and Collagen domain combinations in lophotrochozoans and humans. (**b**) The number of genes with EGF domains and the number of domains combined with EGF domains. (**c**) The number of genes with collagen domains and the number of domains combined with collagen domains. (**d**) A Venn diagram comparing the 20 most abundant domains combined with EGF domains. Note that the combination of EGF and collagen domains is abundant only in *Lingula*. Domains commonly combined with EGF domains shared in the five genomes are listed in the grey box.

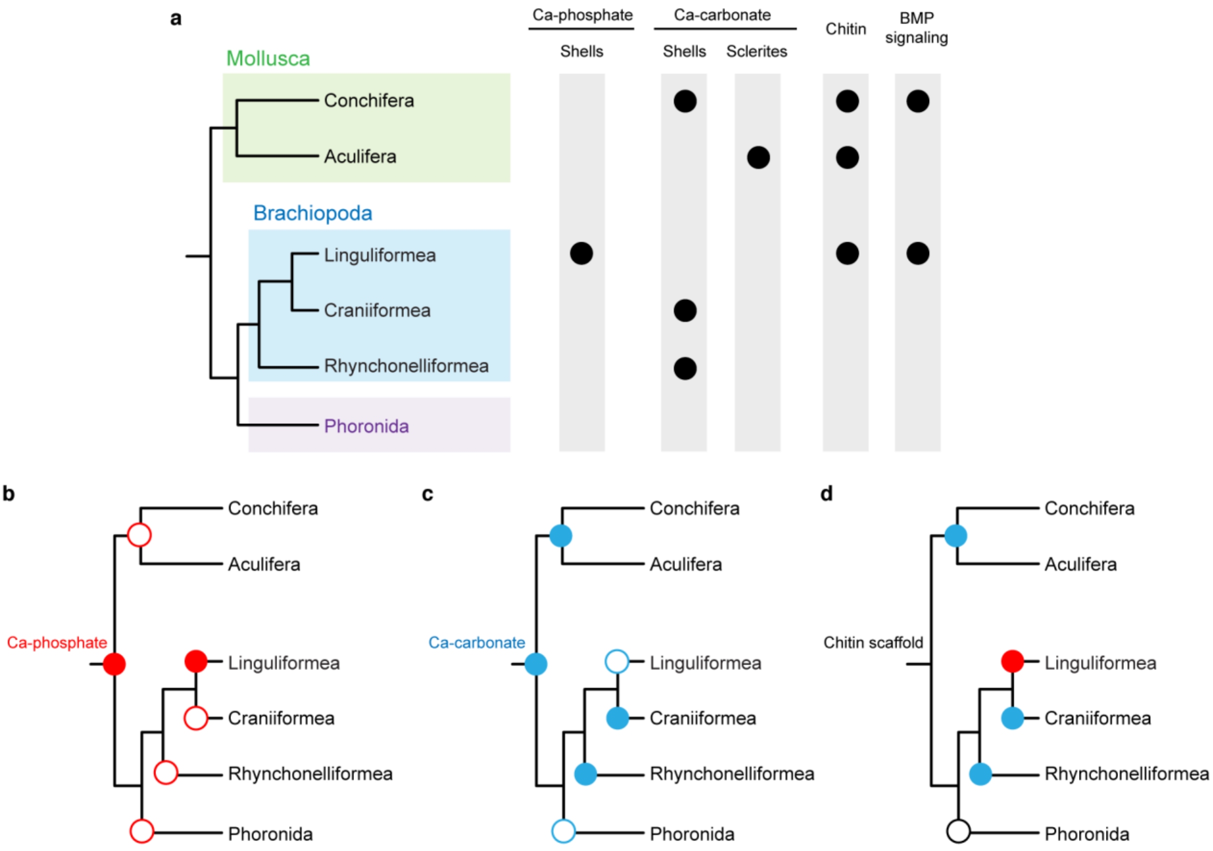

Supplementary Figure 28 | Possible evolutionary scenarios for the origins of animal biomineralization.

(**a**) Features of biomineralization in brachiopods and molluscs. Phylogeny of Mollusca (the clade Conchifera includes Gastropoda, Bivalvia, Scaphopoda, Cephalopoda, and Monoplacophora; the clade Aculifera includes Neomeniomorpha, Chaetodermomorpha, and Polyplacophora) is based on Kocot et al. (2011)[5] and Smith et al. (2011)[6]. Phylogeny of Brachiopoda and Phoronida is based on Sperling et al. (2011)[7]. The close relationship of Brachiopoda and Mollusca is supported by this study. Ca, calcium. (**b**) Ca-phosphate primitive hypothesis. Red solid circles indicate the presence of Ca-phosphate biominerals. Red open circles denote the absence of Ca-phosphate biominerals. (**c**) Ca-carbonate primitive hypothesis. Blue solid circles indicate the presence of Ca-carbonate biominerals. Blue open circles denote absent of Ca-carbonate biominerals. (**d**) Chitin scaffold hypothesis. Chitin is detected in both shells and sclerites in molluscs as well as in brachiopod shells. Black open circle indicates no biomineral.

Supplementary Tables

Supplementary Table 1 | Proteomes and genome assemblies used in comparative analyses

Three -letter code	Species name	Common name	Proteome source[a]	Genome assembly source	Genome file	Genome annotation source	GFF file
adi	*Acropora digitifera*	Coral	OIST*	OIST	adi20110501_Scaffold.fa	OIST	aug_repeatmask_pasa_input.gff3
aqu	*Amphimedon queenslandica*	Sponge	UniProt*	Ensembl	Amphimedon_queenslandica.Aqu1.21.dna_rm.genome.fa	NCBI	ref_v1.0_scaffolds.gff3
bfl	*Branchiostoma floridae*	Amphioxus	UniProt*	JGI	Branchiostoma.allmasked	JGI	Brafl1.FilteredModels1.gff
cel	*Caenorhabditis elegans*	Nematode	UniProt*	NA	NA	WormBase	c_elegans.WS236.annotations.gff3
cgi	*Crassostrea gigas*	Pacific oyster	UniProt*	OysterDB	scaffold.fa	OysterDB	oyster.v9.glean.final.rename.gff
cin	*Ciona intestinalis*	Tunicate	UniProt*	JGI	ciona.rm.fasta	NCBI	ref_KH_scaffolds.gff3
cmi	*Callorhynchus milii*	Elephant shark	UniProt*	NA	NA	NA	NA
cte	*Capitella teleta*	Polychaete	UniProt*	JGI	Capitella_spI.allmasked	JGI	FilteredModelsv1.0.gff
dme	*Drosophila melanogaster*	Fruit fly	UniProt*	NA	NA	FlyBase	dmel-all-no-analysis-r5.55.gff
dpu	*Daphnia pulex*	Water flea	UniProt*	NA	NA	NA	NA
dre	*Danio rerio*	Zebrafish	UniProt*	NA	NA	NCBI	ref_Zv9_scaffolds.gff3
gga	*Gallus gallus*	Chicken	UniProt*	NA	NA	NA	NA
hro	*Helobdella robusta*	Leech	UniProt*	JGI	Helobdella_robusta.allmasked	JGI	Helobdella_robusta_FilteredModels3.gff
hsa	*Homo sapiens*	Human	UniProt*	NA	NA	NCBI	ref_GRCh37.p13_scaffolds.gff3
lan	*Lingula anatina*	Brachiopod (Lamp shell)	This study*	This study	This study	This study	This study
lgi	*Lottia gigantea*	Sea snail (Limpet)	UniProt*	JGI	Lotgi1_assembly_scaffolds_repeatmasked.fasta	JGI	Lotgi1_GeneModels_FilteredModels1.gff
NA	*Mnemiopsis leidyi*	Ctenophore (Comb jelly)	NA	NHGI	MlScaffold09.nt	NA	NA
NA	*Trichoplax adhaerens*	Trichoplax	NA	JGI	Triad1_masked_genomic_scaffolds.fasta	NA	NA
nve	*Nematostella vectensis*	Sea anemone	UniProt*	JGI	Nemve1.allmasked	JGI	Nemve1.FilteredModels1.gff
pfu	*Pinctada fucata*	Pearl oyster	OIST*	OIST	pfu_genome1.0.fasta	NA	NA
sma	*Schistosoma mansoni*	Blood fluke	UniProt*	NA	NA	NA	NA
spu	*Strongylocentrotus purpuratus*	Purple sea urchin	UniProt*	SpBase	Spur_v2.1.assembly.all.fa	NCBI	ref_Spur_3.1_scaffolds.gff3
tca	*Tribolium castaneum*	Red flour beetle	UniProt*	NA	NA	NA	NA
xtr	*Xenopus tropicalis*	Frog	UniProt*	NA	NA	JGI	Xentr4_FilteredModels1.gff

[a]Proteomes used for OrthoMCL analysis are labeled with asterisks (*). *Lingula* is highlighted in grey. NA, not analyzed in this study.

Supplementary Table 2 | Scaffold assembly of the *Lingula* genome

Sequencing Platform	Method	Library length	Read length[a]	Raw reads/ read pairs[b]	Raw bases	Total number of scaffolds	Scaffold N50[c]
Roche 454	Single end	1,750	520	18,515,644	9,620,324,824	33,571	18,305
Illumina MiSeq	Paired-end	500	2x250	2,023,980	1,008,114,613	33,331	18,464
Illumina MiSeq	Paired-end	605	2x250	50,998,885	26,383,588,510	25,558	26,115
Illumina MiSeq	Paired-end	620	2x250	10,392,969	5,132,085,210	23,968	28,603
Illumina MiSeq	Mate pair (Cre-LoxP)	1,500	2x250	32,904,606	15,937,776,007	19,218	39,316
Illumina HiSeq	Mate pair (Cre-LoxP)	3,000	2x150	4,788,702	1,446,188,004	18,589	41,163
Illumina MiSeq	Mate pair (Nextera)	5,000	2x300	4,282,447	2,414,185,960	14,975	57,618
Illumina MiSeq	Mate pair (Nextera)	8,500	2x300	17,686,389	10,202,384,389	9,798	113,603
Illumina MiSeq	Mate pair (Nextera)	17,000	2x300	25,559,146	15,503,641,744	7,256	198,916
PacBio RS II	SMRT	>7,000	7,000	1,184,610	8,476,672,528	4,466	296,446

[a]454 and PacBio, mean read length; MiSeq, maximal read cycle. [b]Single end reads for 454 and PacBio; Paired end read pairs for Miseq and HiSeq. [c]The number of scaffold N50 is before gap closing.

Supplementary Table 3 | Genome assembly statistics of *Lingula* and selected marine invertebrates

Common name	Species name	Assembly statistics							CEGMA[b] (%)		Reference
		Ver[a]	Size (Mb)	Methods	Depth	Assembler	Contig N50	Scaffold N50	C	P	
Brachiopod	*Lingula anatina*	1.0	425	454, MiSeq, HiSeq, PacBio	~226x	Newbler	55 kb	294 kb	85	98	This study
Sea snail (Limpet)	*Lottia gigantea*	1.0	348	Sanger	~9x	JAZZ	96 kb	1,870 kb	86	98	Simakov et al., 2013[8]
Pacific oyster	*Crassostrea gigas*	1.0	559	HiSeq	~155x	SOAPdenovo	19 kb	401 kb	79	95	Zhang et al., 2012[9]
Pearl oyster	*Pinctada fucata*	1.0	1,413	454, GAIIx	~40x	Newbler	1.7 kb	15 kb	25	63	Takeuchi et al., 2012[10]
Polychaete	*Capitella teleta*	1.0	324	Sanger	~8x	JAZZ	22 kb	188 kb	94	97	Simakov et al., 2013[8]
Tunicate	*Ciona intestinalis*	1.0	117	Sanger	~8.5x	JAZZ	37 kb	203 kb	88	96	Dehal et al., 2002[11]
Amphioxus	*Branchiostoma floridae*	1.0	522	Sanger	~11.5x	JAZZ	26 kb	1,584 kb	81	98	Putnam et al., 2008[12]
Sea urchin	*Strongylocentrotus purpuratus*	2.1	814	Sanger	~8x	Atlas-wgs	12 kb	123 kb	60	95	Sodergren et al. 2006[13]
Coral	*Acropora digitifera*	1.0	419	454, GAIIx	~150x	Newbler	11 kb	192 kb	48	82	Shinzato et al., 2011[14]
Sea anenome	*Nematostella vectensis*	1.0	450	Sanger	~6.5x	JAZZ	20 kb	472 kb	73	95	Putnam et al., 2007[15]
Placozoan	*Trichoplax adhaerens*	1.0	98	Sanger	~8x	JAZZ	204 kb	5,978 kb	94	96	Srivastava et al., 2008[16]
Sponge	*Amphimedon queenslandica*	1.2	167	Sanger	~9x	custom[c]	11 kb	120 kb	79	94	Srivastava et al., 2010[17]
Ctenophore	*Mnemiopsis leidyi*	1.0	156	454, GAIIx	~160x	Phusion	30 kb	187 kb	78	92	Ryan et al., 2013[18]
Ctenophore	*Pleurobrachia bachei*	1.1	160	454, GaIIx, HiSeq, MiSeq	~700x	Velvet, SOAPdenovo, ABySS, Newbler	NA	24 kb	NA	NA	Moroz et al., 2014[19]

Lingula in this study is highlighted in grey. [a]Version of the genome assembly at the published time. [b]Completeness of genome assembly is assessed with Core Eukaryotic Genes Mapping Approach (CEGMA) analysis using complete gene models (C) or partial gene models (P). [c]Custom approach by using MALIGN and phrap. NA, not available.

Supplementary Table 4 | Summary of RNA-seq samples and read numbers before and after quality filtering

Sample	Label	Description	Raw Read Pairs	Both Surviving (Q20)[a]	Survival rate
Embryo	F_egg	Fertilized egg	9,198,361	6,522,831	70.91%
	32-128	32-cell to 128-cell	9,813,670	6,902,404	70.33%
	128-EB	128-cell to early blastula	8,993,242	6,323,987	70.32%
	EB	Early blastula	12,205,395	8,529,267	69.88%
	B	Blastula	9,361,241	6,593,472	70.43%
	EG	Early gastrula	19,149,847	13,349,281	69.71%
	MG	Mid gastrula	11,846,791	8,268,875	69.80%
	LG	Late gastrula	18,019,653	12,608,968	69.97%
	1PCL	1-pair-cirri larva	38,271,682	26,537,318	69.34%
	2PCL	2-pair-cirri larva	11,895,218	8,228,684	69.18%
Adult tissue	Lophophore	Lophophore	25,123,284	23,494,368	93.52%
	Gut	Whole gut tissue	27,755,664	26,075,471	93.95%
	Liver	Digestive cecum	44,937,346	42,505,445	94.59%
	D-mantle	Dorsal mantle	31,157,818	28,879,015	92.69%
	V-mantle	Ventral mantle	33,717,677	31,596,371	93.71%
	Tail	Pedicle	33,928,166	31,676,277	93.36%
	R-tail	Regenerated pedicle	23,979,080	22,525,047	93.94%
Total			369,354,135	310,617,081	84.10%

[a]Q20, Phred quality score 20 (99% base call accuracy). Read length, 100 bp.

Supplementary Table 5 | Summary of debated phylogenetic positions of lophotrochozoan phyla

Hypothesis	Proposed relationship (Newick format)	Genes used for analyses	Taxa included	Analytical methods[a]	Reference
ND	(Brachiopoda,Nemertea);	LSU + SSU + mitochondrial genomes + 8 nuclear protein coding genes	168	Bayesian (GTR+Γ model)	Bourlat et al. 2008[1]
((B,A),M);	((((Brachiopoda,Phoronida),Nemertea),Annelida),Mollusca);	150 genes (110 non-ribosomal + 40 ribosomal)	77(64)	Bayesian (CAT model)	Dunn et al. 2008[20]
((B,A),M);	((((Brachiopoda,Phoronida),Nemertea),Annelida),Mollusca);	79 ribosomal genes	39	ML (rtRev+Γ+F model)	Helmkampf et al. 2008[21]
((B,M),A);	((((Brachiopoda,Phoronida),Mollusca),Annelida),Nemertea);	11 protein coding genes + 2 ribosomal RNA genes	96	ML (GTR+Γ+I model)	Paps et al. 2009a[22]
((B,M),A);	((((Brachiopoda,Phoronida),Mollusca),Annelida),Nemertea);	LSU and SSU rDNAs	22	ML (GTR+Γ+I model)	Paps et al. 2009b[23]
((B,M),A);	(((Brachiopoda,Nemertea),Mollusca),Annelida);	1,487 genes (only 2 from Phoronid)	94	ML (rtRev model)	Hejnol et al. 2009[2]
((B,A),M);	((((Brachiopoda,Phoronida),Nemertea),Annelida),Mollusca);	78 ribosomal genes	62	ML (mixed 14 models)	Hausdorf et al. 2010[24]
(B,(M,A));	(((Brachiopoda,Phoronida),(Annelida,Mollusca)),Nemertea);	7 nuclear housekeeping genes + 3 ribosomal genes + specific microRNAs	72	Bayesian (GTR+Γ model)	Sperling et al. 2011[7]
((B,M),A);	((((Brachiopoda,Phoronida),Mollusca),Annelida),Nemertea);	7 nuclear housekeeping genes + 3 ribosomal genes	113	Bayesian (GTR+Γ model)	Erwin et al. 2011[25]
((B,M),A);	(((Mollusca,Brachiopoda),Nemertea),Annelida);	232 genes	63	ML (LG+I+Γ model)	Struck et al. 2014[3]
ND	((Mollusca,Annelida),Nemertea);	2,779 genes	20	ML (LG+Γ model)	Andrade et al. 2014[26]

[a]ML, maximum likelihood. B, Brachiopoda; M, Mollusca; A, Annelida; ND, the relationship among Brachiopoda, Mollusca, and Annelida is not determined.

Supplementary Table 6 | Number of genes containing domains lost in the annelid lineage

Pfam domain name	Pfam ID	Function	hsa	bfl	lan	lgi	cgi	pfu	cte	hro	tca	dpu
3-PAP	PF12578	Myotubularin-associated protein	3	1	1	1	1	1	0	0	2	2
Alpha-2-MRAP_C	PF06401	Alpha-2-macroglobulin RAP, C-terminal domain	1	1	3	1	1	1	0	0	2	1
DAP	PF15228	Death-associated protein	2	1	1	1	1	1	0	0	1	2
DUF1903	PF08991	Domain of unknown function (DUF1903)	1	1	1	1	1	1	0	0	1	1
DUF2356	PF10189	Conserved protein (DUF2356)	1	2	1	1	1	1	0	0	1	1
DUF2368	PF10166	Uncharacterised conserved protein (DUF2368)	1	2	1	1	1	1	0	0	1	1
DUF3697	PF12478	Ubiquitin-associated protein 2	2	1	1	1	1	1	0	0	1	1
DUF4625	PF15418	Domain of unknown function (DUF4625)	2	1	3	2	2	1	0	0	1	1
FNIP_N	PF14636	Folliculin-interacting protein N-terminus	2	1	1	1	1	1	0	0	1	1
Glyco_hydro_30	PF02055	O-Glycosyl hydrolase family 30	1	2	3	2	4	3	0	0	4	3
PA14	PF07691	PA14 domain	3	5	3	1	4	6	0	0	0	2
ParBc	PF02195	ParB-like nuclease domain	1	1	2	1	1	1	0	0	1	5
Peptidase_M23	PF01551	Peptidase family M23	1	5	1	2	2	1	0	0	0	1
Phospholip_A2_1†	PF00068	Phospholipase A2	9	12	21	1	5	2	0	0	1	3
PTE	PF02126	Phosphotriesterase family	1	7	5	2	1	1	0	0	0	1
RasGEF_N_2	PF14663	Rapamycin-insensitive companion of mTOR RasGEF_N domain	1	1	1	1	1	1	0	0	1	1
RICTOR_M	PF14666	Rapamycin-insensitive companion of mTOR, middle domain	1	1	1	2	1	1	0	0	1	1
RNA_pol_I_A34	PF08208	DNA-directed RNA polymerase I subunit RPA34.5	2	1	2	1	1	1	0	0	0	2
SOUL	PF04832	SOUL heme-binding protein	2	20	3	9	9	7	0	0	0	5
Spot_14	PF07084	Thyroid hormone-inducible hepatic protein Spot 14	2	1	2	1	1	1	0	0	1	1
ThiG	PF05690	Thiazole biosynthesis protein ThiG	2	1	2	1	1	2	0	0	1	2
tRNA_edit	PF04073	Aminoacyl-tRNA editing domain	1	1	1	2	1	1	0	0	0	1

Highest expanded domains in *Lingula* compared to other lophotrochozoans are labeled with daggers (†). The major phyla are separated by vertical dashed lines. The numbers of *Lingula* genes are highlighted in grey. Three-letter code: hsa, human (*Homo sapiens*); bfl, amphioxus (*Branchiostoma floridae*); lan, brachiopod (*Lingula anatina*); lgi, sea snail (*Lottia gigantea*); cgi, Pacific oyster (*Crassostrea gigas*); pfu, pearl oyster (*Pinctada fucata*); cte, polychaete (*Capitella teleta*); hro, leech (*Helobdella robusta*); tca, beetle (*Tribolium castaneum*); dpu, water flea (*Daphnia pulex*).

Supplementary Table 7 | Examples of long (>4) shared syntenic blocks in *Lingula* and *Lottia*

Lingula scaffold	*Lottia* scaffold	Number of shared orthologs	Human ID or ortholog group ID[a]	Neighboring linked[b]
scaffold1	sca_1	12	OG_03361 | OG_13209 | PK3C3 | EPHX4 | SCC4 | EPHX4 | F221B | F221B | F91A1 | OG_10621 | GDF11 | IPP	No
scaffold1	sca_31	5	PSA1 | IKBP1 | JAGN1 | PARP4 | OG_17100	No
scaffold5	sca_39	5	HIRA | WDR66 | SETD6 | AN13A | GIT1	No
scaffold6	sca_18	6	PPAC2 | FA78A | NU214 | PHYD1 | ZDH12 | FBXW5	Partial
scaffold8	sca_26	5	EGR1 | TRUA | CTBP1 | MAEA | TEX36	Partial
scaffold11	sca_1	9	WDR93 | PX11B | RM54 | SPA5L | OG_10175 | NASP | BT3L4 | PIGW | RTCA	Partial
scaffold12	sca_20	5	RT23 | PAXI | GPN1 | TCPW | NCPR	No
scaffold13	sca_34	5	DAPK1 | DAPK3 | HACD3 | GALK2 | F227B	Yes
scaffold16	sca_22	5	LGMN | TYY1 | DEGS2 | NADAP | ABCBA	No
scaffold18	sca_1	13	OG_13470 | CNO11 | ZXDA | RS11 | C19L1 | DAAF3 | RAB23 | HSDL2 | CC14A | PIGB | EPT1 | DPTOR | DCC1	Partial
scaffold30	sca_125	5	S35B3 | THOC1 | BLK | GCKR | BLK	No
scaffold40	sca_142	5	ERCC1 | GNPTG | TSR3 | OG_19687 | LENG8	No
scaffold44	sca_20	6	OG_11891 | HPPD | OG_08589 | OG_07356 | OG_07835 | PKRI1	Partial
scaffold46	sca_5	6	BOLA1 | DCTN4 | NODAL | COX18 | TOB1 | DNLI1	Partial
scaffold46	sca_69	5	DDX46 | GAR1 | RHG24 | MK08 | CJ011	Partial
scaffold60	sca_8	5	DRG2 | COX11 | FSCN1 | ALKB5 | OG_08649	Partial
scaffold61	sca_2	6	FGOP2 | TM7S3 | CL029 | OSB10 | OG_09765 | OG_12760	No
scaffold61	sca_25	6	GATC | TRIA1 | BACD3 | IFT20 | BACD3 | IFT20	Partial
scaffold63	sca_37	5	SSA27 | NU133 | ARGI2 | MCM5 | EAPP	Partial
scaffold75	sca_39	5	TPC1 | BOLA2 | OG_09069 | BOLA2 | OG_09069	No
scaffold130	sca_6	5	RRP7A | NAA60 | RRP7A | NAA60 | OG_09688	Partial
scaffold131	sca_12	6	FYCO1 | MNX1 | TMUB2 | HIBCH | PPCS | FBXL2	No
scaffold140	sca_11	8	UBP36 | CYH1 | G3BP2 | RINT1 | UBC9 | RINT1 | UBC9 | FA13A	No
scaffold146	sca_31	5	ATE1 | ODBB | FA46A | ORC3 | EF2K	Partial
scaffold157	sca_50	5	VAMP3 | B3GT6 | UB2J2 | ATD3A | PK3CA	Partial
scaffold198	sca_1	6	SOX11 | CDKAL | CCD78 | HIAL1 | NANO2 | TM38B	No
scaffold198	sca_35	8	ARFRP | MBRL | BABA1 | OBRG | TERA | PTC1 | NEUL | MTAP	Partial
scaffold202	sca_12	5	WSDU1 | HXB7 | HXB5 | HXC4 | HXA1	Partial
scaffold203	sca_5	8	PIGX | CHMP6 | ATG12 | WDR16 | FOXK1 | TEKT4 | CP059 | MFS11	Yes
scaffold204	sca_1	6	TM214 | TATD1 | HPCL1 | IF2A | HAUS3 | AP1M1	No
scaffold205	sca_150	5	HEAT4 | TCRG1 | P4K2A | OG_17178 | VPS51	Partial
scaffold215	sca_6	7	ISCU | PRKN2 | SETD4 | TMEM9 | JIP1 | AT5F1 | RRAS2	Partial
scaffold226	sca_18	5	BOK | F1882 | PIGZ | NCBP2 | EFGM	No
scaffold259	sca_100	5	FACR1 | TADA3 | PIGV | PLK1 | PINX1	Partial
scaffold275	sca_1	8	S39A3 | PMGT1 | DEP1A | OSBL9 | PSB2 | MARE3 | UTP23 | EIF3H	Partial
scaffold301	sca_35	7	SKP2 | CCHL | SKP2 | CCHL | PTBP1 | METL4 | TC1D3	Partial
scaffold307	sca_25	5	LPP1 | TERB1 | RL27A | CHKA | TIF1A	No
scaffold395	sca_19	5	VMAT1 | SMOX | GRPE1 | GFOD1 | NEK11	No
scaffold415	sca_79	5	DFFB | CE104 | S12A9 | IF5A1 | OG_12542	No
scaffold458	sca_5	5	T2EA | TXLNA | HDC | SYAP1 | PSMG1	Partial
scaffold603	sca_21	9	SUV3 | SFXN1 | TM128 | CPEB2 | MARH5 | ZDH16 | MED28 | TM127 | TRFM	No
scaffold709	sca_1	5	UK114 | POP1 | UK114 | POP1 | LYPA1	No
scaffold757	sca_6	5	OG_10413 | CNO6L | RM01 | DHE3 | TSN33	Partial

[a]Ortholog group ID is given if no human ortholog can be detected. [b]Yes, all the orthologs are tightly linked; Partial, at least three orthologs are tightly linked; No, orthologs are scattered and distantly located on the corresponded scaffold.

Supplementary Table 8 | Examples of long (>4) shared syntenic blocks in *Lingula* and *Branchiostoma*

Lingula scaffold	*Branchiostoma* scaffold	Number of shared orthologs	Human ID or ortholog group ID[a]	Neighboring linked[b]
scaffold8	scaffold_232	6	RN103 | HPSE | TRUA | CTBP1 | MAEA | TEX36	Partial
scaffold14	scaffold_96	7	IPP | OG_04278 | MBOA5 | TADBP | LRC23 | CASZ1 | CASZ1	Partial
scaffold24	scaffold_24	5	OG_06707 | ARHGH | TM165 | OG_08226 | MGT4A	No
scaffold46	scaffold_9	5	BOLA1 | DCTN4 | DDX46 | GAR1 | CJ011	No
scaffold92	scaffold_46	5	IPMK | PIGF | RPAC1 | RHGBB | GRP1	No
scaffold96	scaffold_2	5	ETFD | VWA3B | CNOT7 | F16A2 | SH3R1	No
scaffold119	scaffold_165	5	KCND1 | MTU1 | MTMRE | APEX2 | PXK	Partial
scaffold141	scaffold_42	7	CA198 | PUS10 | REL | EMAL5 | NEK9 | ZC21C | MLH3	Partial
scaffold177	scaffold_347	7	DC2L1 | LRRC9 | OG_08938 | CDKN3 | OG_08938 | CDKN3 | BMP2	No
scaffold205	scaffold_205	5	NSE4A | TACC1 | TCRG1 | P4K2A | OG_17178	Partial
scaffold267	scaffold_2	5	T184C | SPG20 | UBP12 | FRG1 | PCM1	No
scaffold664	scaffold_326	5	TAF7 | NIPA2 | TFAP4 | TIM16 | LRC59	Partial
scaffold1240	scaffold_84	8	BBOF1 | S29A3 | WDR43 | WDHD1 | SOCS5 | MMSA | LIN52 | LIN52	Partial

[a]Ortholog group ID is given if no human ortholog can be detected. [b]Yes, all the orthologs are tightly linked; Partial, at least three orthologs are tightly linked; No, orthologs are scattered and distantly located on the corresponded scaffold.

Supplementary Table 9 | Examples of long (>4) shared syntenic blocks in *Lingula* and *Capitella*

Lingula scaffold	*Capitella* scaffold	Number of shared orthologs	Human ID or ortholog group ID[a]	Neighboring linked[b]
scaffold180	scaffold_5	5	ZFAT | SEH1 | NEUL2 | SEH1 | NEUL2	No
scaffold198	scaffold_547	7	CCD78 | MBRL | BABA1 | OBRG | TERA | PTC1 | NEUL	Partial
scaffold215	scaffold_1	5	SND1 | TMEM9 | JIP1 | AT5F1 | RRAS2	No
scaffold275	scaffold_1	7	MARE3 | UTP23 | EIF3H | OG_07962 | PITH1 | RM15 | OG_11917	Partial
scaffold40	scaffold_208	6	LENG8 | KAP0 | D42E1 | STPG2 | UNC5A | KCC2A	Yes
scaffold61	scaffold_315	6	TX261 | OG_00315 | BACD3 | IFT20 | BACD3 | IFT20	Partial

[a]Ortholog group ID is given if no human ortholog can be detected. [b]Yes, all the orthologs are tightly linked; Partial, at least three orthologs are tightly linked; No, orthologs are scattered and distantly located on the corresponded scaffold.

Supplementary Table 10 | Number of introns in 150 one-to-one phylogenetic markers

Gene name	lan	lgi	cte	Gene name	lan	lgi	cte	Gene name	lan	lgi	cte
AATF	14	8	11	HACD2*	7	7	6	RS8*	6	6	5
ADCK1	10	9	8	HDDC2	6	6	6	RTCB	11	1	7
ADX	4	4	4	HEM2	8	9	43	RWDD1	7	7	7
ALG11	4	3	3	IF5	9	8	24	S35B2	3	2	2
AP2M1*	10	10	7	IMP4	9	1	33	SF3A2	6	7	7
ARP2	8	8	8	ISCU	5	5	5	SF3A3*	16	16	40
ASNA*	7	7	5	KAD2*	5	5	4	SIAH1	8	5	6
ATTY	16	11	9	KIF17	18	10	14	SIR1	11	7	6
BCS1	4	4	4	LIAS	11	1	7	SLBP	8	2	7
BRAP	15	2	14	MAEA	9	9	9	SLX1	5	4	4
BUB3*	6	6	19	MAF1*	6	6	4	SMUG1*	3	3	2
BYST	9	10	10	MCRS1	12	10	12	SNF8	8	1	7
CALR	9	10	46	MDHC	8	1	6	SNP29	3	1	32
CDC16*	16	16	17	MED18	6	6	6	SNX12	5	4	4
CDC27	20	7	11	MGAT1	11	9	8	SODM	5	4	5
CDC5L	16	1	15	MICU1	9	10	10	SRP72*	17	17	16
CDIPT	6	6	6	MTHFS	3	1	3	SSRB	6	5	4
CDO1*	4	4	3	MTMR9	9	13	13	STON2	3	2	2
CK5P3	15	13	51	MUL1*	3	3	5	SUCB2	11	1	8
CLPT1	11	14	10	NARFL	10	9	10	SYAP1	10	8	9
CNO10	16	13	14	NDUA6	3	3	3	TAD2B	9	13	14
COG4	21	21	21	NDUA8	4	3	3	TCPD*	14	14	11
COMD4	11	7	7	NDUV2*	9	9	7	TCPH*	12	12	7
COQ5	7	10	7	NFU1*	8	8	33	TCPQ*	16	16	10
CP072*	3	3	5	NIT2	9	9	9	TF2H4*	14	14	23
CSTF3*	20	20	19	NOM1	7	9	13	THIL	13	11	9
DBR1	9	7	7	NOP58	15	13	10	THIM	11	10	10
DCPS	4	1	4	NSF1C	10	1	8	THOC7	8	8	8
DCTN3	7	8	6	ODBA	10	1	8	TIM10	2	2	2
DDX27	19	11	13	PAR16	6	1	6	TIPRL	6	7	7
DDX52	9	15	11	PIGO	5	5	5	TM2D1	5	6	6
DGKE*	9	9	7	PIMT*	5	5	6	TMCO1	7	7	7
DHX37	30	23	26	PK3C3	27	22	21	TRM61	4	4	4
DIC	10	8	9	PSB3	6	6	6	TTI1	21	25	26
DJB11	9	9	9	PSB4	7	1	5	UB2J2*	7	7	31
DNAI1	19	17	20	PSMD7	7	6	5	UBA5	10	12	11
DUS4L	8	8	8	RAB6A	7	8	7	UBE2C	5	4	5
EFGM	27	18	18	RCL1	9	9	9	UBE2H	7	7	7
ETFB	6	2	10	RENT2	34	36	24	UBP7*	33	33	25
EXOS1	10	7	42	REV1	19	18	13	UFD1	13	11	12
EXOS5	2	1	1	RFC2	9	9	9	USO1	21	16	15
FBX11	19	17	14	RFC4	10	11	11	UTP11	8	8	8
FNTB	17	13	1	RL13A*	7	7	6	VPS18*	29	29	28
FUND1	5	4	5	RL17	6	2	3	VPS29*	4	4	11
GALK2	8	9	9	RL8*	6	6	5	VPS45	14	13	11
GANP	30	6	21	RL9	4	4	4	WBP4*	9	9	10
GATD1	2	3	5	RPAB2	5	1	3	WBS22	9	11	11
GID8	5	4	4	RPAB3	4	3	36	WDR82	9	9	9
GOSR1	9	8	7	RPIA	8	7	7	ZDH16	9	7	7
GPN2*	3	3	5	RPN1*	10	10	8	ZN598	6	4	4

Gene names are based on the human orthologs according to UniProt entry name without "_HUMAN" at the end. Three-letter code: lan, brachiopod (*Lingula anatina*); lgi, sea snail (*Lottia gigantea*); cte, polychaete (*Capitella teleta*). Genes labeled with asterisks (*) indicate the same intron number shared between lan and lgi (grey box) but not lan and cte.

Supplementary Table 11 | Number of genes with transcription factor-related domains in selected bilaterians

Pfam domain name	Pfam ID	Function	hsa	bfl	lan	lgi	cgi	pfu	cte	hro	tca	dpu
ARID	PF01388	ARID/BRIGHT DNA binding domain	15	4	11	6	7	7	7	10	8	6
Basic	PF01586	Myogenic Basic domain	4	4	1	1	1	0	1	1	1	0
bZIP_1	PF00170	bZIP transcription factor	45	37	37	36	40	34	31	29	21	26
bZIP_Maf	PF03131	bZIP Maf transcription factor	34	14	15	13	15	13	12	14	9	14
CUT	PF02376	CUT domain	7	3	4	4	3	3	3	11	2	3
DM	PF00751	DM DNA binding domain	7	9	5	4	3	2	5	3	3	5
Ets	PF00178	Ets-domain	28	13	16	10	16	19	13	22	9	9
Fork_head	PF00250	Fork head domain	50	31	27	31	26	31	47	31	19	17
GATA‡	PF00320	GATA zinc finger	20	7	9	6	6	6	16	15	7	5
GCM	PF03615	GCM motif protein	2	2	1	1	1	0	1	2	1	1
Hairy_orange	PF07527	Hairy Orange	12	12	15	12	10	9	14	5	8	7
HLH	PF00010	Helix-loop-helix DNA-binding domain	108	78	78	76	74	58	84	70	51	48
HMG_box	PF00505	HMG (high mobility group) box	56	39	43	29	29	29	25	66	31	33
Homeobox‡	PF00046	Homeobox domain	244	127	129	140	117	116	182	242	97	114
Homeobox_KN‡	PF05920	Homeobox KN domain	66	25	34	37	31	32	54	88	22	26
Hormone_recep	PF00104	Ligand-binding domain of nuclear hormone receptor	48	28	45	33	41	41	39	34	19	22
Neuro_bHLH	PF12533	Neuronal helix-loop-helix transcription factor	4	1	2	1	1	1	2	1	1	0
OAR†	PF03826	OAR domain	15	12	10	7	10	10	5	4	5	0
P53	PF00870	P53 DNA-binding domain	3	4	1	1	1	1	1	2	2	1
PAX	PF00292	'Paired box' domain	9	5	8	8	9	12	8	10	20	17
Pou	PF00157	Pou domain - N-terminal to homeobox domain	16	6	3	4	4	3	6	11	4	4
HPD	PF05044	Homeo-prospero domain	2	0	3	1	1	1	1	3	1	1
RHD	PF00554	Rel homology domain (RHD)	10	2	3	4	4	3	3	4	4	4
Runt	PF00853	Runt domain	3	1	1	1	1	1	2	2	4	4
SCAN	PF02023	SCAN domain	60	0	5	2	1	1	1	1	0	0
SRF-TF	PF00319	SRF-type transcription factor (DNA-binding and dimerisation domain)	5	3	2	3	5	4	2	6	2	2
T-box	PF00907	T-box	17	11	7	12	17	7	8	18	8	7
TF_AP-2	PF03299	Transcription factor AP-2	5	1	2	1	3	3	2	2	1	1
TF_Otx†*	PF03529	Otx1 transcription factor	3	0	1	0	1	1	0	0	0	0
zf-C2H2	PF00096	Zinc finger, C2H2 type	708	986	237	321	231	277	312	230	245	153
zf-C2HC	PF01530	Zinc finger, C2HC type	6	2	5	3	5	4	5	3	4	3
zf-C4	PF00105	Zinc finger, C4 type (two domains)	46	29	46	36	42	42	38	50	22	28

Domains expanded in *Lingula* and molluscs but not in annelids are labeled with daggers (†). Domains expanded in annelids but not in *Lingula* and mollsucs are labeled with double daggers (‡). Domain lost in annelids is labeled with astertisk (*). The major phyla are separated by vertical dashed lines. The numbers of *Lingula* genes are highlighted in grey. Three-letter code: hsa, human (*Homo sapiens*); bfl, amphioxus (*Branchiostoma floridae*); lan, brachiopod (*Lingula anatina*); lgi, sea snail (*Lottia gigantea*); cgi, Pacific oyster (*Crassostrea gigas*); pfu, pearl oyster (*Pinctada fucata*); cte, polychaete (*Capitella teleta*); hro, leech (*Helobdella robusta*); tca, beetle (*Tribolium castaneum*); dpu, water flea (*Daphnia pulex*).

Supplementary Table 12 | Number of genes with signaling pathway-related domains in selected bilaterians

Pfam domain name	Pfam ID	Function	hsa	bfl	lan	lgi	cgi	pfu	cte	hro	tca	dpu
CHRD†*	PF07452	CHRD domain	1	1	1	1	1	0	0	0	1	2
Dishevelled	PF02377	Dishevelled specific domain	5	0	2	1	1	1	1	0	1	0
DIX	PF00778	DIX domain	7	3	4	4	3	3	3	2	2	3
DSL	PF01414	Delta serrate ligand	4	2	4	5	11	17	8	3	4	2
EGF†	PF00008	EGF-like domain	125	527	263	103	222	176	214	99	39	49
FGF†	PF00167	Fibroblast growth factor	27	10	4	3	2	2	2	2	6	3
Focal_AT	PF03623	Focal adhesion targeting region	2	1	1	1	1	1	1	1	1	34
Frizzled	PF01534	Frizzled/Smoothened family membrane region	12	5	6	5	4	6	5	5	5	5
G-alpha†	PF00503	G-protein alpha subunit	48	29	44	37	36	27	34	35	27	30
G-gamma†	PF00631	GGL domain	16	1	7	4	5	5	6	6	4	4
HH_signal†	PF01085	Hedgehog amino-terminal signalling domain	3	3	5	3	3	3	1	1	2	1
MCPsignal	PF00015	Methyl-accepting chemotaxis protein (MCP) signalling domain	0	9	0	7	5	12	1	0	1	1
Notch†	PF00066	LNR domain	7	3	7	2	2	4	6	3	2	2
NPH3	PF03000	NPH3 family	0	0	0	0	1	1	1	0	0	0
PDGF	PF00341	PDGF/VEGF domain	9	2	2	2	2	1	2	1	2	8
Phe_ZIP†*	PF08916	Phenylalanine zipper	3	0	1	0	1	1	0	0	1	0
PTN_MK_C	PF01091	PTN/MK heparin-binding protein family, C-terminal domain	2	1	1	0	1	3	1	0	1	1
PTN_MK_N	PF05196	PTN/MK heparin-binding protein family, N-terminal domain	2	1	0	1	0	0	0	1	0	0
Rabaptin	PF03528	Rabaptin	2	2	0	1	1	0	1	2	0	0
RGS†	PF00615	Regulator of G protein signaling domain	36	14	23	14	16	16	17	17	11	17
STAT_alpha	PF01017	STAT protein, all-alpha domain	7	2	2	1	1	1	5	1	1	1
STAT_bind	PF02864	STAT protein, DNA binding domain	7	4	3	2	1	4	6	3	1	1
STAT_int	PF02865	STAT protein, protein interaction domain	7	3	2	1	2	1	6	2	1	1
TGF_beta†	PF00019	Transforming growth factor beta like domain	37	20	15	11	12	10	14	8	8	9
TGFb_propeptide†	PF00688	TGF-beta propeptide	28	15	14	10	7	10	10	5	10	7
wnt	PF00110	wnt family	19	15	17	12	12	11	16	18	9	13

Domains expanded (with highest number) in *Lingula* compared to other lophotrochozoans are labeled with daggers (†). Domains lost in annelids are labeled with astertisks (*). The major phyla are separated by vertical dashed lines. The numbers of *Lingula* genes are highlighted in grey. Three-letter code: hsa, human (*Homo sapiens*); bfl, amphioxus (*Branchiostoma floridae*); lan, brachiopod (*Lingula anatina*); lgi, sea snail (*Lottia gigantea*); cgi, Pacific oyster (*Crassostrea gigas*); pfu, pearl oyster (*Pinctada fucata*); cte, polychaete (*Capitella teleta*); hro, leech (*Helobdella robusta*); tca, beetle (*Tribolium castaneum*); dpu, water flea (*Daphnia pulex*).

Supplementary Table 13 | The 20 most abundant domains in *Lingula* compared with selected bilaterians

Pfam domain name	Pfam ID	Function	hsa	bfl	lan	lgi	cgi	pfu	cte	hro	tca	dpu
Pkinase†	PF00069	Protein kinase domain	482	554	576	320	360	381	377	473	222	403
Pkinase_Tyr†	PF07714	Protein tyrosine kinase	478	554	553	316	353	372	363	461	218	394
7tm_1	PF00001	7 transmembrane receptor (rhodopsin family)	722	571	504	307	412	521	1025	224	82	179
Ank_2	PF12796	Ankyrin repeats (3 copies)	259	244	428	201	338	370	432	166	140	317
Ank†	PF00023	Ankyrin repeat	256	238	407	190	324	349	421	165	138	287
Ank_5†	PF13857	Ankyrin repeats (many copies)	256	232	404	189	324	338	411	155	135	274
Ank_4†	PF13637	Ankyrin repeats (many copies)	256	229	398	188	328	340	413	157	137	281
Ank_3†	PF13606	Ankyrin repeat	247	225	388	185	320	329	397	156	136	262
MFS_1†	PF07690	Major Facilitator Superfamily	122	284	380	228	210	317	244	122	200	140
WD40†	PF00400	WD domain, G-beta repeat	261	255	362	227	236	245	244	200	178	239
LRR_4	PF12799	Leucine Rich repeats (2 copies)	285	1006	356	171	210	282	466	133	185	128
Lectin_C	PF00059	Lectin C-type domain	84	640	329	129	260	336	209	78	14	51
LRR_8	PF13855	Leucine rich repeat	244	1003	314	157	191	258	441	120	177	120
EF-hand_7†	PF13499	EF-hand domain pair	182	249	297	193	225	274	182	143	83	80
EF-hand_1†	PF00036	EF hand	184	247	296	204	227	278	182	144	82	80
LRR_1	PF00560	Leucine Rich Repeat	231	842	283	147	164	231	386	105	154	91
EF-hand_6†	PF13405	EF-hand domain	164	238	279	197	219	268	168	141	76	77
EGF†	PF00008	EGF-like domain	125	527	263	103	222	176	214	99	39	49
Miro†	PF08477	Miro-like protein	205	223	253	162	191	170	167	139	133	118
EGF_CA†	PF07645	Calcium-binding EGF domain	92	482	251	83	136	110	249	82	29	42

Highest expanded domains in *Lingula* compared to other lophotrochozoans are labeled with daggers (†). The major phyla are separated by vertical dashed lines. The numbers of *Lingula* genes are highlighted in grey. Three-letter code: hsa, human (*Homo sapiens*); bfl, amphioxus (*Branchiostoma floridae*); lan, brachiopod (*Lingula anatina*); lgi, sea snail (*Lottia gigantea*); cgi, Pacific oyster (*Crassostrea gigas*); pfu, pearl oyster (*Pinctada fucata*); cte, polychaete (*Capitella teleta*); hro, leech (*Helobdella robusta*); tca, beetle (*Tribolium castaneum*); dpu, water flea (*Daphnia pulex*).

Supplementary Table 14 | The 20 most expanded gene families in *Lingula*

Entry[a]	Entry name[b]	Protein name	Function	Copy #	P-value[c]	Highly expressed[d]
Q4P9K9	CHS8_USTMA*	Chitin synthase 8	Chitin synthesis	31	2.E-06	M,L,G,D
Q7LGC8	CHST3_HUMAN*	Carbohydrate sulfotransferase 3	Glycosaminoglycan (GAG; chondroitin sulfate, CS) biosynthesis	30	0.E+00	E,M
Q8N6F8	WBS27_HUMAN	Williams-Beuren syndrome chromosomal region 27 protein	Unknown	19	0.E+00	E,G,D
Q9BYK8	HELZ2_HUMAN	Helicase with zinc finger domain 2	Ttranscriptional coactivator for a number of nuclear receptors	17	4.E-04	L,G,D
Q8WUJ3	CEMIP_HUMAN*	Cell migration-inducing and hyaluronan-binding protein	Mediating depolymerization of GAG (hyaluronic acid, HA)	17	0.E+00	M
O60449	LY75_HUMAN	Lymphocyte antigen 75	Endocytic receptor, capturing antigens from the extracellular space	16	0.E+00	D
Q96NT5	PCFT_HUMAN	Proton-coupled folate transporter (G21)	Mediating heme uptake from the gut lumen into duodenal epithelial cells	16	4.E-06	D
P02751	FINC_HUMAN*	Fibronectin (FN)	Involving in cell adhesion and cell-mediated matrix assembly process	16	0.E+00	M,L,G,D
P23415	GLRA1_HUMAN	Glycine receptor subunit alpha-1	Neurotransmitter-gated ion channel	15	9.E-05	L
Q99102	MUC4_HUMAN*	Mucin-4	Altering cellular behavior through cell-extracellular matrix interactions	15	1.E-06	M,L
P15428	PGDH_HUMAN	15-hydroxyprostaglandin dehydrogenase	Prostaglandin inactivation	15	1.E-06	D
Q99489	OXDD_HUMAN	D-aspartate oxidase	Catalyzing the oxidative deamination of D-aspartate	15	0.E+00	M,L,G,D,P
Q6UW02	CP20A_HUMAN	Cytochrome P450 20A1	Monooxygenase with unkown function	14	0.E+00	M,D,P
Q96A11	G3ST3_HUMAN	Galactose-3-O-sulfotransferase 3	Proteoglycan biosynthesis	13	2.E-05	P
Q86WV6	STING_HUMAN	Stimulator of interferon genes protein	Facilitator of innate immune signaling	13	1.E-06	M,L,G,D
P20061	TCO1_HUMAN	Transcobalamin-1	Protecting vitamin B12 from the acidic environment of the stomach	13	0.E+00	M,L,P
P04054	PA21B_HUMAN	Phospholipase A2	Catalyzing phosphatidylcholine (PC)	12	4.E-06	D
P21589	5NTD_HUMAN	5'-nucleotidase	Hydrolyzing extracellular nucleotides into membrane permeable nucleosides	11	1.E-05	E
Q8NBI5	S43A3_HUMAN	Solute carrier family 43 member 3	Putative transporter with unkown function	11	1.E-05	M
Q5TF39	NAGT1_HUMAN	Sodium-dependent glucose transporter 1	Sodium-dependent glucose transporter	11	0.E+00	G,D

[a]UniProt entry ID. [b]Genes that are possibly related to shell formation are labeled with asterisks (*). [c]Significantly expanded gene families are tested by P-value calculated from 15 selected metazoan genomes with the Viterbi method using CAFE. [d]Abbreviations: E, embryos; M, mantle, L, lophophore; G, gut; D, digestive cecum; P, pedicle.

Supplementary Table 15 | Chitin synthase genes in *Lingula*

Gene ID[a]	Blastp 31	OrthoMCL 25	KEGG 17	Pfam domain(s)	Best hit to UniProt	Expression[b]
05204	+	+		Chitin_synth_2	CHS1_CRYNH	ND
05483	+	+		Chitin_synth_2	CHS8_USTMA	A only
05484	+	+	+	Chitin_synth_2	CHS2_USTMA	A only
06947		+		No-hit	No-hit	ND
07365	+	+	+	Chitin_synth_2	CHS2_PARBR	ND
07368	+	+	+	Chitin_synth_2	CHS3_EXODE	A only
07383	+	+	+	Chitin_synth_2	CHSC_ASPFU	ND
07385	+	+	+	Chitin_synth_2	CHS2_PARBR	ND
08249	+	+	+	Chitin_synth_2	CHS1_NEUCR	E&A
10157	+		+	Chitin_synth_2	CHS1_USTMA	E&A
10838	+	+	+	Chitin_synth_2	CHS8_USTMA	ND
13561*	+	+	+	Myosin_head, Chitin_synth_2	MYO3B_HUMAN	E&A
14064	+	+		Chitin_synth_2	CHS8_USTMA	A only
14065		+		No-hit	No-hit	ND
14334†	+	+	+	Chitin_synth_2, SAM_2, SAM_1	CHS1_CRYNH	A only
16731†	+	+	+	Chitin_synth_2, SAM_2, SAM_1	CHS6_USTMA	A only
16893	+			Chitin_synth_2	NODC_RHIGA	E only
18179	+	+	+	Chitin_synth_2	CHS1_CRYNH	ND
18723†	+	+	+	zf-TAZ, Chitin_synth_2, SAM_2, SAM_1	CBP_RAT	E&A
19590	+			Chitin_synth_2	CHS2_NEUCR	E only
21358†	+	+		Chitin_synth_2, SAM_2, SAM_1	CHS2_USTMA	ND
24021		+		No-hit	No-hit	ND
24329	+		+	Chitin_synth_2	CHS8_USTMA	ND
26406	+			Chitin_synth_2	NODC_RHIGA	ND
29127	+	+	+	Chitin_synth_2	CHS1_YEAST	ND
29294	+			Chitin_synth_2	CHS2_NEUCR	ND
29711	+			(RVT_1, Peptidase_A17)x2, rve, Chitin_synth_2	CHS6_USTMA	ND
30735	+			Chitin_synth_2	CHS8_USTMA	A only
31332	+		+	Chitin_synth_2	CHS6_USTMA	A only
31400	+			Chitin_synth_2	CHS6_USTMA	ND
31417	+			Chitin_synth_2	NODC_RHIGA	ND
31493	+	+		Chitin_synth_2	CHS8_USTMA	ND
32229		+		SUV3_C	No-hit	ND
32630†	+	+		Chitin_synth_2, SAM_1	CHS1_CRYNH	ND
32837	+		+	(Chitin_synth_2)x2	CHS6_USTMA	ND
32872		+		Myosin_head	MYO3B_HUMAN	ND
33112		+		No-hit	No-hit	ND

[a]Chitin synthase (CHS) with a myosin motor head is labeled with an asterisk (*). CHSs with SAM domains are labeled with daggers (†) [b]ND, not detected; A, adult tissues; E, embryonic stages. +, detected in given analyses.

Supplementary Table 16 | Transposable elements in the *Lingula* genome

Class of transposons	Total length	Percentage in the genome (%)
DNA transposons	12,192,180	2.865
TcMar	9,973,854	2.344
Zator	854,703	0.201
Academ	493,160	0.116
PIF	375,492	0.088
Maverick	184,373	0.043
Ginger	100,154	0.024
hAT	95,432	0.022
Kolobok	38,345	0.009
Sola	37,190	0.009
CMC	19,181	0.005
Helitron	16,472	0.004
MuLE	3,824	0.001
Retrotransposons	9,857,666	2.317
LTRs (Long terminal repeats)		
Gypsy	882,079	0.207
DIRS	60,227	0.014
Ngaro	24,446	0.006
Pao	23,516	0.006
LINEs (Long interspersed elements)		
RTE	3,113,168	0.732
L2	1,824,206	0.429
Penelope	1,443,806	0.339
Rex	1,372,563	0.323
L1	828,288	0.195
CR1	265,476	0.062
Dong	10,292	0.002
Proto2	6,139	0.001
I	1,737	0.000
Jockey	1,723	0.000
Simple repeat	8,986,631	2.112
Unspecified	60,818,593	14.294

Supplementary Table 17 | Comparison of mineral composition in *Lingula*, molluscs, and vertebrates

	Lingula	Molluscs	Vertebrates
Chemical composition	Calcium phosphate	Calcium carbonate	Calcium phosphate
Mineral	Fluorapatite	Calcite, Aragonite	Hydroxyapatite
Formula	$Ca_{10}(PO_4)_6F_2$	$CaCO_3$	$Ca_{10}(PO_4)_6(OH)_2$
Fibrillar collagen	Yes	No	Yes
Chitin	Yes	Yes	No[a]

[a]No chitin in the bone matrix has been reported.

Supplementary Table 18 | Functional annotation of mantle-specific genes based on gene GO enrichment terms

Annotation Cluster	Enrichment Score	Database	Term	Genes involved in the term	%	*P*-value	Fold Enrichment
Extracellular glycoprotein	19.6	UP_SEQ_FEATURE	glycosylation site:N-linked (GlcNAc...)	363	29.88	2.06E-31	1.74
		SP_PIR_KEYWORDS	glycoprotein	374	30.78	5.30E-30	1.69
		UP_SEQ_FEATURE	disulfide bond	214	17.61	1.53E-20	1.84
Membrane glycoprotein	17.0	UP_SEQ_FEATURE	glycosylation site:N-linked (GlcNAc...)	363	29.88	2.06E-31	1.74
		SP_PIR_KEYWORDS	glycoprotein	374	30.78	5.30E-30	1.69
		UP_SEQ_FEATURE	topological domain:Extracellular	205	16.87	3.13E-18	1.79
G protein receptor	12.2	SP_PIR_KEYWORDS	receptor	121	9.96	1.54E-17	2.21
		GOTERM_BP_FAT	G-protein coupled receptor protein signaling pathway	68	5.60	2.96E-15	2.79
		INTERPRO	IPR017452:GPCR, rhodopsin-like superfamily	40	3.29	5.22E-15	4.07
Extracelular and plasma membrane	10.8	UP_SEQ_FEATURE	Extracellular	205	16.87	3.13E-18	1.79
		GOTERM_CC_FAT	plasma membrane	241	19.84	1.61E-08	1.37
		SP_PIR_KEYWORDS	cell membrane	141	11.60	8.20E-08	1.55
EGF	5.8	INTERPRO	IPR006210:EGF-like	53	4.36	6.73E-09	2.33
		SMART	SM00181:EGF	53	4.36	1.49E-08	2.25
		INTERPRO	IPR006209:EGF	45	3.70	2.30E-08	2.45
Sulfotransferase	5.5	GOTERM_MF_FAT	transferase activity, transferring sulfur-containing groups	24	1.98	3.07E-07	3.27
		GOTERM_MF_FAT	sulfotransferase activity	21	1.73	9.82E-07	3.40
		INTERPRO	IPR018011:Carbohydrate sulfotransferase-related	9	0.74	3.68E-06	7.40
Cell adhesion	5.5	GOTERM_BP_FAT	cell adhesion	71	5.84	5.41E-07	1.82
		GOTERM_BP_FAT	biological adhesion	71	5.84	5.41E-07	1.82
		SP_PIR_KEYWORDS	cell adhesion	41	3.37	1.49E-04	1.85
Extracellular matrix	5.3	SP_PIR_KEYWORDS	extracellular matrix	38	3.13	2.04E-06	2.29
		GOTERM_CC_FAT	extracellular matrix	48	3.95	3.75E-06	2.00
		GOTERM_CC_FAT	extracellular region part	75	6.17	4.06E-06	1.69
Neuropeptide binding	5.2	GOTERM_MF_FAT	peptide receptor activity	18	1.48	6.98E-07	3.94
		GOTERM_MF_FAT	peptide receptor activity, G-protein coupled	18	1.48	6.98E-07	3.94
		GOTERM_MF_FAT	neuropeptide receptor activity	14	1.15	2.69E-06	4.54
Sushi	4.0	INTERPRO	IPR000436:Sushi/SCR/CCP	16	1.32	2.03E-05	3.51
		SMART	SM00032:CCP	16	1.32	2.95E-05	3.38
		INTERPRO	IPR016060:Complement control module	16	1.32	8.15E-05	3.16
Fibronectin	3.8	UP_SEQ_FEATURE	domain:Fibronectin type-III 7	15	1.23	5.64E-05	3.43
		UP_SEQ_FEATURE	domain:Fibronectin type-III 1	23	1.89	1.00E-04	2.47
		UP_SEQ_FEATURE	domain:Fibronectin type-III 2	23	1.89	1.00E-04	2.47
Pentaxin	3.7	INTERPRO	IPR001759:Pentaxin	8	0.66	1.06E-04	6.07
		SMART	SM00159:PTX	8	0.66	1.32E-04	5.86
		UP_SEQ_FEATURE	domain:Pentaxin	7	0.58	4.54E-04	6.01
Chondroitin sulfate metabolic process	3.7	GOTERM_BP_FAT	aminoglycan metabolic process	20	1.65	1.34E-05	3.04
		GOTERM_BP_FAT	glycosaminoglycan metabolic process	14	1.15	1.24E-04	3.38
		GOTERM_BP_FAT	chondroitin sulfate metabolic process	8	0.66	5.26E-04	4.95

This analysis was conducted with DAVID. The top 3 terms are listed for each annotation cluster.

Supplementary Table 19 | Genes highly expressed (FPKM>100) in mantle tissue

Gene ID	Transcript ID	Best hit to UniProt	Protein name	GO cellular component	L	MT	LP	GT	DC	PC
13995	comp130956_c0	COKA1_MOUSE	Collagen alpha-1(XX) chain	extracellular region	0	2139	76	2	1	0
10202	comp38020_c0	R7V0B0_CAPTE	Uncharacterized protein	NA	3	1476	0	0	0	1
18117	comp144785_c0	C3YI43_BRAFL	Putative uncharacterized protein	NA	10	594	50	6	7	31
11761	comp135679_c1	ZAN_RABIT	Zonadhesin	plasma membrane	0	416	9	0	0	0
03146	comp131601_c0	CO6A4_MOUSE	Collagen alpha-4(VI) chain	extracellular region	0	410	0	0	0	0
11763	comp106172_c0	ZAN_RABIT	Zonadhesin	plasma membrane	0	381	0	0	0	0
30541	comp151635_c1	PAL2_CICAR	Phenylalanine ammonia-lyase 2	cytoplasm	3	376	43	8	12	12
23590	comp153570_c3	HSP71_ANOAL	Heat shock protein 70 A1	NA	15	345	15	11	18	15
01960	comp133336_c0	FCGBP_HUMAN	IgGFc-binding protein	cytoplasm	0	304	0	0	0	0
03108	comp142561_c4	K1RDK5_CRAGI	Uncharacterized protein	NA	3	298	43	4	2	2
27258	comp102482_c0	ABFB_EMENI	Alpha-L-arabinofuranosidase B	extracellular region	0	280	49	1	1	0
00827	comp134377_c0	B7PYM0_IXOSC	Putative uncharacterized protein	NA	1	273	1	1	1	1
16769	comp129574_c0	E0UDJ8_CYAP2	Uncharacterized protein	NA	2	272	48	1	0	0
26410	comp133581_c1	CALM4_MOUSE†	Calmodulin-4	extracellular vesicular exosome	19	261	22	2	4	20
27616	comp140975_c1	CNN3_HUMAN†	Calponin-3	cytoplasm	24	254	26	5	7	2469
09659	comp108623_c1	YLK2_CAEEL†	EGF-like domain-containing protein D1044.2	integral component of membrane	1	229	0	0	0	2
13590	comp134106_c0	FCGBP_HUMAN	IgGFc-binding protein	cytoplasm	0	206	11	0	0	0
03256	comp148732_c1	FCL_CRIGR	GDP-L-fucose synthase	NA	32	197	37	10	5	17
27773	comp121945_c1	MSHA_CORA7	D-inositol 3-phosphate glycosyltransferase	NA	41	180	5	4	2	6
26029	comp129548_c0	MUC5B_CHICK	Mucin-5B	extracellular region	0	135	0	0	0	0
08940	comp133810_c0	UROM_CANFA†	Uromodulin	extracellular region	0	123	18	0	0	0
02131	comp78413_c0	C3YUZ7_BRAFL†	Putative uncharacterized protein	NA	0	122	0	0	0	0
29625	comp78997_c0	VWF_RAT	von Willebrand factor	extracellular region	0	120	1	0	1	13
30006	comp134304_c1	CHSTB_RAT	Carbohydrate sulfotransferase 11	Golgi membrane	0	112	16	0	0	0

Expression level is shown as Fragments Per Kilobase of transcript per Million mapped reads (FPKM). Genes with GO molecular function in calcium ion binding are marked with daggers (†). Mantle tissue is highlighted in grey where gene expression may imply their roles in shell formation. Embryonic stage and adult tissues are separated by a vertical dashed line. L, larva; MT, mantle; LP, lophophore; GT, gut; DC, digestive cecum; PC, pedicle.

Supplementary Table 20 | Numbers of homologs associated with biomineralization found in selected bilaterians

	Human	Shark	*Lingula*	Pearl oyster	Pacific oyster	Sea snail
Vertebrate bone formation						
BMP signaling	25	19	15	12	14	14
FGF signaling	17	10	7	7	7	8
Hedgehog signaling	20	13	14	13	13	14
Transcription factors	18	15	14	13	14	14
Differentiation	49	41	37	33	32	35
Osteoclast specific	10	8	6	6	7	6
Proteoglycans	16	9	3	2	5	4
Haparins	8	4	5	2	2	2
SPARCs & SCPPs	12	2	1	1	1	1
Mollusc shell formation-related proteins	40	37	45	77	54	48
Spider silk proteins	0	0	0	0	0	0
Lingula shell matrix proteins	26	26	65	30	31	32

BMP, bone morphogenetic protein; FGF, fibroblast growth factor; SPARCs, secreted proteins acidic and rich in cysteine; SCPPs, secreted calcium-binding phosphoproteins. Human, *Homo sapiens*; shark, *Callorhinchus milii*; pearl oyster, *Pinctada fucata*; Pacific oyster *Crassostrea gigas*; sea snail, *Lottia gigantea*. Categories are marked in italic.

Supplementary Table 21 | Expression profiles of vertebrate bone formation-related genes in *Lingula*: signaling components and transcription factors

Category[a]	Gene name[b]	Gene ID	Transcript ID	B	MG	L	MT	LP	GT	DC	PC
BMP	ACVR1	23724	comp151002_c0	53	55	26	13	17	9	10	10
BMP	AVR2B	11314	comp156042_c0	37	52	41	24	32	30	18	17
BMP	BMP3	04706	comp149594_c0	13	30	19	0	0	1	3	0
BMP	BMP4	09932	comp125124_c3	16	22	18	4	5	2	4	2
BMP	BMP7	24996	comp154511_c0	51	33	16	8	5	2	10	4
BMP	BMPR2	02574	comp151731_c0	2	4	1	3	3	5	2	9
BMP	BMR1B	08775	comp145623_c0	13	17	13	10	13	21	12	18
BMP	CHRD	24246	comp155946_c0	28	42	6	6	2	1	1	2
BMP	FST	05233	comp129356_c0	0	0	5	7	9	13	4	13
BMP	GREM1	09906	comp128018_c0	0	1	1	1	3	1	1	0
BMP	NOGG	17517	comp114181_c1	0	0	1	2	3	1	0	1
BMP	SMAD4	13458	comp153142_c0	26	24	35	12	14	13	10	12
BMP	SMAD5	06646	comp151818_c0	107	78	37	19	23	20	29	56
BMP	SMAD6	20055	comp140924_c1	6	10	8	4	4	4	4	1
BMP	SMUF2	18299	comp151039_c1	20	26	30	16	14	19	15	28
FGF	FGFR2	01550	comp144963_c0	12	22	55	27	21	35	26	16
FGF	MK01	13532	comp142866_c0	120	79	24	47	39	33	31	40
FGF	MK08	03899	comp139020_c1	3	5	15	9	9	10	7	14
FGF	RAC1	04280	comp146854_c0	39	54	137	57	58	47	62	48
FGF	RAF1	14169	comp151296_c0	11	9	7	6	7	6	9	10
FGF	RASH	14122	comp148465_c1	16	23	25	27	19	24	42	30
FGF	SPY2	16964	comp139589_c2	7	11	17	26	9	11	11	10
HH	CDON	08420	comp155051_c1	1	3	7	6	10	15	15	5
HH	DISP1	23228	comp138199_c0	3	3	3	4	3	8	2	4
HH	GAS1	16367	comp141019_c1	4	2	3	8	7	1	2	5
HH	GLI3	02580	comp156832_c0	1	1	11	9	5	0	1	4
HH	GPC3	27534	comp153443_c0	29	32	11	14	5	9	7	13
HH	HHAT	18977	comp152791_c0	7	5	4	2	3	1	1	6
HH	HHIP	27843	comp149084_c0	8	10	12	22	11	6	3	24
HH	IHH	09573	comp143638_c0	0	0	3	2	5	49	3	1
HH	KIF7	03227	comp154426_c1	11	12	9	8	10	13	6	5
HH	LBN	19623	comp154532_c0	1	1	3	7	46	11	3	1
HH	PTC1	10768	comp128742_c0	9	16	10	13	20	11	5	5
HH	SCUB1	17817	comp145409_c1	0	1	4	36	5	6	3	27
HH	SMO	10508	comp149123_c0	20	57	21	20	26	11	8	15
HH	SUFU	00133	comp132453_c0	30	23	27	12	20	18	12	11
TF	ATF4	3292	comp140740_c2	6	12	68	40	17	21	6	61
TF	FOS	00313	comp121592_c1	27	60	93	3910	1270	2342	741	42
TF	MITF	18700	comp146794_c0	9	11	28	135	75	75	88	71
TF	MSX2	21761	comp130312_c0	4	23	19	23	3	0	0	3
TF	NFAC1	03040	comp155839_c0	3	5	2	10	7	8	10	4
TF	NKX32	21763	comp140997_c0	1	0	0	0	1	1	1	15
TF	PDLI7	08773	comp155077_c1	26	60	24	8	9	8	6	15
TF	RUNX2	03722	comp128792_c0	0	0	75	1	1	0	3	0
TF	SOX6	14704	comp141690_c0	5	9	10	10	8	45	32	11
TF	SOX9	05515	comp147643_c1	10	8	39	45	21	31	19	167
TF	SP3	11447	comp151883_c0	5	6	3	5	6	4	3	8
TF	SP7	01702	comp147809_c1	27	70	20	9	1	0	0	14
TF	SPI1	10304	comp134554_c4	12	19	6	4	2	5	3	8
TF	TWST2	20986	comp147718_c0	1	17	4	5	11	1	0	6

[a]Components of bone morphogenetic protein (BMP) signaling, fibroblast growth factor (FGF) signaling, and hedgehog (HH) signaling; transcription factors (TF). [b]UniProt human ID. Expression level is shown as Fragments Per Kilobase of transcript per Million mapped reads (FPKM). Genes involved in different functions are separated by horizontal dashed lines. Embryonic stages and adult tissues are separated by a vertical dashed line. Mantle tissue is highlighted in grey. B, blastula, MG, mid-gastrula, L, larva; MT, mantle; LP, lophophore; GT, gut; DC, digestive cecum; PC, pedicle.

Supplementary Table 22 | Expression profiles of vertebrate bone formation-related genes in *Lingula*: differentiation and others

Category	Gene name[a]	Gene ID	Transcript ID	B	MG	L	MT	LP	GT	DC	PC
Differentiation	ANKH	26099	comp145937_c0	2	3	10	5	3	4	7	3
Differentiation	AT2B1	16621	comp154884_c0	60	50	20	54	34	33	28	189
Differentiation	ATS18	07895	comp155485_c1	2	2	6	3	4	3	3	2
Differentiation	BMP1	06336	comp139808_c0	75	18	22	36	44	8	6	156
Differentiation	CANT1	23237	comp150834_c1	9	6	9	3	4	5	4	5
Differentiation	CO1A2	19810	comp155159_c0	0	0	58	35	94	1	0	0
Differentiation	CO2A1	27162	comp138233_c1	0	0	6	126	16	6	23	970
Differentiation	CRTAP	01592	comp151767_c8	3	5	4	13	7	14	8	19
Differentiation	ENPP1	00127	comp151403_c2	1	0	3	2	0	1	7	1
Differentiation	ENTP5	15499	comp152740_c0	26	12	8	6	4	6	2	2
Differentiation	EXT1	17421	comp153432_c2	9	7	6	16	11	15	5	9
Differentiation	EXT2	05014	comp153984_c0	23	15	9	11	10	12	9	18
Differentiation	EXTL3	22793	comp155951_c1	13	9	9	5	4	4	4	7
Differentiation	FAM3C	09569	comp153859_c1	10	10	8	8	8	12	16	9
Differentiation	GALNS	09039	comp155163_c1	34	7	2	6	3	5	4	11
Differentiation	GALT3	23501	comp156631_c0	15	9	15	7	6	25	26	9
Differentiation	HS2ST	20680	comp150678_c0	44	23	12	21	15	20	17	11
Differentiation	HYAS2	08249	comp149935_c0	0	0	18	6	0	19	10	19
Differentiation	MATN1	30027	comp149858_c1	0	0	0	0	0	5	315	0
Differentiation	MMP1	25962	comp150297_c0	1	3	70	34	11	12	4	8
Differentiation	MMP13	26851	comp143111_c0	0	1	5	0	0	0	0	1
Differentiation	PGH2	21648	comp153995_c1	0	0	78	1	0	0	0	0
Differentiation	PHEX	25752	comp151433_c3	1	3	6	19	4	35	40	1
Differentiation	PHOP2	15146	comp141753_c0	2	3	1	1	2	2	1	1
Differentiation	PPBT	02796	comp146003_c1	0	0	2	0	0	109	75	0
Differentiation	RSPO3	04788	comp110257_c2	0	0	3	13	4	2	2	1
Differentiation	S35B2	19246	comp148430_c3	10	6	19	8	7	3	3	11
Differentiation	SOSD1	13588	comp152197_c1	4	8	7	1	3	2	3	4
Differentiation	SPTB2	15831	comp154657_c0	10	10	22	98	57	49	27	258
Differentiation	SUCO	12934	comp145086_c3	8	8	14	16	15	46	25	12
Differentiation	UXS1	20295	comp116798_c0	13	14	16	25	23	36	14	25
Osteoclast	EGR1	00915	comp123878_c0	1	2	3	2527	1231	1867	573	15
Osteoclast	OSTF1	19475	comp149203_c0	3	4	27	11	19	10	22	13
Osteoclast	TNF11	30098	comp133569_c2	0	0	1	60	58	138	195	1
Proteoglycans	FINC	31031	comp138749_c1	0	0	2	6	3	6	11	3
Proteoglycans	FINC	31031	comp148942_c2	0	0	1	11	11	22	47	1
Proteoglycans	FINC	31031	comp150132_c1	0	0	0	12	11	43	84	0
Proteoglycans	P3H1	01592	comp151767_c8	3	5	4	13	7	14	8	19
Proteoglycans	PODN	15316	comp136896_c1	0	0	0	18	40	5	1	0
Proteoglycans	PODN	15316	comp147278_c3	0	0	0	3	6	3	3	1
Proteoglycans	PODN	15316	comp156841_c0	1	3	18	1	0	0	0	0
Heparins	CSPG2	12529	comp134546_c6	0	0	0	0	0	34	13	0
Heparins	CSPG2	12529	comp136785_c0	0	0	0	0	0	1	149	0
Heparins	CSPG2	12529	comp143055_c1	0	0	0	0	0	307	363	0
Heparins	E7EX88	22242	comp135101_c0	4	2	6	329	294	41	29	221
Heparins	E7EX88	22242	comp136268_c3	1	0	0	0	492	11	0	0
Heparins	NCAN	05721	comp135115_c3	0	0	0	4	17	8	40	0
Heparins	NCAN	05721	comp141499_c0	0	0	0	4	16	1	1	0
Heparins	NCAN	05721	comp148773_c0	0	0	0	0	0	127	96	0
SPARC	SPRC	01638	comp124545_c2	0	0	22	70	86	9	27	215

[a]UniProt human ID. Expression level is shown as Fragments Per Kilobase of transcript per Million mapped reads (FPKM). Gene models with different transcripts isoforms are shown separately with different transcript IDs. Genes involved in different functions are separated by horizontal dashed lines. Embryonic stages and adult tissues are separated by a vertical dashed line. Mantle tissue is highlighted in grey. B, blastula, MG, mid-gastrula, L, larva; MT, mantle; LP, lophophore; GT, gut; DC, digestive cecum; PC, pedicle; SPARC, secreted protein acidic and rich in cysteine.

Supplementary Table 23 | Expression profiles of mollusc shell formation-related genes in *Lingula*: shared core sets in selected bilaterians

Gene name	Species	NCBI ID	*Lingula* gene ID	Transcript ID	L	MT	LP	GT	DC	PC
67kD laminin receptor precursor	*Pinctada fucata*	ABO10190	09282	comp141336_c1†	97	454	345	379	643	301
ACCBP 1	*Pinctada fucata*	ABF13208	01540	comp152364_c0	1	11	11	0	0	1
Alkaline phosphatase	*Pinctada fucata*	AAV69062	02796	comp146003_c1	2	0	0	109	75	0
BMP2/4	*Saccostrea kegaki*	BAG68618	09932	comp125124_c3	18	4	5	2	4	2
BMSP	*Mytilus galloprovincialis*	BAK86420	18155	comp138782_c3	1	2	324	14	12	1
BMSP	*Mytilus galloprovincialis*	BAK86420	18155	comp144291_c0	0	0	0	3	74	0
BMSP	*Mytilus galloprovincialis*	BAK86420	18155	comp149465_c0	2	0	9	202	143	0
BMPR2	*Crassostrea gigas*	CAD20574	02574	comp151731_c0	1	3	3	5	2	9
CA like	*Pinctada fucata*	BAJ52887	12996	comp141374_c0	0	1	0	9	0	1
Calcineurin A subunit	*Pinctada fucata*	ACI96106	05198	comp148420_c0†	53	57	62	89	56	139
Calcineurin B subunit	*Pinctada fucata*	ACI96107	09775	comp140873_c0†	40	18	27	15	13	61
Calcium/calmodulin-dependent serine protein kinase	*Lymnaea stagnalis*	AAO83853	31538	comp138049_c0	0	1	2	1	0	6
Calmodulin	*Hyriopsis schlegelii*	ACI22622	23066	comp131930_c0†	345	235	275	307	229	495
Calreticulin	*Pinctada fucata*	ABR68546	26826	comp132691_c0	263	104	158	62	138	482
Carbonic anhydrase precursor	*Tridacna gigas*	AAX16122	17981	comp144544_c2*	0	28	2	9	0	0
Engrailed	*Saccostrea kegaki*	BAG68617	29453	comp151141_c0†	64	0	0	0	0	0
Ferritin like protein	*Pinctada fucata*	AAQ12076	21091	comp140617_c2†	1469	2478	1033	2231	9595	1632
Hox4	*Gibbula varia*	ACX84672	10888	comp149466_c0†	16	0	0	1	0	0
IMSP-2	*Crassostrea gigas*	P86785	06306	comp141881_c5	3	11	15	4	1	10

(*Continued*)

Supplementary Table 23 Continued

Gene name	Species	NCBI ID	*Lingula* gene ID	Transcript ID	L	MT	LP	GT	DC	PC
L-type voltage-dependent calcium channel alpha-1 subunit isoform c	*Lymnaea stagnalis*	AAO83840	17989	comp146389_c0	1	0	0	0	0	0
L-type voltage-dependent calcium channel alpha-1 subunit isoform c	*Lymnaea stagnalis*	AAO83840	17989	comp151889_c1	2	1	0	1	0	5
L-type voltage-dependent calcium channel alpha-1 subunit isoform c	*Lymnaea stagnalis*	AAO83840	17989	comp156742_c0	21	17	7	11	1	35
L-type voltage-dependent calcium channel beta subunit	*Pinctada fucata*	ABL98211	04961	comp136043_c2	14	18	11	11	5	117
Neuronal calcium sensor-1	*Lymnaea stagnalis*	AAZ66779	03452	comp141341_c1*	2	5	1	0	0	3
Perlucin	*Haliotis laevigata*	P82596	25055	comp135101_c0†*	6	329	294	41	29	221
Perlucin	*Haliotis diversicolor*	ADD16957	02704	comp147268_c4†*	1	32	4	1	3	18
pfGbeta1	*Pinctada fucata*	Q5GIS3	01114	comp149934_c0	73	49	42	34	37	66
PFMG12	*Pinctada fucata*	AAZ22321	02554	comp132695_c2	1	2	0	0	0	27
PFMG12	*Pinctada fucata*	AAZ22321	02554	comp140078_c1	12	0	0	0	1	0
PFMG2	*Pinctada fucata*	AAZ76256	21098	comp137936_c1	14	5	2	0	0	38
PFMG2	*Pinctada fucata*	AAZ76256	21098	comp139732_c1†*	109	256	126	99	53	185
PFMG9	*Pinctada fucata*	AAZ22318	01110	comp150805_c2	0	0	0	1	10	0
PFMG9	*Pinctada fucata*	AAZ22318	01110	comp156486_c0	6	20	6	6	5	5
Plasma membrane calcium ATPase	*Pinctada fucata*	ABL63470	16621	comp154884_c0	20	54	34	33	28	189
Sarco/endoplasmic reticulum calcium ATPase isoform A	*Pinctada fucata*	ABS19815	21332	comp139209_c1	1	1	1	0	0	1
Serine threonine protein-kinase H1 homolog	*Pinctada fucata*	Q4KTY1	26560	comp152230_c1	6	4	4	4	3	4
TFG beta signaling pathway factor	*Pinctada fucata*	ABX57736	20056	comp150055_c2	8	9	14	12	11	12

The genes listed here are all shared by *Lingula*, sea snail, Pacific oyster, pearl oyster, and human. Expression level is shown as Fragments Per Kilobase of transcript per Million mapped reads (FPKM). Gene models with different transcript isoforms are shown separately with different transcript IDs. Transcripts that have the highest expression level at the larval stage during embryogenesis are labeled with dagger (†). Transcripts that are highly expressed in the mantle tissue are labeled with asterisks (*). Mantle tissue is highlighted in grey where expression profile may imply their roles in shell formation. Embryonic stage and adult tissues are separated by a vertical dashed line. L, larva; MT, mantle; LP, lophophore; GT, gut; DC, digestive cecum; PC, pedicle.

Supplementary Table 24 | Expression profiles of mollusc shell formation-related genes in *Lingula*: others

Gene name	Species	Shared by[a]	NCBI ID	*Lingula* gene ID	Transcript ID	L	MT	LP	GT	DC	PC
Calcium-dependent protein kinase	*Crassostrea gigas*	LOCP	AAU93878	26410	comp133581_c1†*	19	243	20	2	3	17
Chitin synthase	*Pinctada fucata*	LOCP	BAF73720	13561	comp142439_c1†	10	28	78	2	0	0
EP protein precursor	*Mytilus edulis*	LOPH	AAQ63463	00340	comp138794_c1	2	25	6	26	18	147
EP protein precursor	*Mytilus edulis*	LOPH	AAQ63463	00340	comp145542_c6†	15	5	4	3	16	4
Ependymin related protein 1	*Haliotis asinina*	LOCP	P86734	14790	comp128760_c2	1	0	1	163	601	0
IMSP-3	*Crassostrea gigas*	LOCP	P86786	04518	comp143931_c1	1	6	27	19	0	0
IMSP-6	*Crassostrea gigas*	LOCP	P86789	31214	comp140567_c0	3	1	1	6	16	1
Jacalin-related lectin PPL2-a	*Pteria penguin*	LPH	BAG80527	13721	comp132274_c0	0	0	3	0	0	2
Lectin	*Pteria penguin*	LCP	BAB03232	10095	comp147111_c0	0	6	9	0	0	0
Perlustrin	*Haliotis laevigata*	LOP	P82595	04676	comp125631_c0	0	0	0	0	0	19
PFMG4	*Pinctada fucata*	LCPH	AAZ76258	00347	comp133071_c0	0	0	1	2	1	0
PFMG8	*Pinctada fucata*	LP	AAZ76262	00479	comp149493_c0†	17	17	29	12	45	41
Pfty1	*Pinctada fucata*	LPH	BAF42771	10467	comp151045_c0	0	0	0	0	1	28
Putative uncharacterized protein F18	*Crassostrea nippona*	LOCP	BAG50305	31626	comp152218_c0†*	10	112	46	24	70	22
Tyrosinase	*Pinctada fucata*	LOCP	AAZ66340	09477	comp145394_c2†	257	3	0	0	0	0
Veliger mantle 1	*Haliotis asinina*	LOCP	ABD47938	10342	comp126542_c0†	19	0	0	9	6	0

[a]Abbreviations: L, *Lingula*; O, *Lottia* (sea snail); C, *Crassostrea* (Pacific oyster); P, *Pinctada* (pearl oyster); H, *Homo* (human). Expression level is shown as Fragments Per Kilobase of transcript per Million mapped reads (FPKM). Gene models with different transcript isoforms are shown separately with different transcript IDs. Transcripts that have the highest expression level at the larval stage during embryogenesis are labeled with daggers (†). Transcripts that are highly expressed in the mantle tissue are labeled with asterisks (*). Mantle tissue is highlighted in grey where gene expression may imply their roles in shell formation. Embryonic stage and adult tissues are separated by a vertical dashed line. L, larva; MT, mantle; LP, lophophore; GT, gut; DC, digestive cecum; PC, pedicle.

Supplementary Table 25 | Characterization of *Lingula* shell matrix proteins (SMPs) with detectable homologies to metazoan proteins

Gene ID[a]	Best hit to UniProt	Pfam domain(s)	Fraction[b]	Signal peptide	Unique peptide hit(s)	Length	MW (kDa)	pI	A (%)	G (%)	Acidic (%)[c]
00259	PRS42_MOUSE	Trypsin, HYR, FXa_inhibition, cEGF, Big_3_4	ASP	Yes	1	759	83	5.5	4	9	10
01003*	SVEP1_RAT	VWA, VWD, VWA_2, C8, GCC2_GCC3, CUB, Sushi, EGF, HYR, I-set, VWA_3, V-set, SEA, EGF_3, Ldl_recept_a, TIL, EGF_CA	AIP	Yes	1	8126	880	4.9	6	8	10
01574	C3Y3Y7_BRAFL	ND	AIP	Yes	1	644	71	5.7	6	8	10
01575	VDR_BOVIN	zf-C4, Hormone_recep	ASP	No	1	387	45	8.5	5	4	16
02153	BRE4_CAEEL	Glyco_transf_7N, Glyco_transf_7C, Glyco_tranf_2_2	AIP	Yes	1	351	40	9.5	5	7	9
02308	R7TKQ2_CAPTE	ND	AIP	Yes	1	664	75	6.2	5	6	11
03132	COLL4_MIMIV	Collagen	AIP	Yes	1	605	62	9.5	5	21	10
03669	ATL1_MOUSE	TSP_1, I-set, Ig_2	ASP	Yes	1	1058	117	7.9	6	7	11
04974	CHSS1_HUMAN	CHGN	Both	Yes	1	333	39	8.5	3	6	13
05522	TNR16_HUMAN	TNFR_c6, Death	AIP	Yes	1	396	43	8.6	6	7	9
05602	PHM_CAEEL	Cu2_monoox_C, Cu2_monooxygen	AIP	Yes	1	340	38	5.9	8	8	12
05786*	CHIT3_DROME	Glyco_hydro_18	AIP	Yes	5	1089	117	4.9	9	8	10
05787*	CHIT3_DROME	CBM_14	AIP	No	1	860	93	9.5	7	4	5
05788	CHIT3_DROME	Glyco_hydro_18, CBM_14	Both	Yes	1	2120	232	9.2	8	6	8
06725	COLA1_HUMAN	VWA, VWA_2, TSP_1, VWA_CoxE	Both	Yes	5	437	48	10.3	8	7	8
07695*	FBN2_HUMAN	EGF_CA, cEGF, EGF, FXa_inhibition, EGF_3, TSP_1	Both	No	5	3384	355	4.4	7	8	8
07696*	HMCN1_HUMAN	TSP_1	AIP	Yes	4	1021	110	5.0	5	12	11
08180*	C3XSB7_BRAFL	ND	AIP	Yes	1	151	17	6.3	5	8	10
08475	CDHR1_CHICK	Cadherin	ASP	Yes	1	662	73	4.3	5	6	14
08509*	GPX3_RAT	GSHPx	Both	Yes	5	200	22	8.3	6	9	9
09129	PA2A2_VIPRE	Phospholip_A2_1, Parvo_coat_N	AIP	Yes	1	240	27	9.2	6	11	9
09130	PA2A2_VIPRE	Phospholip_A2_1	Both	Yes	1	128	14	8.8	10	8	9
10213	GRM7_PONAB	ANF_receptor, Peripla_BP_6, 7tm_3	AIP	Yes	1	2826	309	5.7	8	8	9
10732	K1PV37_CRAGI	ND	Both	Yes	1	217	24	5.4	6	5	11
11625	C3ZH66_BRAFL	ND	ASP	No	1	854	96	8.9	5	5	9

(*Continued*)

Supplementary Table 25 Continued

Gene ID[a]	Best hit to UniProt	Pfam domain(s)	Fraction[b]	Signal peptide	Unique peptide hit(s)	Length	MW (kDa)	pI	A (%)	G (%)	Acidic (%)[c]
13290	COKA1_HUMAN	VWA, VWA_2, VWA_3	AIP	Yes	8	310	33	7.0	11	11	10
14202	CO6A6_HUMAN	VWA, VWA_2	AIP	Yes	2	429	49	9.1	9	5	13
14618*	HMCN1_HUMAN	TSP_1	AIP	Yes	1	423	44	5.0	8	14	9
17440	PDIA6_RAT	Thioredoxin, Thioredoxin_2, Thioredoxin_8, Thioredoxin_6	ASP	Yes	1	442	48	5.3	9	10	14
17613*	CO4A1_CAEEL	Collagen, EGF	AIP	Yes	5	795	71	11.3	19	31	4
17614*	CO4A2_ASCSU	Collagen, EGF	AIP	Yes	2	774	68	10.5	20	31	5
17615*	CO4A2_ASCSU	Collagen. EGF	AIP	Yes	6	781	69	9.4	21	30	8
19406	TENX_HUMAN	Laminin_G_3, VWD, F5_F8_type_C, Pentaxin	AIP	Yes	1	21010	2242	5.7	6	13	9
19546	CTHR1_HUMAN	PAN_1	AIP	Yes	1	446	49	6.0	6	9	9
20759*	VWA2_HUMAN	VWA, VWA_2, DUF1194	Both	Yes	6	246	26	8.7	11	10	8
20760*	VWA1_HUMAN	VWA, VWA_2, DUF1194	Both	Yes	3	246	26	8.4	11	10	9
20929*	CO4A2_ASCSU	Collagen, EGF	AIP	Yes	4	1548	145	9.8	10	30	6
21526	ALG8_HUMAN	Alg6_Alg8	ASP	Yes	1	525	60	9.1	7	6	5
21648*	PXDN_XENTR	An_peroxidase	AIP	Yes	2	927	106	8.6	6	6	13
22439*	K1QDK3_CRAGI	VWA, VWA_2, DUF1194	AIP	Yes	1	246	26	8.4	11	10	8
22634*	NFH_MOUSE	ND	Both	Yes	8	530	53	8.7	38	2	14
23591*	MSMB_DORPE	PSP94	AIP	Yes	1	196	21	5.1	5	11	11
24135*	HEPH_HUMAN	ND	AIP	Yes	4	421	47	6.0	7	9	12
24136	HEPH_MOUSE	Cu-oxidase_3, Cu-oxidase_2	Both	No	8	648	73	5.4	6	7	14
27080	MUC5B_HUMAN	Mucin2_WxxW, F5_F8_type_C, VWD, C8, TIL	AIP	Yes	1	7124	753	6.3	4	8	10
28318	CD109_HUMAN	A2M_N_2, A2M, A2M_N, Thiol-ester_cl	Both	No	6	1007	111	5.6	6	7	11
28319	CD109_HUMAN	A2M_comp, Prenyltrans_2, Thiol-ester_cl, Prenyltrans_1	AIP	No	1	878	96	5.6	8	8	10
28520	FAT4_HUMAN	Cadherin	Both	Yes	7	5471	607	5.3	6	6	11
28818*	CO5A2_MOUSE	ND	Both	Yes	7	1159	108	4.5	7	22	4
29907	SAP_HUMAN	SapB_2, SapB_1	ASP	Yes	1	696	76	4.9	4	7	11
30054	COKA1_HUMAN	VWA, VWA_2, VWA_3	AIP	Yes	3	340	35	7.6	11	14	9

[a]Genes highly or specifically expressed in the mantle tissue are labeled with asterisks (*). [b]AIP, acid insoluble proteins; ASP, acid souble proteins; Both, proteins in both AIP and ASP fractions. [c]Acidic amino acids counted by number of total aspartate and glutamate. MW, molecular weight. ND, not detected.

Supplementary Table 26 | Characterization of *Lingula* SMPs with no detectable homology

Gene ID[a]	Best hit to UniProt	Pfam domain	Fraction[b]	Signal peptide	Unique peptide hit(s)	Length	MW (kDa)	pI	A (%)	G (%)	Acidic (%)[c]
09615*	No-hit	ND	AIP	Yes	1	159	18	7.0	6	8	12
11493	No-hit	ND	AIP	Yes	3	123	12	10.3	56	2	2
11626*	No-hit	ND	AIP	No	1	103	12	9.9	12	2	12
12756	No-hit	ND	ASP	Yes	1	200	23	9.9	4	6	6
14146	No-hit	Shisa	AIP	Yes	1	145	16	4.9	7	6	15
18759	No-hit	ND	Both	Yes	1	103	10	10.3	43	9	5
18760*	No-hit	ND	Both	Yes	9	242	22	9.6	57	5	8
18761*	No-hit	ND	AIP	Yes	3	253	23	9.7	53	6	7
20455*	No-hit	ND	AIP	Yes	1	113	12	7.8	12	12	7
21207*	No-hit	ND	AIP	Yes	7	237	23	5.0	36	3	15
25838*	No-hit	ND	AIP	Yes	1	210	23	8.1	10	6	7
26937*	No-hit	ND	AIP	Yes	5	150	17	5.4	7	5	12
28631*	No-hit	ND	Both	Yes	3	114	13	10.1	11	9	5
31064*	No-hit	ND	Both	Yes	1	107	12	8.7	12	9	6

[a]Genes highly or specifically expressed in the mantle tissue are labeled with asterisks (*). [b]AIP, acid insoluble proteins; ASP, acid souble proteins; Both, proteins in both AIP and ASP fractions. [c]Acidic amino acids counted by number of total aspartate and glutamate. MW, molecular weight. ND, not detected.

Supplementary Table 27 | Summary of domains found in *Lingula* SMPs

Pfam ID	Count	Pfam accession	Description
Cadherin	52	PF00028.12	Cadherin domain
Collagen	40	PF01391.13	Collagen triple helix repeat (20 copies)
TSP_1	37	PF00090.14	Thrombospondin type 1 domain
VWD	19	PF00094.20	von Willebrand factor type D domain
EGF_CA	17	PF07645.10	Calcium-binding EGF domain
EGF	17	PF00008.22	EGF-like domain
FXa_inhibition	13	PF14670.1	Coagulation Factor Xa inhibitory site
cEGF	13	PF12662.2	Complement Clr-like EGF-like
CBM_14	13	PF01607.19	Chitin binding Peritrophin-A domain
C8	13	PF08742.6	C8 domain
VWA_2	12	PF13519.1	von Willebrand factor type A domain
VWA	12	PF00092.23	von Willebrand factor type A domain
Mucin2_WxxW	11	PF13330.1	Mucin-2 protein WxxW repeating region
EGF_3	10	PF12947.2	EGF domain
Laminin_G_3	8	PF13385.1	Concanavalin A-like lectin/glucanases superfamily
F5_F8_type_C	8	PF00754.20	F5/8 type C domain
Sushi	6	PF00084.15	Sushi domain (SCR repeat)
SapB_1	6	PF05184.10	Saposin-like type B, region 1
GCC2_GCC3	6	PF07699.8	GCC2 and GCC3
VWA_3	5	PF13768.1	von Willebrand factor type A domain
TIL	5	PF01826.12	Trypsin Inhibitor like cysteine rich domain
Peripla_BP_6	5	PF13458.1	Periplasmic binding protein
HYR	5	PF02494.11	HYR domain
ANF_receptor	5	PF01094.23	Receptor family ligand binding region
TNFR_c6	4	PF00020.13	TNFR/NGFR cysteine-rich region
Phospholip_A2_1	4	PF00068.14	Phospholipase A2
Glyco_hydro_18	4	PF00704.23	Glycosyl hydrolases family 18
I-set	3	PF07679.11	Immunoglobulin I-set domain
DUF1194	3	PF06707.6	Protein of unknown function (DUF1194)
CUB	3	PF00431.15	CUB domain
Thioredoxin	2	PF00085.15	Thioredoxin
Thiol-ester_cl	2	PF10569.4	Alpha-macro-globulin thiol-ester bond-forming region
SapB_2	2	PF03489.12	Saposin-like type B, region 2
PSP94	2	PF05825.6	Beta-microseminoprotein (PSP-94)
Parvo_coat_N	2	PF08398.5	Parvovirus coat protein VP1
Cu-oxidase_3	2	PF07732.10	Multicopper oxidase
A2M_comp	2	PF07678.9	A-macroglobulin complement component
zf-C4	1	PF00105.13	Zinc finger, C4 type (two domains)
VWA_CoxE	1	PF05762.9	VWA domain containing CoxE-like protein
V-set	1	PF07686.12	Immunoglobulin V-set domain
Trypsin	1	PF00089.21	Trypsin
Thioredoxin_8	1	PF13905.1	Thioredoxin-like
Thioredoxin_6	1	PF13848.1	Thioredoxin-like domain
Thioredoxin_2	1	PF13098.1	Thioredoxin-like domain

(*Continued*)

Supplementary Table 27 Continued

Pfam ID	Count	Pfam accession	Description
Shisa	1	PF13908.1	Wnt and FGF inhibitory regulator
SEA	1	PF01390.15	SEA domain
Prenyltrans_2	1	PF13249.1	Prenyltransferase-like
Prenyltrans_1	1	PF13243.1	Prenyltransferase-like
Pentaxin	1	PF00354.12	Pentaxin family
PAN_1	1	PF00024.21	PAN domain
Ldl_recept_a	1	PF00057.13	Low-density lipoprotein receptor domain class A
Ig_2	1	PF13895.1	Immunoglobulin domain
Hormone_recep	1	PF00104.25	Ligand-binding domain of nuclear hormone receptor
GSHPx	1	PF00255.14	Glutathione peroxidase
Glyco_transf_7N	1	PF13733.1	N-terminal region of glycosyl transferase group 7
Glyco_transf_7C	1	PF02709.9	N-terminal domain of galactosyltransferase
Glyco_tranf_2_2	1	PF10111.4	Glycosyltransferase like family 2
Death	1	PF00531.17	Death domain
Cu-oxidase_2	1	PF07731.9	Multicopper oxidase
Cu2_monooxygen	1	PF01082.15	Copper type II ascorbate-dependent monooxygenase, N-terminal domain
Cu2_monoox_C	1	PF03712.10	Copper type II ascorbate-dependent monooxygenase, C-terminal domain
CHGN	1	PF05679.11	Chondroitin N-acetylgalactosaminyltransferase
Big_3_4	1	PF13754.1	Bacterial Ig-like domain (group 3)
An_peroxidase	1	PF03098.10	Animal haem peroxidase
Alg6_Alg8	1	PF03155.10	ALG6, ALG8 glycosyltransferase family
A2M_N_2	1	PF07703.9	Alpha-2-macroglobulin family N-terminal region
A2M_N	1	PF01835.14	MG2 domain
A2M	1	PF00207.17	Alpha-2-macroglobulin family
7tm_3	1	PF00003.17	7 transmembrane sweet-taste receptor of 3 GCPR

Supplementary Table 28 | Summary of SMPs highly expressed in the *Lingula* mantle tissue

Gene ID	Entry[a]	Entry name[b]	Protein names	Function
01003	P0C6B8	SVEP1_RAT	Sushi, von Willebrand factor type A, EGF and pentraxin domain-containing protein 1	Calcium ion binding and cell attachment
05786	Q9W5U2	CHIT3_DROME	Probable chitinase 3	Hydrolysis of N-acetyl-beta-D-glucosaminide (1->4)-beta-linkages in chitin
05787	Q9W5U2	CHIT3_DROME	Probable chitinase 3	Hydrolysis of N-acetyl-beta-D-glucosaminide (1->4)-beta-linkages in chitin
07695	P35556	FBN2_HUMAN	Fibrillin-2	Extracellular calcium-binding microfibrils and regulating osteoblast maturation
07696	Q96RW7	HMCN1_HUMAN	Hemicentin-1	Calcium ion binding protein with multiple roles
08180	C3XSB7	C3XSB7_BRAFL	Putative uncharacterized protein	Unknown
08509	P23764	GPX3_RAT	Glutathione peroxidase 3	Protecting cells and enzymes from oxidative damage
14618	Q96RW7	HMCN1_HUMAN	Hemicentin-1	Calcium ion binding protein with multiple roles
17613	P17139	CO4A1_CAEEL	Collagen alpha-1(IV) chain	Specific for basement membranes with multiple roles
17614	P27393	CO4A2_ASCSU	Collagen alpha-2(IV) chain	Specific for basement membranes with multiple roles
17615	P27393	CO4A2_ASCSU	Collagen alpha-2(IV) chain	Specific for basement membranes with multiple roles
20759	Q5GFL6	VWA2_HUMAN	von Willebrand factor A domain-containing protein 2	Promoting matrix assembly
20760	Q6PCB0	VWA1_HUMAN	von Willebrand factor A domain-containing protein 1	Promoting matrix assembly
20929	P27393	CO4A2_ASCSU	Collagen alpha-2(IV) chain	Specific for basement membranes with multiple roles
21648	A4IGL7	PXDN_XENTR	Peroxidasin	Extracellular matrix consolidation, phagocytosis and defense
22439	K1QDK3	K1QDK3_CRAGI	Collagen alpha-1(XII) chain	Unknown
22634	P19246	NFH_MOUSE	Neurofilament heavy polypeptide	Intermediate filament maintaining of neuronal caliber
23591	D2X5V5	MSMB_DORPE	Beta-microseminoprotein	Acting as a pheromone
24135	Q9BQS7	HEPH_HUMAN	Hephaestin	May function as a ferroxidase for ferrous (II) to ferric ion (III) conversion and may be involved in copper transport and homeostasis
28818	Q3U962	CO5A2_MOUSE	Collagen alpha-2(V) chain	A key determinant in the assembly of tissue-specific matrices

[a]UniProt entry ID. [b]UniProt entry name.

Supplementary Table 29 | The 20 most abundant domains combined with EGF domains in selected bilaterians

Human		*Lingula*		Sea snail		Pacific oyster		Pearl oyster	
Domain	#	Domain	#	Domain	#	Domain	#	Domain	#
EGF_CA	71	EGF_CA	135	EGF_CA	52	EGF_CA	113	EGF_CA	81
FXa_inhibition	52	EGF_3	102	EGF_2	43	hEGF	78	EGF_2	68
cEGF	52	cEGF	99	hEGF	41	EGF_2	70	hEGF	61
EGF_3	51	FXa_inhibition	93	EGF_3	36	EGF_3	65	EGF_3	51
Laminin_G_2	27	hEGF	52	FXa_inhibition	31	FXa_inhibition	55	cEGF	42
Laminin_G_1	25	EGF_2	48	cEGF	31	cEGF	44	FXa_inhibition	40
hEGF	25	Sushi	29	Laminin_G_2	14	CUB	25	Laminin_G_3	18
EGF_2	24	Laminin_G_3	27	Laminin_G_3	13	MAM	18	Laminin_G_2	14
Laminin_G_3	14	Laminin_G_2	26	Laminin_G_1	12	Astacin	17	MAM	12
Sushi	13	EGF_MSP1_1	26	Laminin_EGF	5	VWA_2	16	Laminin_G_1	12
**Trypsin†**	11	Laminin_G_1	22	HYR	5	Laminin_G_2	16	GCC2_GCC3	12
CUB	9	**Collagen†**	17	CUB	5	VWA	15	TSP_1	11
SGL	8	TSP_1	15	Sushi	4	Sushi	13	EGF_MSP1_1	11
Lectin_C	8	HYR	15	**Ldl_recept_a†**	4	**F5_F8_type_C†**	13	**DSL†**	11
**Ldl_recept_b†**	8	GCC2_GCC3	15	GCC2_GCC3	4	Laminin_G_1	12	CUB	11
Laminin_EGF	8	Lectin_C	14	EGF_MSP1_1	4	**VWA_3†**	11	VWA	10
VWA_2	7	CUB	13	**Cadherin†**	4	TSP_1	11	VWA_2	10
**V-set†**	7	**SEA†**	12	**Cadherin_2†**	4	GCC2_GCC3	11	Sushi	9
**TB†**	7	VWD	11	SGL	3	Laminin_G_3	10	**I-set†**	9
**Gla†**	7	**VWA†**	11	**Pentaxin†**	3	EGF_MSP1_1	10	Astacin	9

Domains that are highly and specifically abundant in particular species are highlighted in bold and labeled with daggers (†). *Lingula* domains are highlighted in grey.

Supplementary Table 30 | The 10 most abundant domains combined with Collagen domains in selected bilaterians

Human		*Lingula*		Sea snail		Pacific oyster		Pearl oyster	
Domain	#	Domain	#	Domain	#	Domain	#	Domain	#
C1q	22	EGF_CA	22	**COLFI†**	3	Ig_3	5	EGF_CA	7
VWA	12	FXa_inhibition	20	C4	3	Ig_2	5	FXa_inhibition	6
VWA_2	12	cEGF	19	Glutenin_hmw	2	V-set	4	cEGF	6
**COLFI†**	11	EGF*	17	DUF4402	2	I-set	4	I-set	4
VWA_3	9	C1q	10	DUF3060	2	ig	4	Ig_2	4
Laminin_G_3	8	EGF_3	6	TNF	1	C4	4	MAM	3
Lectin_C	7	C4	6	Ribosomal_L6	1	**COLFI†**	3	ig	3
Laminin_G_2	7	SRCR	5	Plasmodium_HRP	1	MAM	2	EGF*	3
C4	6	TSP_1	4	Laminin_G_3	1	VWC	1	**COLFI†**	3
VWC	4	PAN_1	4	Laminin_G_2	1	Peptidase_M13	1	V-set	2

Fibrillar collagen C-terminal domain (COLFI) that is present in collagen genes for vertebrate bone formation is highlighted in bold and labeled with daggers (**†**). Epidermal growth factor-like domain (EGF) that is found in the *Lingula* shell matrix, is underlined and labeled with asterisks (*). *Lingula* domains are highlighted in grey.

Supplementary Table 31 | Summary of genes reported to be involved in shell and bone formation.

Category	Gene name	Function	Shell formation in mollusc species	Mollusc shell formation	Vertebarte bone formation
Shell and bone formation	BMP2/4	Ligand of BMP signaling	*Patella vulgata*	Nederbragt et al., 2012[27]	Chen et al., 2012[28]
	BMPR	Receptor of BMP signaling	*Pinctada martensii*	Yan et al., 2014[29]	Chen et al., 2012[28]
	Smad1/5/9	Regulatory mediator of BMP signaling	*Crassostrea gigas*	Liu et al., 2014[30]	Chen et al., 2012[28]
	Smad4	Co-mediator of BMP signaling	*Crassostrea gigas*	Liu et al., 2014[30]	Chen et al., 2012[28]
	Engrailed	Homeodomain transcription factor	*Lymnaea stagnalis*	Iijima et al., 2008[31]	Deckelbaum et al., 2006[32]
	Calcineurin†	Calcium-dependent serine-threonine phosphatase	*Pinctada fucata*	Li et al., 2010[33]	Sun et al., 2005[34]
	Calponin†	Calcium binding protein	*Pinctada martensii*	Shi et al., 2013[35]	Su et al., 2013[36]
	Calmodulin†	Calcium-binding messenger	*Pinctada fucata*	Yan et al., 2007[37]	Zayzafoon et al., 2005[38]
	Cadherin†	Transmembrane junction	*Crassostrea gigas*	Zhang et al., 2012[9]	Marie, 2002[39]
	Carbonic anhydrase	Catalyzing CO2 to bicarbonate	*Pinctada fucata*	Miyamoto et al., 1996[40]	Lehenkari et al., 1998[41]
	ECM collagen	ECM component	*Crassostrea gigas*	Zhang et al., 2012[9]	Nudelman et al., 2010[42]
	Fibronectin	ECM component binds to integrins	*Crassostrea gigas*	Zhang et al., 2012[9]	Bentmann et al., 2010[43]
Shell formation	Hox4	Homeodomain transcription factor	*Gibbula varia*	Samadi and Steiner, 2009[44]	NA
	Tyrosinase	Formation of melanin from tyrosine	*Pinctada fucata*	Zhang et al., 2006[45]	NA
	Chitin synthase	Synthesizing chitin	*Atrina rigida*	Weiss et al., 2006[46]	NA
	Chitinase	Degrading chitin	*Lottia gigantea*	Marie et al., 2013[47]	NA
	Perlucin†	Carbohydrate binding	*Haliotis laevigata*	Mann et al., 2000[48]	NA
	Peroxidasin†	Peroxidase with ECM motif	*Lottia gigantea*	Marie et al., 2013[47]	NA
	VWA	Cell adhesion	*Lottia gigantea*	Marie et al., 2013[47]	NA
	Mucin	Glycosylated protein for gel forming	*Pinna nobilis*	Marin et al., 2000[49]	NA
Lingula specific	EGF collagen fiber	Unknown	NA	NA	NA
	Alanine-rich fiber	Unknown	NA	NA	NA
Bone formation	Carbohydrate sulfotransferase	Transferring sulfate to chondroitin	NA	NA	Hermanns et al., 2008[50]
	Fibrillin†	Forming elastic fibers	NA	NA	Nistala et al., 2010[51]
Not determined	Glutathione peroxidase	Reduction of hydroxyperoxides	NA	NA	NA
	Hephaestin	Metabolism of copper	NA	NA	NA
	Hemicentin†	Extracellular immunoglobulin	NA	NA	NA
	SVEP1†	Cell attachment	NA	NA	NA

Abbreviations: BMP, bone morphogenetic protein; ECM, extracellular matrix; VWA, von Willebrand factor type A; EGF epidermal growth factor; SVEP1, Sushi, von Willebrand factor type A, EGF and pentraxin domain-containing protein 1. Genes with calcium binding domains are labeled with daggers (†). NA, study not available.

Supplementary Notes

Supplementary Note 1: Background information, materials and methods

1.1. Background information of the brachiopod *Lingula anatina*

Although superficially resembling mussels, lingulid (i.e., tongue-shaped) brachiopods, including *Lingula anatina,* have several unique features that distinguish them from bivalves. These include flexible, dorso-ventral shells made of calcium phosphate without hinges, chitinous chaetae on the mantle margins, two arms lined with ciliated tentacles (i.e., lophophores) for filter feeding, and a tail-like structure (i.e., pedicle) to attach to hard substrate[52,53] (Fig. 1a). In addition, their early embryonic development is like that of basal deuterostomes[54] (i.e., radial cleavage and enterocoely; Fig. 1b-i). With inarticulate shells, *Lingula* has evolved to adapt to an infaunal lifestyle, such as burrowing into the sand in a U-shaped manner, positioning themselves vertically, and living in the intertidal zone[55,56]. Importantly, their lingulid shell shows some of the very first innovations in animal biomineralization, since the fossil record of lingulid brachiopods dates back more than 520 million years ago (MYA)[57]. It seems reasonable that lingulid brachiopods might have taken advantage of calcium phosphate, since the phosphorus concentration in the seawater was ostensibly high during the Precambrian and Cambrian Periods[58]. Since the Permian extinction, bivalves have rapidly increased their diversity, but the basic body plan of brachiopods has been constrained[59]. The origin of differences between brachiopods and bivalves is still a mystery.

Darwin first noticed *Lingula* (possibly referring to all then known lingulid brachiopods) while comparing abundant fossils to living species. He concluded that their shells have changed very little since the early Cambrian, compared to bivalves and referred to them as an example of "living fossils"[60]. However, this idea is still controversial[61,62]. Detailed examination of fossilized and living shells of lingulid brachiopods shows that there is a high diversity on their chemical structure (i.e., how the minerals growth and arrange within the shell)[63,64]. Similar to this line, soft tissue fossils found in the Chengjiang fauna show that there have been morphological changes among lingulid brachiopods, suggesting that they evolved in contrast to the idea of that "the Silurian Lingula differs but little from the living species" by Darwin thought[65]. This notion is supported by population genetics of *L. anatina* across the Indo-West Pacific region, which exhibits a high genetic divergence within the same species[66].

In order to answer some of the questions mentioned above, we sequenced the genome, transcriptomes, and shell proteome of the lingulid brachiopod, *Lingula anatina*. Using these data, we applied comparative genomic analyses to provide insights into the evolutionary history of this

lophotrochozoan and the origin of phosphate biomineralization (A full list of proteomes and genomes of selected metazoans used in this study is shown in Supplementary Table 1).

1.2. Biological materials

Gravid *Lingula* adults (Fig. 1a) were collected during July to August in Kasari Bay, Amami Island (28.440583 N 129.667608 E; Supplementary Fig. 1a). Mature male gonads were dissected for genomic DNA extraction. Maturation of oocytes was induced by injection of 30 µl of 40 mM dibutyryl-cAMP (in phosphate-buffered saline, PBS) into the gonad[67]. Artificial spawning was performed by elevating the temperature to 29°C for 2-6 h[68] followed by cold shock back to room temperature (~25°C) for several cycles. Fountain-like spawning behavior can be observed as reported before[69], in which the gametes are ejected via the middle pseudosiphon. Embryonic development was monitored and staged according to Yatsu (1902)[54] as shown in Fig. 1b-i. For studying early development and providing transcript evidence for gene model prediction, ten embryonic stages from fertilized egg to 2-pair-cirri larva were collected and subjected to total RNA extraction with TRIzol.

In order to study the function of mantle tissue, which is responsible for biomineralization, we sampled adult tissues. An adult individual with a shell length of 4 cm was dissected. Seven tissues including the dorsal mantle, ventral mantle, lophophore, gut, digestive cecum, pedicle, and regenerating pedicle (one month post amputation) were collected. Dissected tissues were washed with filtered seawater and then rinsed with PBS. After adding TRIzol, the tissues were first ground with plastic micropestle. Larger tissues were cut into small pieces with surgical scissors. The tissues were then completely homogenized with a Polytron handheld homogenizer. Homogenized samples were centrifuged and supernatants were used for RNA extraction with the standard TRIzol protocol. The RNA quality of both embryonic and adult extracts was checked with an Agilent Technologies 2100 Bioanalyzer using an Agilent RNA 6000 Nano Kit.

1.3. Genome sequencing, assembly, gene modeling, and annotation

Sequencing, assembly and annotation of the *Lingula anatina* genome are shown in Supplementary Fig. S2. To avoid contamination with environmental microbes, we extracted genomic DNA from a gravid male gonad (i.e., mostly sperm cells). We sequenced the *Lingula* genome with next-generation sequencing (NGS) with a hybrid approach using three different platforms. The genome was sequenced by the shotgun method using NGS platforms: Roche 454 GS FLX+[70], Illumina (MiSeq and HiSeq 2500)[71], and PacBio RS II[72]. Sequencing quality was checked with FastQC (v0.10.1)[73]. MiSeq reads with duplication and low-complexity were removed with PRINSEQ (v0.20.3)[74]. Raw Illumina reads were quality filtered (Q20, 99%

accuracy) and trimmed 5-10 bp on both ends to remove sequencing bias and low quality bases using Trimmomatic (v0.30)[75]. Raw mate pair reads were filtered with DeLoxer[76] (using version of R 2.12.1 is recommended) or NextClip (v0.8)[77] depending on library preparation.

Genome assembly was conducted using Newbler (v2.9, an overlap-based assembler) with a hybrid assembly approach using data from 454 and Illumina[10,14]. First, after preparation of a 1,750 bp library, we sequenced 17 runs of this library using a Roche GS FLX+[70]. This generated 9.6 Gb data with an average read length of 520 bp (~23X of coverage) (Supplementary Figs 1c and 2a and Supplementary Table 2). Second, taking advantage of the enhancement of the read length in Illumina technology[71], we prepared libraries in size ranging from 500 to 620 bp and sequenced 32.5 Gb of 250 bp long paired-end data using an Illumina MiSeq (~76X) (Supplementary Fig. S2a and Supplementary Table S2). To overcome repetitive regions of the genome, we prepared 1.5-3 kb mate pair libraries by Cre-Lox recombination approach[76]. In addition, in order to produce a long mate pair library, we used the BluePippin system to prepare 5-17 kb DNA fragments and constructed libraries by using Nextera technology[78]. We sequenced these libraries to obtain 45.5 Gb of mate pair data using a MiSeq and a HiSeq 2500 of Illumina, which have read lengths of 300 and 150 bp, respectively (~107X) (Supplementary Table 2).

Finally, Illumina mate pair reads together with 8.5 Gb of PacBio extra-long reads (7-38 kb, ~20x) were used for scaffolding. Scaffolding was accomplished by mapping paired-end and mate pair reads (1.5-17 kb) from Illumina with SSPACE (v3.0)[79]. PacBio long reads (>7 kb) were mapped to the Newbler generated scaffolds with BLASR (v20141001)[80], and upgraded scaffolds were produced with SSPACE-LongRead (v1-1)[81] (Supplementary Table 2). Gaps in the scaffolds were then filled using GapCloser (v1.12-r6) from the SOAPdenovo2 package (r240)[82] (Supplementary Fig. 2a). Redundancy of the final scaffolds was removed using a custom Perl script (calculating BLASTN alignment length and identity, Chuya Shinzato, personal communication)[14]. After gap closing, there were 17.5 Mb of gaps (4.1%) in the final assembly (Supplementary Fig. 2d). The estimated size of the *Lingula* genome was approximately 463 Mb, based on flow cytometry (Supplementary Fig. 1b). To estimate the genome size further, we performed K-mer analysis with SOAPec (v2.01) and Genomic Character Estimator (GCE; v1.0.0) from the SOAPdenovo package[82]. We also counted the K-mers using Jellyfish (v2.0.0)[83] and conducted the analysis with a custom Perl script. These two methods generated similar results, namely, approximately 410 Mb (Supplementary Fig. 1d).

Heterozygosity rate of the *Lingula* genome was 1.6% based on calculation of the ratio of homozygous and heterozygous peaks (Supplementary Fig. 1d), meaning that SNP occurs about once every 62 bp. This ratio is higher than that of humans (0.043%). Regions of repetitive sequences were identified with RepeatScout (v1.0.5)[84] and then masked with RepeatMasker

(v4.0.3)[85]. The *Lingula* genome is less repetitive (22.2%) than the pearl oyster genome[10] (Supplementary Fig. 2d; see also Supplementary Note 3.5). The *Lingula* genome shows low GC content (36.3%), which is similar to mollusc genomes (Supplementary Fig. 1e,f). The final assembly size of the *Lingula* genome was 425 Mb, which was in the range predicted by two different types of estimate. Based upon this genome size, we sequenced the *Lingula* geneome with approximately 226-fold coverage.

The quality and completeness of the genome assembly were assessed by searching for the set of 248 core eukaryotic genes using CEGMA (v2.4.010312)[86] and by mapping back mRNA transcripts to the genome assembly with BLAT (v.35)[87] and baa.pl[88]. The CEGMA analysis shows that the completeness of the current version of genome assembly is comparable to that of published genomes of marine invertebrate genomes (Supplementary Fig. 3 and Supplementary Table 3). Further evaluation of the current assembly quality by mapping back transcriptome data to the assembled genome shows that 99.3% of transcripts have BLAT entries, indicating a high quality genome.

To obtain high quality gene models, we performed deep mRNA sequencing (RNA-seq) to obtain transcript information (Supplementary Figs 2b and 4). Gene models were predicted with trained AUGUSTUS (v3.0.2)[89] using hints from spliced alignment of transcripts to the masked genome assembly produced with BLAT[87] and PASA (r20130907)[90] (Supplementary Fig. 2c). There are 34,105 gene models predicted from the repeat-masked genome, which is higher than other lophotrochozoans[8,9] (Supplementary Fig. 1d). A BLAST top-hits search against the NCBI nr database using BLAST+ (v2.2.29+; e-value, 1e-5)[91] and Blast2GO[92] shows that 28% of the *Lingula* gene models have best hits among molluscs, implicating a close relationship between *Lingula* and molluscs (Supplementary Fig. 5). On the other hand, 21% of the genes show no hits to known sequences, suggesting these genes may specifically pertain to the *Lingula* lineage (Supplementary Fig. 5); however, we cannot exclude the possibility of overestimation in which gene model errors may contribute to this estimate. In agreement with BLAST top-hits results, *Lingula* has an average gene size of 6.7 kb with 6.6 introns per gene (Supplementary Fig. 2d), which is closer to the sea snail, *Lottia*, than to the leech, *Helobdella*, or the polychaete, *Capitella*[8].

1.4. Transcriptome analysis of embryos and adult tissues

To study the spatiotemporal expression of genes, RNA-seq of 369 M read pairs from embryos and adult tissues was conducted with an Illumina HiSeq 2500 (Supplementary Fig. 4 and Supplementary Table 4). Transcripts were assembled *de novo* with Trinity (r2013_08_14, a de Bruijn graph-based program)[93,94] and used as an expression evidence for gene model prediction

(Supplementary Fig. 2b). In addition, to allow the transcriptome more accessible for downstream analysis, we eliminated transcript assemblies that contained computation errors, expressed at extremely low levels, and expressed with highly similar isoforms. After RNA-seq assembly, we mapped back all Q20 reads from each embryonic stage and adult tissue using Bowtie (v2.1.0)[95], followed by estimation of the transcript abundance using RSEM (v1.2.5)[96]. We filtered transcripts using the criteria that the expression level of fragments per kilobase of transcript per million mapped reads (FPKM) lower than one and isoform appearance less than 5%. In addition, redundant isoforms were removed with CD-HIT (v4.6)[97] using 95% identity as a criterion. This step removed 61% of the transcripts from the primary assembly (Supplementary Fig. 4b, secondary assembly).

Next, we applied three sets of criteria to select transcripts with annotated biological functions. First, open reading frames (ORFs) of transcripts were extracted with the program, getorf, in the EMBOSS package (v6.6.0.0)[98]. We retained transcripts with ORFs longer than 70 amino acids. Next, we searched the transcriptome against the Pfam database (Pfam-A 27.0)[99] with HMMER (v3.1b1)[100] and against UniProtKB database[101] with BLASTP, respectively. The final representative "best" assembly is the union of three sets of transcripts, which gave rise to a 101 Mb transcriptome with N50 size of 2,955 bp for 47,943 transcripts (removal 86% of transcripts from the primary assembly) (Supplementary Fig. 4b,c). In order to assess the quality of the transcriptome assembly, we applied full-length transcript analysis using a bundled Perl script "analyze_blastPlus_topHit_coverage.pl" in the Trinity package[94]. The *Lingula* transcriptome is of high quality in terms of the number of full-length transcripts, which is comparable to the best annotated animals and is the best when selected gene models and the transcriptome are compared (Supplementary Fig. 4d).

1.5. Proteomic analyses of shell matrix proteins

To provide insights into biomineralization in one of the most ancient animals, we used a proteomic approach to study the *Lingula* shell matrix. The shell of one *Lingula* adult was dissected and stirred in 12.5% NaClO. Soft tissue remaining on the shells was removed with Milli-Q water. The cleaned shell was mechanically crushed and ground into fine powder, and then treated with 12.5% NaClO to remove remaining contaminants. In order to remove minerals and classify the acidic solubility of shell matrix proteins, the shell powder was decalcified with 1 M acetic acid overnight. Acid soluble proteins (ASP) from the supernatant were precipitated by adding chloroform/methanol/Milli-Q water (1:1/4/3). After mixing and centrifuging, the same amount of methanol was added for washing the pellet. The pellet contained ASP was re-suspended in the reducing buffer (1% SDS, 10 mM DTT, 50 mM pH 8.0 Tris-HCl).

On the other hand, the insoluble pellet from the acetic acid solution, which contained acid insoluble proteins (AIP), was rinsed with Milli-Q water and then re-suspended in reducing buffer. ASP and AIP samples were mixed with sample buffer, respectively, and subjected to SDS-PAGE. Extracted shell matrix proteins (SMPs) were resolved in a 10-20% gradient gel, visualized with SimplyBlue SafeStain and SYPRO Ruby staining. Afterward, protein bands were excised and in-gel digested with trypsin. Peptides were identified with LC-MS/MS performed as previously described[102].

In brief, digested peptides were analyzed using a capillary liquid chromatography system (Dionex, UltiMate 3000) connected to a mass spectrometer (Thermo Scientific, LTQ-XL). Raw spectra were processed using SEQUEST software to extract peak lists[103]. Resulting peak lists were analyzed using an in-house MASCOT (v2.3.2) server against *Lingula* predicted gene models. We did not apply the "two-peptide" rule here since we noticed that this approach introduces bias and often leads to loss of information[104]. Instead, peptide-hits were quality-filtered using ion score significance thresholds (>45; i.e., false discovery rate, FDR < 0.05), and high-quality one-peptide hits were retained.

Lingula SMPs were first analyzed in regard to molecular weight, theoretical pI, and amino acid composition with ProtParam[105] using a custom Python script with the module, "Bio.SeqUtils.ProtParam" from Biopython. They were then searched against the NCBI nr database by BLASTP to assign homology and possible function. Furthermore, they were categorized into secreted and non-secreted proteins using SignalP (v4.1)[106]. Secondary structure was predicted by PSIPRED (http://bioinf.cs.ucl.ac.uk/psipred/)[107]. Since one features of SMPs is that they contain repetitive sequences for initiating deposition of proteins and minerals, repeats within SMPs were detected with RADAR (http://www.ebi.ac.uk/Tools/pfa/radar/)[108]. For novel proteins that do not have detectable homology based on primary sequences, prediction of 3D structures and possible functions were performed by I-TASSER (Iterative Threading ASSEmbly Refinement; http://zhanglab.ccmb.med.umich.edu/I-TASSER/)[109].

Supplementary Note 2: Molecular phylogeny

2.1. Phylogenetic position of brachiopods: background

The Phylum Brachiopoda comprises of three major subphyla, Linguliformea, Craniiformea, and Rhynchonelliformea[7], the former including Lingulida and some other orders. Of these, Lingulida is the only lineage that has survived until the present, and it also has a rich fossil record dating to the Crambrian Period[53]. In spite of a great number of fossil species, our knowledge of brachiopod phylogeny is still limited. Before the 1980s, zoologists thought that brachiopods were deuterostomes based upon their mode of development (Fig. 1b-i). They were then grouped with protostomes by 18S rRNA analysis[110]. This classification was further confirmed by analyzing Hox genes in brachiopods and priapulids[111]. However, hypotheses on the evolutionary origin of brachiopods that come from paleontological[112-114] and embryological[115] studies have been highly debated. In addition, brachiopod phylogenetic position based on molecular phylogeny is still controversial (see Supplementary Fig. 6 for three types of topology; see Supplementary Table 5 for the list of full comparison). For example, whether Brachiopoda is monophyletic or paraphyletic is under debated. Analyses of small subunit (SSU) and large subunit (LSU) rRNA sequences from 12 and 21 taxa, respectively, suggest that phoronids are shell-less brachiopods, which are then grouped into Inarticulata. Phoronids and inarticulate brachiopods are combined together to form a sister group to Articulata (including brachiopods with calcium carbonate shells)[116,117]. By contrast, analysis of 7 nuclear housekeeping genes, 3 ribosomal genes, and specific microRNAs suggests that Brachiopoda is a monophyletic group and a sister group to Phoronida[7].

In recent large-scale molecular phylogenetic studies, although Brachiopoda and Phoronida are proposed as sister groups, all studies have used only one brachiopod species, which may yield unresolved results[20,21]. Therefore, it is still an open question whether Brachiopoda is a monophyletic group. Moreover, another issue needs to be addressed is the relationship between Brachiopoda and other lophotrochozoan phyla, including Phoronida, Mollusca, Annelida, and Nemertea. In addition, whether Brachiopoda and Phoronida are grouped with Ectoprocta by the so-called lophophorate hypothesis, is also debated[24,118].

The first comprehensive study addressing these issues, including 168 taxa shows that Brachiopoda (using *Terebratalia*; belonging to Rhrynchonelliformea) and Nemertea are closely related groups[1] (Supplementary Table 5). However, in that study, the interpretation of the relationship of brachiopods to other phyla may be problematic, since Mollusca became paraphyletic, which contradicts current understanding[5,6]. Further studies based on broad sampling proposed that Brachiopoda, Phoronida, and Nemertea are supraphyletic taxa called

"Kryptrochozoa"[20,21,24,119] (Supplementary Fig. 6, Type 3; Supplementary Table 5), but the bootstrap value to support this classification (lower than 70%) may not be solid enough to exclude other possibilities. Recently, large-scale transcriptome analyses including data from Platyzoa[3] and Nemertea[26] showed that the phylogenetic position of Nemertea is unstable (Supplementary Table 5). As a result, the only consistency among these studies is that brachiopods are always grouped with phoronids, which confirms the previously proposed clade Brachiozoa (i.e., Brachipoda+Phoronida)[120] (Supplementary Fig. 6). On the other hand, the relationship between Brachiozoa and Nemertea is unclear. In opposition to the idea of "Kryptrochozoa," an analysis based on 11 protein coding genes and 2 ribosomal RNA genes from 96 taxa showed that the sister group of Brachiozoa is Mollusca but not Nemertea[22] (Supplementary Fig. 6, Type 1; Supplementary Table 5). In agreement with this, analyses of SSU and LSU from 22 taxa showed similar results, suggesting Nemertea is not close to Brachiozoa[23]. In addition, a close relationship between Brachiopoda and Mollusca was supported by a large scale analysis using a 1,487 gene-matrix[2] and a broader sampling with 113 taxa[25].

Accordingly, there is still an unresolved phylogenetic issue with brachiopods. It is therefore useful to have genomic data to understand the phylogeny of lophotrochozoans. Since *Lingula* has been recognized as the most primitive group of brachiopods due to its anatomical features and life history[61], comparative studies of the *Lingula* genome with decoded genomes from three molluscs and two annelids[8-10] can provide useful information to interpret the evolution of brachiopods. Since there are no published data on genomes of Phoronida and Nemertea, the present analysis did not include these phyla for the analyses, which will be the subject of future studies on lophotrochozoan evolution.

2.2. Molecular phylogeny of *Lingula*

To identify robust phylogenetic markers, two strategies were applied. First, OrthoMCL (v2.0.9)[121,122] was used to cluster orthologous gene groups from 22 selected metazoan proteomes (Supplementary Table 1, asterisks), and then orthologs with one-to-one orthologous relationships were selected for further analyses using custom Perl scripts. Second, homology searches using a bidirectional best hits (BBH) approach[123] with BLASTP and custom Bash scripts identified the best orthologous pairs among many-to-many orthologous relationships. Alignments of orthologs were performed with MAFFT (v7.130b)[124]. Unaligned regions were then trimmed with Gblocks (v0.91b)[125] or TrimAl (v1.2rev59)[126]. Trimmed alignment blocks were concatenated with a Perl script catfasta2phyml.pl (https://github.com/nylander/catfasta2phyml). Finally, the maximum likelihood method with LG+Γ4[127] and GTR+Γ4[128] models was used to construct phylogenetic trees by RAxML (v8.0.5)[129]. Bayesian trees were constructed with PhyloBayes (v3.3f)[130] using

LG+ Γ4 and GTR+Γ4 models with the first 500 trees as a burn-in. After a run time of ~20 days (with approximately 4,000 generations), convergence of the tree topology was post-analyzed by sampling every 10 trees.

Three different phylogenetic positions of brachiopods related to other lophotrochozoans have been proposed (Supplementary Fig. 6). Since we did not include phoronids and nemerteans in our current analysis, an alternative version of the current hypotheses on the relationship among *Lingula*, molluscs, and annelids is topologically simplified (Supplementary Fig. 7a). One of the issues in phylogenetic analyses is how to select proper phylogenetic markers carrying unbiased evolutionary information (See Supplementary Note 2.4 for further analyses). In several studies using transcriptomic approaches, the orthologous relationships may be misidentified due to poor sampling of whole gene families or lineage-specific gene duplication events within specific gene families. This makes selection of target genes complicated. As the consequence, different data sets contain variations leading to different results.

To resolve these problems, we applied extensive phylogenetic analyses using genomic scale data to identify robust orthologs among selected genomes. We selected orthologs, which have only one copy in each genome, where the evolutionary pressure is supposed to be similar and the orthologous relationship is unambiguous. We selected three different marker sets including 150 one-to-one orthologs identified by OrthoMCL orthologous groups (OG) from 15 genomes (including ecdysozoans, coral, and sponge), 515 one-to-one orthologs identified by OrthoMCL OG from 10 genomes, and 2,295 ortholog pairs selected with BBH from many-to-many orthologous relationships from 10 genomes (Supplementary Fig. 7b).

Gene ontology (GO) analysis of the term biological process shows that the selected markers belong to core metabolic processes, such as ribonucleoprotein complex biogenesis and RNA processing, suggesting that they are more likely to indicate reliable evolutionary history than other highly specific genes (Supplementary Fig. 7c). The results based on 150 one-to-one orthologs from 15 genomes (with 46,845 amino-acid positions using sponges as an outgroup) indicate that *Lingula* is closely related to Mollusca rather than Annelida (Fig. 1j). Further analyses using 515 one-to-one orthologs and 2,295 orthologs found with BBH from 10 genomes (removal of sponges, corals and ecdysozoans) provided results to support this conclusion (Supplementary Fig. 7d,e). Bayesian analysis using 150 and 515 markers tested by posterior probability also yielded the same result as that of maximum likelihood (Supplementary Fig. 7f). With respect to currently available genome resources, our data confirm that Brachiopoda is closer to Mollusca, favoring the type-1 topology of the current hypothesis (Supplementary Figs 6 and 7a).

2.3. Evolutionary rate of genes associated with basic metabolism

Protein-coding genes of another “living fossil,“ the coelacanth, have been reported to be evolving significantly more slowly than those of other tetrapods[131]. Similarly, we found that a *Lingula* gene-set associated with basic metabolism (Supplementary Fig. 7c) showed the slowest evolutionary rate (i.e., the amino acid substitution rate in terms of branch length of the tree) compared to other lophotrochozoans (Fig. 1j and Supplementary Fig. 7d). This slow rate may be one of reasons why *Lingula* has retained its shell form with little modification for more than 520 million years.

2.4. Further analyses on selection of phylogenetic markers

To examine more carefully the issue of selecting proper phylogenetic markers, we further performed extensive analyses on the effects of using phylogenetic markers with different substitution rates when determining the phylogenetic relationship of Brachiopoda, Mollusca, and Annelida. We calculated the evolutionary rate of the given orthologs (“gene rate” hereafter) by summing the total branch length of the gene tree in selected genomes using a custom Perl script with a BioPerl module Bio::TreeIO. We then examined the distribution of 515 one-to-one orthologs from 10 selected genomes, and categorized their distribution into five sets (Supplementary Fig. 8a; solid red line denotes the slowest evolving genes while dashed red lines denote others with faster rates). We found that when a set of genes with slowest evolutionary rate is used, the phylogenetic position within the known chordate grouping is incorrect, suggesting only using the slowest evolving genes generate biases in phylogenetic analysis. This situation can be improved when the genes with an average evolutionary rate are added to the analysis (Supplementary Fig. 8b).

In addition, to test the effect of sampling size, we performed an analysis by sampling random marker sets of 50 genes. We showed that when the number of genes is under 100, there is an incorrect grouping of chordates, indicating that sampling size causes biases. This can be improved by sampling more than 100 genes (Supplementary Fig. 8c). Interestingly, we also found that in some cases, the phylogenetic position is unstable even using a larger sampling size. This effect was examined further by looking at the gene rate distribution of selected gene sets. On top of that, we showed that this unstable condition is due to accidental sampling of the fast-evolving genes (Supplementary Fig. 8d). To test whether fast-evolving genes contribute to the variation in interpreting phylogenetic position, we further performed bootstrap support analysis using fast-evolving gene sets (with gene rate > 6). Indeed, the unstable relationship can be observed, as in Supplementary Fig. 8e. Our analyses thus suggest that it is worth carefully examining the sample size and the gene rate of selected phylogenetic markers, since these two factors affect the final

outcome of the analysis. Taken together, these data also support the greater affinity of Brachiopoda for Mollusca than Annelida.

2.5. Lineage-specific domain loss

In addition to molecular phylogenetic analysis, we examined whether *Lingula*-Mollusca-Annelida relationship is supported by using other qualitative traits. First, annotated metazoan proteomes Swiss-Prot and TrEMBL[132] were downloaded from UniProt[101] (Supplementary Table 1). Next, we performed protein domain analysis by searching a given proteome against the Pfam database[99] using HMMER[100]. Since events of shared domain (or gene) loss mostly occurs between closely related species[133,134], we tested the relationship among genomes of *Lingula*, three molluscs (the sea snail, *Lottia gigantea*, the Pacific oyster, *Crassostrea gigas*, and the pearl oyster, *Pinctada fucata*), and two annelids (the polychaete, *Capitella teleta*, and the leech, *Helobdella robusta*) by comparing their pairwise lineage-specific domain losses.

Common domain losses were evident among the three mollusc species and between the two annelids (Supplementary Fig. 9a). Fourteen and twelve shared loses were detected between *Lingula* and *Crassostrea* and between *Lingula* and *Pinctada*, respectively. In contrast, only one and three common losses were detected between *Lingula* and *Capitella* and between *Lingula* and *Helobdella*, respectively (Supplementary Fig. 9a). These results indicate that *Lingula* is closer to molluscs but not annelids, consistent with the molecular phylogenetic analyses. In addition, during this analysis, we also noticed that the CHRD domain which is an important part of the dorsal-ventral patterning gene, *Chordin*, has been lost in the pearl oyster (*Pinctada*) and in annelids (Supplementary Fig. 9a). This finding, together with the fact that *Chordin* cannot be found in the *Helobdella* and *Capitella* genomes, but is mostly retained in other lineages dating back to cnidarians[135], supports *Chordin* loss as a synapomorphic trait in annelids, as previously suggested[136].

Furthermore, we noticed that the SOUL domain for heme-binding protein and the DAP domain for Death-associated protein are lost in annelids (See Supplementary Table 6 for a full description of 22 lineage-specific domain losses in the annelids). Functional classification on GO biological process of these 22 lost domains in annelids showed that they are mainly involved in metabolic and cellular processes (Supplementary Fig. 9b). Taken together, these suggest that annelids have specific metabolic needs and that stress responses are different from those of molluscs and brachiopods.

2.6. Microsynteny analysis

To gain further insight into the evolutionary history of lophotrochozoans[8], we conducted a microsynteny analysis of the *Lingula* genome in comparing with *Branchiostoma floridae* (amphioxus), *Lottia*, and *Capitella*. First, ortholog groups among these bilaterians were identified using OrthoMCL with proteomes downloaded from UniProt. Next, genome annotation (i.e., general feature format (GFF) file) and transcript fasta files with corresponding headers to given GFF files were retrieved (Supplementary Table 1). The relationship between each UniProt protein and the transcript was identified with BBH method. Finally, locus information for each conserved ortholog groups among selected bilaterians was acquired with custom Perl scripts. For ortholog groups with human counterparts, human IDs were used to represent the ortholog name, whereas ortholog group IDs were used for lophotrochozoan-specific genes.

We found that for lineage-specific syntenic blocks with at least 3 genes, *Lingula* shares 331, 217, and 123 with *Lottia, Branchiostoma,* and *Capitella,* respectively (Supplementary Fig. 10a). While for lineage-specific syntenic blocks with at least 5 genes, *Lingula* shares 43, 13, and 6 with *Lottia, Branchiostoma,* and *Capitella,* respectively (Supplementary Fig. 10a, numbers shown in parentheses; Supplementary Tables 7-9). Since the close distance of genes within the neighboring tightly-linked blocks (NTBs, where each gene distance is shorter than 20 kpb) may reflect the evolutionary history of selected genomes[8], we next checked these NTBs. We found one example where a cluster with three conserved orthologs (*TEX33*, *THIOM*, and *NOL11*) was retained in lophotrochozoans, but inversion of *TEX33* and *THIOM* occurred and *Branchiostoma* displays an insertion between *THIOM* and *NOL11*. The cluster in *Lingula* and *Lottia* shared an additional, flanking conserved ortholog, *SMG1*, which could not be found in *Branchiostoma* or *Capitella* (Supplementary Fig. 10b). In addition, we found a similar case in which a conserved orthologous cluster was shared by *Lingula, Lottia,* and *Branchiostoma*, but not *Capitella,* although there were genes inserted between *CTBP1* and *MAEA* in *Branchiostoma* (Supplementary Fig. 10c). Interestingly, we found many NTBs that were presented only in *Lingula* and *Lottia* but not in other genomes (Supplementary Fig. 10d,e and Supplementary Table 7). Taken together, our data strongly support that *Lingula*'s greater affinity to molluscs than to annelids, consistent with the results of molecular phylogeny and lineage specific domain loss.

Supplementary Note 3: Characterization of the *Lingula* genome

3.1. Intron structure

The average gene length in the *Lingula* genome is 6,669 bp, while transcripts average 1,425 nucleotides (Supplementary Fig. 2). The mean number of introns per gene is 6.6, with an average length of 787-bp. To better understand the evolution of intron size, we compared the genome size, gene size, and intron size of *Lingula* to those of eight decoded metazoan genomes. We found that there is a weak correlation between genome size and gene size during evolution (Supplementary Fig. 11a, R^2=0.5), but a strong positive correlation between gene size and intron size (Supplementary Fig. 11b, R^2=0.88). These suggest that during metazoan evolution, one of the main factors affecting gene size is intron size. In addition, analyses of genome size and intron size show that *Lingula* is more similar to *Lottia*, than annelids (Supplementary Fig. 11a,b). To further examine the similarity of intron structure between *Lingula* and *Lottia*, we selected 150 one-to-one orthologs used for phylogenetic analyses and analyzed intron structure among *Lingula*, *Lottia*, and *Capitella*. We found that 26 genes in all three genera contain the same number of introns, while *Lingula* and *Lottia* share 32 genes with the same number of introns. In contrast, there are only 10 genes shared with the same number of introns between *Lingula* and *Capettela* (Supplementary Fig. 11c and Supplementary Table 10). These results also support the closer relationship of *Lingula* to molluscs than to annelids.

3.2. The disorganized Hox cluster and loss of *Lox2* and *Lox4*

Hox genes, homeodomain-containing transcription factors, play an important role in regulating anteroposterior body axis and appendage development. They are highly conserved among animals, usually with a fixed gene order on the chromosome and a segmented expression pattern according to its physical location in the genome. This property is so termed “colinearity”[137]. Recent studies have shown that the Hox cluster is surprisingly conserved in bilaterians, suggesting that a single 11-gene Hox cluster is present in the last lophotrochozoan common ancestor[8].

To study the Hox cluster in *Lingula*, Hox orthologs were identified by phylogeny of the Hox gene tree (Supplementary Fig. 12a). We found *Lingula* orthologs for *Hox1*, *Hox2*, *Hox3*, *Hox4*, *Scr*, *Lox5*, *Antp*, *Post1*, and *Post2*. We failed to identify them for *Lox4* and *Lox2* in our *Lingula* gene models, despite extensive BLAST searches. When we examined this gene cluster, we found that the Hox cluster is disorganized and broken into two genomic regions. The anterior and central Hox genes lie in a 446-kb long scaffold, whereas the posterior Hox genes reside in a 413-kb long scaffold, respectively (Supplementary Fig. 12b). There are five non-Hox genes

posterior to *Lox5*, three of them with homology to known sequences (i.e., namely *EXOS6*, *ACTP1*, and *WSDU1* counted from posterior; Supplementary Fig. S12b, grey boxes). Interestingly, *Antp* was rearranged to link with *Hox1* in opposite direction (Supplementary Fig. 12b).

Fragmented Hox gene clusters have been reported in many lophotrochozoans, such as *Helobdella*, *Capitella*[8], and *Crassostrea*[9]. It may be that lophotrochozoans experienced less selective pressure to keep the intact Hox cluster due to their unique body plan. Another finding is that lophotrochozoan Hox genes *Lox4* and *Lox2* are lost in *Lingula* (Supplementary Fig. 12), although these two genes were reported in a previous study[111]. This discrepancy is possibly because Hox gene sequences obtained in the previous study were based on a PCR method. Since they were short and incomplete, this may have caused to an incompatible homology assignment.

3.3. Overall gene components

(a) Transcription factors

By Pfam domain analysis using custom Perl scripts, we examined components of transcription factor-related domains and their abundance in the *Lingula* genome, comparing them with 9 selected bilaterians. For example, the *Lingula* genome contains 37, 16, 27, 9, and 129 genes for those with bZIP, Ets, fork head, GATA, and homeobox, respectively (Supplementary Table 11). These numbers are comparable to those of molluscs, but different from those of annelids. For instance, the numbers of homeobox genes in three molluscs (*Lottia*, *Crassostrea*, and *Pinctada*) are 140, 117, and 116, respectively, and these are smaller than those of two annelids, 182 for *Capitella* and 242 for *Helobdella* (Supplementary Table 11). It is tempting to speculate that the higher number of homeobox genes in annelid lineage may be related to their segmented body plan, which is absent in the molluscs and brachiopods.

(b) Signaling pathway-related molecules

A similar Pfam domain analysis was carried out to determine components of signaling pathway-related domains. The *Lingula* genome contains 4, 5, 7, 15, and 17 genes for those with FGF, Hedgehog, Notch, TGF-beta, and Wnt, respectively (Supplementary Table 12). In general, these numbers are larger than those of molluscs and annelids, suggesting that more complicated cell-signaling-associated regulation may occur in *Lingula*.

3.4. Evolution of *Lingula* gene families

Gene families are groups of homologous genes that either originate with a speciation event (i.e., orthologs) or a duplication event (i.e., paralogs) which usually have similar functions due to their

close sequence identity[138]. The rate of gene duplication within gene families is estimated to be 17-30 genes gained and lost per million years in fruit flies and mammals[134,138]. This gain and loss of gene families has been shown to play an important role in shaping lineage specific traits[134].

To analyze gene family evolution in lophotrochozoans, we performed all-to-all BLASTP analysis followed by Markov clustering in order to identify orthologous gene groups (OG) with OrthoMCL, according to the standard protocol using a default inflation number of 1.5[121]. We then estimated gene family birth and death by computing the OG with an ultrametric tree generated by the Bayesian method using Computational Analysis of gene Family Evolution (CAFE; v3.1)[139]. The divergence times were estimated by calibrating geological time according to fossil records[25]. Non-synonymous (*Ka*) and synonymous (*Ks*) substitution rates of paired-wise paralogs were calculated with the Perl script, ParaAT (v1.0)[140], including two programs NAL2PAL (v13)[141] and KaKs_Calculator (v2.0)[142].

Important transcription factors and signaling components were annotated with Pfam domain searches using HMMER. To identify genes related to specific pathways, which are interesting topics for lineage specific evolution, the KEGG pathway database[143] was utilized. To correctly assign the orthologous relationship especially in case of many-to-many orthologs and paralogs, phylogenetic analysis on the gene tree of each gene family was conducted by maximum likelihood method with LG model[127] using PhyML (v20120412)[144] or neighbor-joining method[145] with JTT model[146] using MEGA (v6.06)[147]. Venn diagram was plotted by jvenn[148] to identify lineage specific gene families. The GO enrichment based on the gene family analyses was analyzed by DAVID (http://david.abcc.ncifcrf.gov/)[149] and PANTHER (http://www.pantherdb.org/)[150].

(a) The 20 most abundant domains in *Lingula*

The estimated gene number in *Lingula* (34,105) is larger than that of other lophotrochozoans: *Lottia* (23,800)[8], *Crassostrea* (28,027)[9], *Capitella* (32,389)[8], and *Helobdella* (23,400)[8]. This suggests expansion of genes with specific domains and/or in specific gene families. Prior to examining gene families, we first checked protein domain evolution in terms of *Lingula*-specific expansion. We compared the 20 most abundant domains in the *Lingula* genome with 9 selected bilaterians (Supplementary Table 13). We found that the top three were 576 genes with protein kinase domain, 553 genes with protein tyrosine kinase, and 504 genes with seven-transmembrane receptor (rhodopsin family) (Supplementary Table 13). In general, the number of these domains in *Lingula* is larger than in molluscs and annelids. Next five most-abundant domains in the *Lingula* genome are all related to Ankyrin repeats (Supplementary Table 13). All of the most

abundant domains are involved in cellular processes related to signaling pathways, suggesting that biological regulation in *Linugla* is more complex than in molluscs.

(b) Gene family history in *Lingula*

We analyzed the evolutionary history of *Lingula* gene families by comparing them with those of other bilaterians. Genomes of *Lingula, Branchiostoma*, *Lottia,* and *Capitella* contain 13,677, 11,056, 12,103, and 12,335 gene families, respectively (Fig. 2a). When these families were compared, *Lingula* has 3,525 unique gene families, more than *Branchiostoma* (2,341), *Lottia* (2,144), and *Capitella* (2,674). There are 2,476 *Lingula*-specific gene families without detectable homology in 22 selected metazoan genomes (Fig. 2a).

In addition, CAFE analysis showed that the turnover rate of *Lingula* gene families is the highest among selected bilaterians. The *Lingula* genome showed 7,263 gains and 8,441 losses of gene families (Fig. 2b). To better understand evolution of *Lingula* gene families, we further examined its size structure. The majority of *Lingula* gene families are small. There are ~6,000 gene families with only one copy and ~4,000 with only 2 copies (Supplementary Fig. 13a). In addition, *Lingula* has no gene families larger than 50 genes, and no highly expanded gene families were found compared with other lophotrochozoans (Supplementary Fig. 13b,c).

Furthermore, we examined the age distribution of duplicated paralogous genes by estimating their non-synonymous substitution rates (*Ks*). Among the youngest duplicated genes ($Ks < 0.1$), we found that *Lingula* genes duplicate at a rate approximately two to four times higher compared to *Lottia* (~3.8x) and *Capitella* (~2.2x) (Fig. 2c). A large portion of these young duplicated genes is undergoing negative selection, suggesting functional constraints on those genes. We also found that genes related to extracellular matrix are experiencing positive selection (Supplementary Fig. 13d,e), indicating the need to acquire new functions.

These results indicate that the *Lingula* genome has a unique evolutionary history different from other lophotrochoans. *Lingula* genes associated with basic metabolism show a slower evolutionary rate, while rapid acquisition and loss of entire gene families have occurred. These findings together with the fact that high gene duplication rate show that the *Lingula* genome has been actively evolving, contradicting the "living fossil" idea. This decoupling of the molecular and morphological evolution has been also reported in the scorpion, *Mesobuthus martensii*[151].

(c) The 20 most expanded gene families in *Lingula*

The 20 most abundant gene families in the *Lingula* genome with detectable homology and functional annotation were compared to those of 21 selected metazoan genomes (Supplementary Fig. 14a; statistical test and detailed description in Supplementary Table 14). The top five were

31 copies of chitin synthase 8 (*CHS8*), 30 of carbohydrate sulfotransferase 3 (*CHST3*), 19 of Williams-Beuren syndrome chromosomal region 27 protein (*WBS27*), 17 of helicase with zinc finger domain 2 (*HELZ2*), and 17 of cell migration-inducing and hyaluronan-binding protein (*CEMIP*) (Supplementary Table 14). Including *CHS8*, *CHST3* and *CEMIP*, five of the 20 most expanded families have possible functions in the shell formation, since their high expression level in mantle tissue (shown by asterisks in Supplementary Table 14). GO biological process analysis indicates that the expanded gene families are mainly associated with metabolic processes, localization, and cellular processes (Supplementary Fig. 14b).

CHST3 catalyzes the transfer of sulfate to chondroitin (*N*-acetylgalactosamine polymer). Chondroitin sulfate is a major component of the glycosaminoglycans (GAGs) and plays important roles in the extracellular matrix[152]. Interestingly, it has been reported that *Lingula* shells are composed of large amount of GAGs with the property mimicking an elastic isotropic gel[52]. We found that expanded *CHST3* was highly expressed in larvae and mantle tissue, which might be responsible for embryonic shell and adult shell formation, respectively (Supplementary Fig. 14c). This suggests that expansion of *CHST3* may be related to the unique elastic *Lingula* shell. In addition, *Lingula* lines the walls of its burrow with mucus secreted by the mantle[55]. We found that one of expanded gene families, mucin-4 (*MUC4*; gel-like glycosylated protein), is highly expressed in the larval stage, mantle, and lophophore (Supplementary Fig. 14d). This finding supports the secretory nature of the mantle. Furthermore, the high expression of *MUC4* genes in the lophophore also suggests that mucus may be involved in feeding and defense against pathogens.

(d) Evolution of *Lingula* chitin synthase genes

Chitin is a linear, long-chain polysaccharide of *N*-acetylglucosamine, which is the most abundant organic polymer next to cellulose and broadly used by in metazoans and fungi[153]. In ecdysozoans, it can be found mainly in body wall cuticles of crustaceans[154] and insects[155]. Also, it is the major component in mollusc shells[156], gastropod and chiton radulae[157], and cephalopod beaks[158], as well as chaetae (i.e., hair-like sensory bristles) in polychaetes[159], chitons[160], and brachiopods[161]. In addition, in many invertebrates, a chitin scaffold structure, peritrophic matrix, lines the midgut and acts as a mechanical barrier against pathogens, as well as facilitating digestion[162]. Furthermore, not restricted in protostomes, chitinous structures have been reported in the epidermal cuticle of bony fish[163]. Taken together, it is evident that chitin plays crucial roles in animals for the functions of protection, support, feeding, and digestion.

Given that chitin synthase (CHS) genes are the largest expanded gene-family in the *Lingula* genome, we performed extensive analyses of CHS gene evolution. By combining of

BLASTP, OrthoMCL, and KEGG approaches, we identified 31 CHS genes in the *Lingula* genome. First, we checked the domain combination of CHS genes, we found that most *Lingula* CHS genes carry only one CHS domain (Chitin_synth2) and others have one in combination with a Sterile alpha motif (SAM) or myosin head domain (Myosin_head) (Supplementary Table 15). Next, we identified the region of the CHS domain using HMMER (hmmscan). After that, we retrieved the amino acid sequences using custom Perl scripts. The phylogenetic tree of CHS genes was then constructed using conserved CHS domains (358 amino-acid positions). We found that there are two groups of CHS genes, which belong to the metazoan and protostome clades, respectively (Fig. 3a). The expansion of *Lingula* CHS genes can be found in both clades, in which nine *Lingula* CHS genes belong to the lophotrochozoan clade (Fig. 3a).

It has been proposed that a myosin-head-domain (MHD) might have fused to CHS genes during lophotrochozoan evolution[164]. Interestingly, we demonstrated that MHD-containing CHS genes occur only in lophotrochozoans, which is in agreement to a previous report[164]. We also found that there is a greater expansion of MHD-containing CHS genes in molluscs than *Lingula* and annelids (Fig. 3a,b). In molluscs, an MHD-containing CHS gene is expressed specifically in cells that are in close contact with the larval shell[46] and that are probably related to shell formation[165]. Its high expression level during larval shell formation and in the adult mantle further suggests the correlation with mollusc shell formation[9]. In addition, notably, the SAM domain-containing CHS genes are found only in the metazoan clade, in which amphioxus CHS genes are highly expanded. The combination of SAM and CHS domains has been reported in amphioxus[166] and can be also found in corals and sponges (Fig. 3a), suggesting that this combination is likely an ancient character dating back to the metazoan ancestor.

Since *Lingula*'s chitinous structures, such as shells[52], chaetae[161], and pedicles[167] are well known, we further examined the expression pattern of these CHS genes. Transcriptome analysis of *Lingula* CHS genes shows that they are expressed in all adult tissues and in the larval stage (Fig. 3c). The MHD-containing CHS gene is highly expressed in the larval stage and mantle, suggesting that it may also play a role in *Lingula* shell formation (Fig. 3c). Additionally, CHS genes are highly expressed in the gut and digestive cecum, suggesting that a chitinous peritrophic matrix may also be present in the *Lingula* midgut (Fig. 3c). The expansion of CHS genes in the *Lingula* genome and different expression profiles of these genes suggest that chitin plays significant roles in biomineralization and digestion, which should be carefully examined in the future studies.

3.5. Repetitive elements

Repetitive sequences in the genome were identified with RepeatScout (v1.0.5) using the default settings (i.e., sequence length larger than 50 bp and occurring over 10 times). Repeats were annotated with a TBLASTX search against Repbase (v20130422). We found 6,926 repetitive elements, far more than in three other lophotrochozoan genomes (*Lottia*, 2,891; *Capitella*, 5,220; and *Helobdella*, 1,901). On the other hand, 22% of the *Lingula* genome consists of repeats, which is similar to *Lottia* (~21%), but lower than *Capitella* (~31%) and *Helobdella* (~33%)[8]. Only 528 of these repeats have been annotated, which represents just ~7% of the *Lingula* repetitive sequences.

The most abundant DNA transposon, long terminal repeat (LTR) retrotransposon, and Non-LTR retrotransposon are Tc1/mariner-like (TcMar) (2.3%), Gypsy (0.2%), and RTE (0.7%), respectively (Supplementary Table 16). In three other lophotrochozoan genomes, the most abundant DNA transposon is variable (*Lottia*, Maverick; *Capitella*, TcMar; and *Helobdella*, hAT), while the major retrotransposon in all of them is Gypsy[8]. Further analyses of these unknown repeats (e.g., detailed annotation and genomic distribution) in the *Lingula* genome will illuminate lophotrochozoan evolution.

Supplementary Note 4: Evolution of *Lingula* biomineralization

4.1. Biomineralization in shell and bone formation: background

From bacteria to vertebrates, biomineralization is employed to make hard tissues, mostly in the form of calcified minerals with carbonate or phosphate, for protection, support, and feeding[168-170]. Molluscs may be the most successful animal group that forms hard external tissues. Like most other marine invertebrates, mollusc shells are composed of calcium carbonate (i.e., $CaCO_3$) (Supplementary Table 17). The mineral parts constitute more than 90% of the shell weight, and the mass of organic matrix in the shell is usually less than 5%[171,172]. Most mollusc shells have three major layers. The outermost layer, the periostracum, is composed of chitin and organic matrix. The middle, or prismatic layer, is a thin sheet composed of crystalline calcite and aragonite, and the inner layer, the nacreous or foliated layer, is the thickest, and is composed of crystalline aragonite[172,173]. In contrast, *Lingula* shells are rich in organic materials which represent about 40% by dry weight[174], and are made of calcium phosphate[175] in the form of carbonate-substituted fluorapatite (i.e., $Ca_{10}(PO_4)_6F_2$, or francolite) (Supplementary Table 17). Similar to mollusc shells, brachiopod shells also consist of three major layers. The outermost layer, periostracum (~4 µm), is an organic layer composed of chitin and organic matrix. The primary layer (~40 µm) is composed of rod and botryoid types of apatite and glucosaminoglycan gels (GAGs; with long unbranched polysaccharides). The secondary layer, the laminated layer (variable in thickness), is composed of apatitic laminae[52,174]. The laminated structure provides flexibility and fracture resistance, which may benefit burrowing[176]. It is worth mentioning that in *Lingula* there are collagen fibers at the interface of the primary and secondary layers, a feature not shared by molluscs[52,174,177] (Supplementary Table 17).

Biomineralization has been extensively studied but the molecular mechanism remains unknown. The process has been termed as "biologically induced" or "biologically controlled" depending on the degree of biological control involved. The minerals are formed by biologically induced processes if their precipitation is the result of interactions between the organism and the environment, in which cell surfaces and compartmentalized fluid cavities catalyze nucleation and growth of the minerals (i.e., mineralization is initiated by an extracellular organic matrix). On the other hand, the biologically controlled process involves direct control of nucleation, growth, morphology, and location of mineral deposition via intracellular regulation[178]. In humans, for example, cells capable for making calcified tissues, such as cartilage, bone, and dentin, form so-called matrix vesicles, that bud off from specific regions of the plasma membrane and regulate ion concentration and mineral formation intra-cellularly and intra-vesicularly[179,180]. In sea urchins, larval endoskeletons or spicules are formed intra-cellularly in membrane-delineated

compartments generated by multiple skeletogenic cells[181]. Skeletogenic cells are able to transform minerals from amorphous calcium carbonate into crystalline calcite[182,183].

Two models have been proposed for the mechanism of mollusc shell formation. The matrix-secreted model (i.e., biologically induced) suggests that the mantle epithelial cells secrete shell matrix proteins and ions into a compartment (i.e., extrapallial space) where the minerals are formed[171,184], whereas various tissues may also contribute to this secretion process[185]. In the cell-mediated model (i.e., biologically controlled), cells (e.g., granulocytic hemocytes in case of oysters) form the minerals intra-cellularly, in which crystal nucleation is initiated under cellular regulation[9,186]. Taken together, it is reasonable to hypothesize that these two models might both be involved in the biomineralization during shell formation.

Even though there is a lot of interest in mollusc shell formation, the evolutionary origin of mollusc shells is unclear. Studies of mollusc mantle transcriptomes and shell proteomes suggest that gene sets responsible for formation of calcium-carbonate-based calcite or aragonite are evolved rapidly. Mineral homology among molluscs might be the result of parallel evolution, since their "toolkit" genes of many species are so diverse[187-189]. Supporting this view, new shell matrix proteins may have originated from gene duplication events, in which those genes were initially responsible for general functions and were later co-opted for calcification[190]. One interesting proposition is that horizontal gene transfer from bacteria may also have contributed to the rapid neofunctionalization of biomineralization gene sets during early metazoan evolution[191,192], although this idea is still a matter of debate.

In contrast to studies of mollusc shell formation, the origin of the *Lingula* shell is largely unknown. Although some Cambrian arthropods, tommottids, and various other problematica also used calcium phosphate for their skeletons[193], one intriguing observation is that lingulid brachiopods and craniates (i.e., head vertebrates) are the only two well-characterized groups of extant animals that utilize calcium phosphate minerals[168]. Given that vertebrate bones are made up of hydroxyapatites (i.e., $Ca_{10}(PO_4)_6(OH)_2$), fibrillar collagens, and GAGs[194], which are similar in composition to *Lingula* shell[195] (Supplementary Table 17), it is tempting to wonder whether the mechanism of biomineralization between these distant phyla shares a common origin.

However, using solid-state nuclear magnetic resonance spectroscopy and X-ray diffraction, a recent study found that *Lingula* shell has higher mineral crystallinity and shows no GAG-mineral interaction compared to vertebrate bone[196]. Comparison of ultrastructure by electron diffraction confirmed the higher crystallinity and also determined that carbonate content is lower, in contrast to vertebrate bone[197]. These findings cast doubt on the idea that *Lingula* shell and vertebrate bones involve the same gene sets. Genomic scale comparisons of

biomineralization genes among *Lingula*, molluscs, and vertebrates may provide interesting insights into the molecular mechanism and evolutionary origin of the *Lingula* shell.

4.2. Properties of *Lingula* mantle revealed by transcriptome analysis

To characterize genes that might be involved in *Lingula* shell formation, seven adult tissues were collected for RNA-seq (Supplementary Table 4). Transcript expression level was calculated as FPKM using Trinity built-in scripts with RSEM[94,96]. A Venn diagram was plotted using jvenn to identify mantle-specific genes. GO enrichment analysis, such as molecular functions and biological processes of mantle-specific genes, was conducted with DAVID[149] and PANTHER[150].

Given that mantle epithelium is the place where shell formation occurs, genes that are specifically or highly expressed in the mantle may participate in biomineralization. We found that among five adult tissues, there are 2,724 genes specifically expressed in the mantle (Supplementary Fig. 15a). GO enrichment analysis showed that these genes are responsible for cell surface receptor signaling and cell adhesion. They encode extracellular or integral membrane proteins, such as G-protein receptors (Supplementary Fig. 15b,c and Supplementary Table 18). Notably, they contain domain features like EGF, sulfotransferase, neuropeptide binding, and others. (Supplementary Table 18). These data indicate that the *Lingula* mantle is an actively secreting organ that expresses specific sets of glycoproteins and extracellular matrix proteins. This is similar to a previous report showing that 25% of mollusc mantle genes encode secreted proteins[187]. Our results also support the proposal that the appearance of calcified tissues at the Precambrian-Cambrian transition might have originated from reorganization of preexisting secretory machinery[198]. In addition, we found genes related to respiratory gaseous exchange enriched in the mantle, which might relate to the mantle canal, a unique circulation organ in brachiopods[199] (Supplementary Fig. 15b).

Besides searching for genes that are specifically expressed in the mantle, we also analyzed genes that are more highly expressed in the mantle than in other tissues. We found that collagen and zonadhesin are the two mostly highly expressed genes in the mantle (Supplementary Table 19). In addition, many calcium ion-binding proteins are highly expressed in the mantle, such as calmodulin, calponin, EGF domain-containing protein, and uromodulin (Supplementary Table 19, daggers). In mice, calponin is a negative regulator of bone formation. Calponin knockout mice increase bone formation by enhancing responsiveness to BMP signaling[200]. Interestingly, calponin is highly expressed in the pearl oyster mantle[35] and the pearl sac[201], suggesting that it plays a role in calcification. Furthermore, we noticed that one mucin gene is highly and specifically expressed in the mantle (Supplementary Table 19). Mucin genes have

been found in coral skeleton[202,203] and in mussel shell[49], suggesting that they play a conserved and ancient role in hard tissue formation in metazoans.

4.3. Tissue transcriptomic comparison between *Lingula* and *Crassostrea*

(a) Spearman's and Pearson's correlation coefficients

Given the close evolutionary history between *Lingula* and molluscs, we next examined whether *Lingula* tissues also share molecular similarity in transcriptomes with those of molluscs. RNA-seq raw reads of selected adult tissues from the Pacific oyster *Crassostrea gigas*, which are comparable to those of *Lingula*, were downloaded from OysterDB (http://oysterdb.cn/)[9] and reassembled with Trinity[94]. Orthologous genes were identified using a BBH approach[123]. To identify the transcriptome similarities between *Lingula* and *Crassostrea* tissues, we assessed the strength of the linear relationship of orthologous gene expression levels using both Spearman's rank correlation coefficient (ρ) and Pearson's product moment correlation coefficient (r).

Spearman's ρ is robust when the data set contains extreme values, while Pearson's r is affected by outliers[204]. Both coefficients were calculated using custom Bash and Perl scripts. We first calculated Spearman's coefficient (ρ). The defined value of the coefficient (ρ) is

$$\rho = 1 - \frac{6\sum d_i^2}{n(n^2-1)}$$

where $d_i = x_i - y_i$ is the difference between the two rank values, and n is the sample size (i.e., the number of BBH orthologs; 6,315 orthologs were identified). In brief, a serial number was given to each orthologous pair. Orthologs were then sorted and ranked by expression level. Afterward, a global comparison was performed.

We further conducted an analysis using Pearson's coefficient (r). The value of the coefficient (r) is defined by

$$r = \frac{\sum_{i=1}^{n}(x_i - \mu_x)(y_i - \mu_y)}{\sigma_x \sigma_y}$$

where between transcriptomes x and y, there are n orthologous pairs, x_i and y_i are the expression levels in FPKM, μ_x and μ_y are the average FPKM values of each transcriptome, and σ_x and σ_y are the corresponding standard deviations. To compare two transcriptomes differ in gene expression by orders of magnitude, we performed a log transformation of FPKM for Pearson's r. When comparing Spearman's ρ and Pearson's r, we found similar trends of the correlation

pattern, indicating that it is appropriate to use either of these coefficients for our analyses (Supplementary Fig. 16).

(b) Transcriptome similarities between *Lingula* and *Crassostrea*

When compared intra-specifically, we found that the *Lingula* mantle transcriptome is most similar to those of lophophore and pedicle, while the *Crassostrea* mantle transcriptome most resembles those of labial palp and gill (Supplementary Fig. 17a,b). Interspecific comparisons showed that *Lingula* mantle is related to *Crassostrea* mantle (Fig. 4a and Supplementary Fig. 17c,d; MT vs Man), suggesting shared functional similarity in *Lingula* and mollusc mantles. In addition, our analysis showed *Lingula* mantle and *Crassostrea* gill are highly similar (Fig. 4a and Supplementary Fig. 17c,d; MT vs Gil). This is likely because the *Lingula* mantle canal is used for gas exchange[199], functioning like mollusc gill. On the other hand, *Crassostrea* mantle also shared similarity with *Lingula* pedicle, which may be explained by the fact that both mantle and pedicle are actively secreting organs (Fig. 4a and Supplementary Fig. 17c,d; Man vs PC). Indeed, it has been proposed that there are similarities in secretory activity of epithelium between the pedicle and mantle, based on transmission electron microscopy[205]. Our molecular evidence supports this notion. Furthermore, *Lingula* pedicle also shares similarity with *Crassostrea* adductor muscle, which may reflect the muscular nature of the pedicle[205,206] (Fig. 4a and Supplementary Fig. 17c,d; PC vs Amu). Interestingly, our analysis revealed that *Lingula* lophophore shares high similarity with *Crassostrea* gill (Fig. 4a and Supplementary Fig. 17c,d; LP vs Gil). This is likely due to the lophophore's role in collection of food and gas exchange[207].

To further explore the functional similarity of mantles, we categorized each orthologous gene pair by calculating their percent difference (PD), in which two values (x_i and y_i) are compared at log scale in the following manner:

$$PD = \frac{\sqrt{(x_i - y_i)^2}}{(x_i + y_i)/2} \times 100\%$$

By applying GO enrichment analyses to different PD subsets, we found that the expression profiles of genes involved in ribosomal machinery are most similar, while those of genes related to chromosome and cell cycle regulation are diverse (Supplementary Fig. 18). Genes related to membrane trafficking are expressed in a highly similar pattern between *Lingula* and *Crassostrea* mantles, suggesting that the functional similarity mainly comes from genes involved in secretory machinery (Supplementary Fig. 18a,c).

4.4. Comparative genomics of genes associated with biomineralization

We next examined the known biomineralization-associated genes in the *Lingula* genome. Using recent published resources on bone evolution in the elephant shark, *Callorhinchus milii*[208], shell formation in the Pacific oyster, *Crassostrea*[9], and silk genes in the spiders, *Stegodyphus mimosarum,* and *Acanthoscurria geniculata*[209], we conducted comparative analyses on biomineralization genes associated with bone, shell, and silk formation. A full list of genes involved in biomineralization was acquired from supplementary information published with genome papers. The BBH approach was used to identify orthologous relationships. We then compared these genes on a genomic scale using humans (*Homo*), sharks (*Callorhinchus*), *Lingula*, and molluscs (pearl oyster, *Pinctada*, Pacific oyster, *Crassostrea*, and sea snail, *Lottia*) genomes. The heatmap and clustered matrix were created using R (v3.0.2; http:/www.R-project.org/)[210] with the package Bioconductor (v3.0)[211] and pheatmap (v0.7.7)[212].

(a) Genes associated with vertebrate bone formation

A recent study of the elephant shark genome reveals that innovation of acidic secretory calcium-binding phosphoprotein (SCPP) gene family holds the key to vertebrate bone formation[208]. It has been proposed that *SCPPs* arose from a duplication of gene for secreted protein, acidic, cysteine-rich like 1 (*SPARCL1*) after the divergence of cartilaginous and bony fishes[213]. Therefore, we first examined whether *Lingula* has *SCPPs* or not.

A Pfam domain search for SPARC calcium binding domain (SPARC_Ca_bdg) revealed that there are 139 SPARC_Ca_bdg domain-containing genes in the *Lingula* genome, a number that is comparable to those of other lophotrochozoans, but higher than vertebrates (Supplementary Fig. 19a). Further examination of SPARC-related genes revealed a combination of SPARC_Ca_bdg and Kazal domains. Taking account of this combination, there are only two SPARC-related genes in *Lingula* (Supplementary Fig. 19b). Domain composition analysis showed that SPARC genes do not contain a Thyroglobulin_1 domain, which is typical of other SPARC-related families (Supplementary Fig. 19c). Phylogenetic analysis of *Lingula* SPARC-related genes demonstrated that *Lingula* has only one SPARC gene, and the other one is an ortholog of SPARC-related modular calcium-binding protein (*SMOC1/2*) (Supplementary Fig. 19d). This finding suggests that *Lingula* does not have *SCPPs* that arose from *SPARCL1*.

Next, we examined 175 vertebrate bone formation genes in selected metazoan genomes. We found that many genes involved in vertebrate bone formation are derived from genome duplication events in the vertebrate lineage. For most of these genes, *Lingula* shares similar number of homologs to other marine invertebrates; there is no unusual similarity between *Lingula* and humans (Fig. 4b, Vertebrate bone formation; Supplementary Tables 20-22). Transcriptome

analysis of bone formation genes further demonstrates that most of these genes are expressed ubiquitously during embryogenesis and in all adult tissues, suggesting that they have multiple roles, not just biomineralization (Supplementary Tables 21 and 22). Consistent with the SPARC analysis, we failed to find the key bone formation genes *SCPPs* in the *Lingula* genome. Taken together, our data suggest that *Lingula* and bony vertebrates independently evolved their own mechanisms for hard tissue formation, as did sea urchins[214].

(b) Genes associated with mollusc shell formation

On the other hand, a comparative study of 90 mollusc shell formation-associated genes showed that *Lingula* shares most of the common "toolkits" with sea snail and oysters, but there are also many oyster-specific genes that cannot be found in other bilaterians (Fig. 4b, Mollusc shell formation-related proteins). Further analysis of these genes revealed that many so-called shell formation genes are also shared with humans (Supplementary Fig. 20a). GO functional classification showed that these 30 core-shared genes are mainly related to cellular and metabolic process, localization, and biological regulation (Supplementary Fig. 20b). In addition, transcriptome analysis in *Lingula* adult tissues demonstrates that expression of these shared genes is not limited to the mantle and many of them are not expressed. These results suggest that many shell formation genes have been co-opted for mollusc shell formation independently, while they carry out different functions in other bilaterian lineages (Supplementary Table 23). Notably, there are eight genes shared between *Lingula* and molluscs; five of them exhibited high expression in larvae and the mantle. These include genes for calcium-dependent protein kinase[35], chitin synthase[46], extrapallial (EP) protein precursor[215], PFMG8[216], putative uncharacterized protein F18, tyrosinase[217], and veliger mantle 1[218] (Supplementary Fig. 20a; Supplementary Table 24, LOCP). These genes may also be involved in *Lingula* shell formation.

(c) Genes associated with spider silk formation

Lastly, we could not detect any spidroin-like protein genes in the genomes that we compared. When searching for silk proteins in the *Lingula* genome, no homolog with sequence similarity was found (Supplementary Table 20, Spider silk proteins). This suggests that silk formation is unlike that of *Lingula* shell, although there are proteins with alanine-rich regions shared in shell matrix[219] and silk proteins[220]. Given that alanine-rich proteins are the main constituents of the *Lingula* shell[177,221], it is possible that *Lingula* evolved poly(alanine) silk-like proteins independently to develop shell extensibility, which may play an important role in their burrowing lifestyle.

4.5. Conserved molecular mechanisms in metazoan biomineralization

Although the *Lingula* mantle is similar to that of molluscs, we cannot exclude the possibility that the similarities might represent nothing more than the sharing of common secretory cell types. The question remains whether conserved molecules and mechanisms exist for shell formation. To resolve this issue, we focused on one of the ancient metazoan signaling pathways, bone morphogenetic proteins (BMP; or Decapentaplegic, Dpp). BMPs are signaling ligands belonging to the transforming growth factor-β (TGF-β) superfamily. The BMP pathway has been conserved for dorsal-ventral patterning in bilaterians[222] and for symmetry breaking in cnidarians[223-225]. These results suggest that it has an ancient role in regulating the body plan. Intriguingly, BMP signals are also required for bone formation in vertebrates[28], shell formation in molluscs[226] and skeleton formation in corals[227].

To explore the possible role of BMP signaling during embryogenesis, we first annotated BMP ligands and receptor-regulated Smad. *Lingula* has orthologs for one *Bmp2/4*, one *Bmp5-8*, and one *Smad/1/5/9* (Supplementary Fig. 21a,b). Our embryonic transcriptome showed that *Bmp5-8* and *Smad1/5/9* are expressed maternally, while *Bmp2/4* is expressed after the early blastula stage (Supplementary Fig. 21c). Given the conserved role of BMP signaling during early development, the functional motifs of Smad1/5/9 sequences are highly conserved. Taking advantage of that, immunostaining with a commercial phosphorylated Smad1/5/9 antibody (i.e., phospho-Smad1 (Ser463/465)/ Smad5 (Ser463/465)/ Smad9 (Ser465/467) antibody; Cell Signaling 9511) has been shown to specifically detect activation of canonical BMP signaling and has been widely used in marine invertebrates, such as amphioxus[228], sea urchins[229], hemichordates[230], and sea anemones[231] (Supplementary Fig. 21b,d).

To visualize activation sites of BMP signals, we applied immunostaining of nuclear phosphorylated Smad1/5/9 (pSmad), an activated mediator for the signaling[229]. The expression profile of *Bmp2/4* is coincident with the nuclear pSmad signals, suggesting that activation of BMP signaling requires *Bmp2/4* expression (Supplementary Fig. 21c). The commercial pSmad antibody is produced from a synthetic phosphopeptide corresponding to residues surrounding Ser463/465 of human SMAD5, and cross-reacts with human SMAD1 and SMAD9. To validate the specificity of pSmad staining in *Lingula*, we compared C-terminal sequences of Smad proteins in selected metazoans. The alignment shows that C-terminus of *Lingula* Smad1/5/9 is identical to human SMAD1 and SMAD9 (Supplementary Fig. 21d). This observation suggests that the commercial pSmad antibody may also be useful in lophotrochozoans (Supplementary Fig. 21d). Furthermore, we observed that nuclear pSmad signals start to appear at the early blastula stage (Supplementary Fig. 21c). The signals are strongest at the early gastrula stage showing an asymmetrical pattern. This indicates that BMP signaling may play a role in axial

patterning in *Lingula* (Supplementary Fig. 21e). Thus, the temporal correspondence between staining signals and *Bmp2/4* expression, as well as their nuclear localization and asymmetrical pattern in the embryo, argue strongly against the possibility of non-specific binding. More detailed studies will be required to address the function of BMP signaling during *Lingula* embryogenesis.

In *Lingula*, embryonic shells are formed upon mantle lobes starting at the 1-pair-cirri larval stage[54]. Interestingly, at different larval stages, we found that BMP signals are activated at the anterior margin of the mantle lobe, suggesting that the signal may be involved in embryonic shell formation (Fig. 5 and Supplementary Fig. 21f, arrows). In gastropods, *Bmp2/4* is expressed in posterodorsal ectoderm along the mantle edge[27,31,232]. On the other hand, in bivalves, *Bmp2/4* is expressed in the shell field and shell field invagination[233]. Given that BMP signals are activated at the margin of *Lingula* mantle lobes, these findings suggest that BMP signaling may play a conserved role in biomineral formation in the metazoan common ancestor. Further analyses of how BMP signaling regulates embryonic shell formation in *Lingula* will be informative to understand the evolution of biomineralization.

4.6. The *Lingula* shell matrix proteome

(a) Identification and characterization of *Lingula* shell matrix proteins (SMPs)

Proteomic approaches have recently been introduced into the field of mollusc biomineralization, where they provide powerful tools to identify novel shell matrix proteins (SMPs)[47,234-236]. The mantle epithelium has multiple functions. In addition to shell formation, it is also responsible for mucus secretion, light sensing, and circulation. To identify *Lingula* SMPs that are possibly directly involved in shell formation, we conducted proteomic analysis of the matrix proteins from the *Lingula* shell (Supplementary Fig. 22a). We found a total of 231 putative SMPs by retrieving gene models with high-quality peptide hit(s). To avoid contamination from other tissues or cells, we identified SMPs by applying the following strategy.

First, we classified putative SMPs by their solubility and found that most of them were in the acid insoluble fraction (Supplementary Fig. 22b; 146 acid insoluble proteins, 46 acid soluble proteins). Next, we found that most of putative SMPs had only one unique peptide hit (Supplementary Fig. 22c) and many of them lack signal peptides (Supplementary Fig. 22d). Using a GO statistical overrepresentation test, we showed that selection of putative SMPs with unique peptide hits (>1) and with signal peptides significantly enriched proteins that are related to extracellular matrix (Supplementary Fig. 22e). In addition, it has been reported that tandem duplication often occurs in genes related to biomineralization[194]. To select the final set of SMPs, we then applied the combination of genes with unique peptide hits (>1), with signal peptides, and

those showing tandem duplication of the scaffold (Supplementary Fig. 22f). Finally, we identified 65 SMPs in the *Lingula* shell proteome, 51 of which are present in all metazoans, and 14 are *Lingula*-specific, without counterparts in any other organism.

Characteristics of these SMPs such as domain composition, pI, and percentages of amino acid are given in Supplementary Tables 25 and 26 for those with homologies and for novel ones, respectively. Unexpectedly, we could not find secreted acidic proteins (pI<4.5)[237] among *Lingula* SMPs. Instead, many novel SMPs were basic (Supplementary Table 26). Further analysis of these 65 SMPs showed the following features: 14 had no detectable homology (i.e., novel), 20 lacked functional annotation, and 31 had functional annotation. Functional classification analysis of the 31 SMPs showed that they are mainly related to extracellular matrix proteins, receptors, cell adhesion molecules, and hydrolases (Supplementary Fig. 23a).

Through an examination of amino acid composition, one of the main characteristics of *Lingula* shells compared with other articulate brachiopods or molluscs is that their SMPs contain a large amount of glycine and alanine[52,177,221]. To support previous observations, we provided the first molecular evidence to show that glycine-rich SMPs are collagens (Supplementary Table 25, G%>20). In addition, we also found that many novel SMPs are alanine-rich and in low molecular weight (~10-20 kDa, amino-acid length ~100-200) (Supplementary Fig. 23b and Supplementary Table 26). Pfam analysis of *Lingula* SMPs shows that the most abundant domains are cadherin, collagen, and thrombospondins 1 (TSP_1), whereas the most abundant proteins contain von Willebrand factor type A (VWA), epidermal growth factor (EGF), and TSP_1 domains (Supplementary Fig. 23c and Supplementary Table 27). The domain composition suggests that the shell matrix is derived from extracellular matrix[238].

We next examined the expression profile of these SMPs. We found that 26 SMPs are expressed ubiquitously in all adult tissues, indicating that they have functions other than shell formation (Supplementary Fig. 24a). On the other hand, 20 SMPs exhibited specific expression in the mantle. These include collagen (*CO4A2*), chitinase (*CHIT3*), glutathione peroxidase (*GPX3*), hephaestin (*HEPH*), hemicentin (*HMCN1*), peroxidasin (*PXDN*), von Willebrand factor A domain-containing protein (*VWA1*), and fibrillin (*FBN2*) (Supplementary Fig. 24b and Supplementary Table 28). Many of these genes function as extracellular enzymes and ion binding sites in humans, suggesting that they are probably co-opted in *Lingula* for shell formation. Their expression in both the mantle and the shell implies that they may be directly involved in biomineralization. We also showed that five SMPs are weakly expressed or have no expression in the mantle, suggesting that they have been deposited into the shell matrix in the earlier event of the production (Supplementary Fig. 24c). All 14 *Lingula*-specific SMPs are highly or specifically expressed in the mantle, indicating specific roles in shell formation (Supplementary Fig. 24d).

Taken together, nearly one third of SMPs are expressed ubiquitously, while half of them are expressed specifically in the mantle (Supplementary Fig. 24e).

(b) Comparative genomics of *Lingula* SMPs

When *Lingula* SMPs are compared to those of other bilaterians, we found that most of the *Lingula* shell proteins are highly specific, and are not present in either molluscs or vertebrates (Fig. 4b, *Lingula* shell matrix proteins). To gain insights into the evolutionary origins of mineral formation genes, we excluded SMPs that are present only in the *Lingula* lineage (i.e., novel) or shared by all other animals. After filtering, we identified 29 SMPs, which were further analyzed by comparing them with those found in 12 selected metazoan genomes. By comparative genomics, we found that the composition of *Lingula* SMPs shared homology mostly with those of amphioxus and molluscs (Supplementary Fig. 25). These data are consistent with those of the whole genome comparison with bone formation genes (Fig. 4b, Vertebrate bone formation).

Regarding the phylogenetic debates on the relationship of brachiopods, molluscs, and annelids (Supplementary Fig. 6), we searched for SMPs that are only shared by *Lingula* and annelids; however, we found none. Instead, we discovered 11 SMPs that were lost in the annelid lineage, but that have been retained in the other lineages. Taken together, analyses of the subset of SMPs indicate a close relationship between *Lingula* and molluscs, suggesting that some of the SMPs already existed before the common ancestor of *Lingula* and molluscs.

(c) Novel *Lingula* SMPs

Recent proteomic studies of molluscan shells have shown that both highly conserved and lineage-specific genes are expressed in the shell matrix[47,234], suggesting that each mollusc lineage may use different genes for shell formation, according to environmental conditions and genetic context. One important finding of our shell proteome study is that *Lingula* carries a lot of lineage-specific SMPs. Careful examination revealed that some of these SMP genes have tandem duplicated architecture in the genome. One example is an alanine-rich gene family that has three copies arranged in tandem on the same scaffold (Supplementary Fig. 26a). These novel secreted alanine-rich proteins contain conserved 4-5 poly(alanine) blocks and GYGY motifs (Supplementary Fig. 26b).

Poly-alanine proteins are usually found in silk proteins with poly(glycine-alanine) or poly(alanine) motifs[239]. It is proposed that the repetitive poly(alanine) motifs in the silk protein are able to fold into β-sheet, forming highly oriented alanine-rich crystals[220]. Intriguingly, similar alanine-rich SMPs have also been found in oysters. But in comparison with the 4-8 poly(alanine) blocks in silk proteins, oyster SMP, MSI60, has 9-13 poly(alanine) blocks, which may contribute

to pack crystals more densely[219]. Another oyster SMP, Shelk2, has 7-8 poly(alanine) blocks. This protein is expressed in the fresh shell framework structure prior to shell regeneration[240]. *Lingula* alanine-rich SMPs have 4-6 poly(alanine) blocks, which are more similar to those of silk proteins than of oysters. To gain more insight into the function of these novel proteins, we predicted their 3D structure with I-TASSER[109]. Interestingly, we found that the top-scoring predicted structure is similar to that of a recently designed artificial monomeric three-helix bundle (Supplementary Fig. 26c; C-score=-2.72), which has high thermodynamic stability[241]. It is likely that properties of this novel helix protein contribute to the unique features of the *Lingula* shell. Further studies on this protein will be needed to elucidate its role in shell formation.

4.7. Evolution of *Lingula* fibrillar collagen

(a) Phylogeny of fibrillar collagens

Bone formation in vertebrates relies on depositing apatite crystals on fibrillar collagens[242]. Under scanning electron microscopy, *Lingula* shells show collagenous fibrils associated with GAGs[52]. Collagen fibers are not detected in the shell proteome of the Pacific oyster, suggesting that mollusc shells are not composed of fibrillar collagen[9]. Given that biominerals with fibrillar collagens are one of the characteristics shared by *Lingula* and vertebrates, using phylogenetic analysis on the evolution of fibrillar collagen, we tested whether they shares a common origin of biomineralization with vertebrates.

Vertebrate fibrillar collagens can be grouped into three major groups (Clade A, B, and C) carrying COLFI domains[194] (Fig. 6a). Our analysis shows that the fibrillar collagens used for shell formation in *Lingula* do not have COLFI domains. Instead, they comprise a new group of collagens with EGF-like domains, which do not belong to the vertebrate type of fibrillar collagens (Fig. 6a,b). These new types of collagen are expressed in the shells and mantle, suggesting their direct involvement in shell formation (Fig. 6a and Supplementary Table 28). This finding is consistent with the previous observation that the ultrastructure of *Lingula* shell collagen fibers is different from that of vertebrates[174,177]. Notably, some fibrillar collagen genes likely arose by tandem duplication (Fig. 6c).

(b) Shuffling of EGF and Collagen domains

It has been shown that domain shuffling contributes to the evolution of lineage-specific characteristics in vertebrates[243], fruit flies[244] and corals[202]. Given that *Lingula* collagens carry a new domain combination, which is not found in vertebrate-type fibrillar collagens, we analyzed the domain shuffling based on EGF and collagen domains. Supplementary Tables 29 and 30

summarize the most abundant domains combined with EGF and collagen domains, respectively, in *Lingula*, humans, and molluscs (sea snail, Pacific oyster, and pearl oyster). We found that *Lingula* contains 17 genes encoding proteins with combination of EGF and collagen domains (Supplementary Table 29), the number of which is the highest among bilaterians (Supplementary Fig. 27a). Four of these 17 EGF domain-containing collagens are found in the shell matrix proteome (Fig. 6b). Further analyses of domain combinations showed that *Lingula* carried higher number of EGF domain-containing proteins and these with domains combined with EGF domain in others bilaterians (Supplementary Fig. 27b). On the other hand, the number of collagen domain-containing proteins in *Lingula* is higher than in molluscs but similar to annelids (Supplementary Fig. 27c). In addition, we found that 11 of the 20 most abundant domains combined with EGF domains are commonly shared by other bilaterians, whereas a collagen domain is specially linked to EGF domains in *Lingula* (Supplementary Fig. 27d). These results suggest that EGF-domain shuffling occurred more frequently in the *Lingula* lineage and contribute to generate new types of collagens with a novel domain combination.

Taken together, our genomic and proteomic analyses suggest that the characteristics of biomineralization shared by *Lingula* and vertebrates probably arose through independent evolution. Indeed, many examples of parallel evolution have been shown. For example, studies on collagen evolution among vertebrates and basal chordates show that three different fibrillar collagen clades mentioned above occurred independently, a co-option in which collagen was used for biomineral formation of chordates[245]. Similarly, studies of biomineralization genes in sea urchins and molluscs (bivalves and gastropods) show that there are extensive differences in their expressed gene sets. These are usually lineage-specific, suggesting that biomineral proteins arose independently various times in metazoans[188,189,214].

4.8. Evolution of bilaterian biomineralization

(a) Biomineralization mechanisms in *Lingula*

We have demonstrated that *Lingula* used its own gene sets to originate their calcium phosphate chemistry that is different from the set used by vertebrates. In addition, we have shown that there are lineage-specific SMPs in *Lingula* and molluscs, respectively. A schematic summary of genes involved in *Lingula* shell formation identified by this study is given in Fig. 7. References supporting the illustration are provided in Supplementary Table 31. We proposed that the metazoan ancestor used a core of ancient signaling proteins to initiate the biomineralization process. We speculated that this involves canonical BMP signaling, in which BMP ligands bind to its receptor, from which a signal is transduced by the regulatory and co-mediator, pSmad1/5/9 and Smad4, respectively. They then act as transcription factors, interacting with other proteins to

activate the expression of downstream biomineralization genes (Fig. 7, proteins in green). The other conserved transcription factor is engrailed, which is involved in both bone and shell formation (Fig. 7; Supplementary Table 31, Shell and bone formation).

In addition, many calcium binding proteins (e.g., calcineurin, calponin, and calmodulin) and extracellular matrix proteins (e.g., cadherin, collagen, and fibronectin) have been reported to participate in bone and shell formation (Fig. 7, Supplementary Table 31). This implies that metazoan biomineralization likely originated from a calcium-regulated extracellular matrix system. Furthermore, we also discovered that Hox4, tyrosinase, chitin synthase, perlucin, chitinase, peroxidasin, mucin, and VWA protein are common shell formation-associated components shared by *Lingula* and molluscs (Fig. 7, proteins in orange; Supplementary Table 31, Shell formation), suggesting that this fundamental gene set has been used by their last common ancestor, estimated to be approximately 600 MYA[25] (Fig. 2b).

Additionally, *Lingula* shared with vertebrate genes associated with bone formation including carbohydrate sulfotransferase and fibrillin (Fig. 7, proteins in blue; Supplementary Table 31, Bone formation). There are several enzymes such as glutathione peroxidase, hephaestin, hemicentin, and SVEP1, which cannot be found in shell or bone formation. On the other hand, interestingly, hephaestin and hemicentin are found in the coral skeletal organic matrix[202,203,246]. It implies that these extracellular ion-binding proteins in the biomineral matrix may either be the common features of metazoans that have been lost in vertebrate bones and mollusc shells, or that they arose independently in *Lingula* and corals.

Notably, *Lingula*-specific proteins such as EGF domain-containing fibrillar collagens and alanine-rich proteins may represent the original genes for calcium phosphate-based biomineralization[57]. The duplication of carbohydrate sulfotransferase, chitin synthase, fibronectin, and mucin genes may also contribute to unique features of *Lingula* shells (Fig. 7, proteins in dashed outlines). Taken together, our genomic, transcriptomic, and proteomic analyses of *Lingula* biomineralization show similar patterns to those in molluscs[188] and corals[202], where co-option, domain shuffling, and novel genes are the fundamental mechanisms for metazoan biomineralization.

In conclusion, we proposed possible mechanisms for *Lingula* shell formation (Fig. 7). First, the interaction of myosin head-containing chitin synthases and actin filaments may translate the cytoskeleton organization into an extracellular chitin scaffold. Chitinase in the shell matrix possibly then remodel the chitin scaffold to facilitate the interaction of chitin and chitin-binding proteins. Calcium-binding proteins likely regulate the calcium concentration in the shell matrix and initiate calcium phosphate deposition together with other structural proteins, such as EGF domain-containing fibrillar collagens and alanine-rich proteins.

(b) Evolutionary scenarios of biomineralization

Although fossils of conodont elements might be the first mineralized skeletons of vertebrates dating back to the late Cambrian (~515 MYA)[247], their affinity to the vertebrate teeth is uncertain[248]. Thus, the first vertebrate mineralized bones (i.e., endoskeletons) appeared in the late Ordovician (~450 MYA)[208] much later than lingulid shells (~520 MYA, early Cambrian)[65]. Together with the distant phylogenetic relationship of vertebrates and *Lingula*, it is perhaps not surprising that bones and shells shared different genetic origins. In fact, recent discoveries from Cambrian fossils have changed our ideas about evolution of early molluscs and animal biomineralization. For example, a non-mineralized cephalopod fossil, *Nectocaris*, found in Burgess Shale (~508 MYA, middle Cambrian) suggests that a mineralized shell is a derived character of cephalopods[249]. On the other hand, phylogenomic studies of mollusc phylogeny show that shells may have multiple origins[5,6], which is in agreement with the proteomic studies of mollusc shells[187-189]. Extant molluscs can be divided into two major groups, Conchifera (shell-bearing; Gastropoda, Bivalvia, Scaphopoda, Cephalopoda, and Monoplacophora) and Aculifera (worm-like; Neomeniomorpha, Chaetodermomorpha, and Polyplacophora)[5]. Although conchiferans make shells and aculiferans have only sclerites, both of them use calcium carbonate. While brachiopods have adopted different modes of biomineralization, only the Linguliformea makes shells with calcium phosphate[52] (Supplementary Fig. 28a).

In the light of the close phylogenetic relationship between *Lingula* and molluscs, we hypothesized evolutionary scenarios for the primitive mode of biomineralization in their common ancestors. By comparing chemical and molecular features, three possible primitive modes are presented (Supplementary Fig. 28b-d). First, we propose that calcium phosphate might be the primitive mode of biomineralization, since lingulid brachiopod fossils are abundant in the early Cambrian (Supplementary Fig. 28b). However, this implies a huge number of secondary losses in other lineages, which makes this hypothesis less attractive. On the other hand, calcium carbonate might be primitive, because it is the mode that has been used by most extant brachiopods and molluscs. Relatively few losses are required to fulfill this scenario (Supplementary Fig. 28c). Nevertheless, calcium phosphate and carbonate biominerals appeared almost at the same time during the Cambrian explosion[171]. Although the mollusc-like fossil, *Kimberella*, was found before the Cambrian[250], there is no clear evidence which mode of the biominerals appeared first.

Perhaps the ancestor of lophotrochozoans was non-minerlaized. Supporting evidence comes from another mollusc-like fossil, *Odontogriphus*, in the middle Cambrian. Considered as a stem-group lophotrochozoan, it was shell-less and possessed putative radulae[251]. Thus, we argue that calcification might be a derived feature in molluscs and brachiopods, in which chitin in the

shell may be a synapomorphic character shared by their ancestors. Chitinous scaffold may provide the organic framework for interactions between extracellular matrix and mineral ions (Supplementary Fig. 28d). This idea is supported by data from the embryonic shell of molluscs, where a chitin scaffold is crucial for shell formation[165]. More interestingly, chitin and chitin synthase genes were recently found in vertebrates, expressed in epithelial cells of fishes and amphibians[252]. These suggest an ancient evolutionary origin of epidermal chitin in bilaterian ancestors. The ancestral composition of animal biominerals remains to be resolved. Further comparative genomic and functional studies of lophotrochozoans, such as brachiopods, phoronids, and molluscs will be needed to resolve this question.

Supplementary References

1. Bourlat, S. J., Nielsen, C., Economou, A. D. & Telford, M. J. Testing the new animal phylogeny: a phylum level molecular analysis of the animal kingdom. *Mol. Phylogenet. Evol.* **49**, 23-31 (2008).
2. Hejnol, A. *et al.* Assessing the root of bilaterian animals with scalable phylogenomic methods. *Proc. R. Soc. B* (2009).
3. Struck, T. H. *et al.* Platyzoan paraphyly based on phylogenomic data supports a noncoelomate ancestry of spiralia. *Mol. Biol. Evol.* **31**, 1833-1849 (2014).
4. Tagawa, K. *et al.* A cDNA resource for gene expression studies of a hemichordate, *Ptychodera flava*. *Zoolog. Sci.* **31**, 414-420 (2014).
5. Kocot, K. M. *et al.* Phylogenomics reveals deep molluscan relationships. *Nature* **477**, 452-456 (2011).
6. Smith, S. A. *et al.* Resolving the evolutionary relationships of molluscs with phylogenomic tools. *Nature* **480**, 364-367 (2011).
7. Sperling, E. A., Pisani, D. & Peterson, K. J. Molecular paleobiological insights into the origin of the Brachiopoda. *Evol. Dev.* **13**, 290-303 (2011).
8. Simakov, O. *et al.* Insights into bilaterian evolution from three spiralian genomes. *Nature* **493**, 526-531 (2013).
9. Zhang, G. *et al.* The oyster genome reveals stress adaptation and complexity of shell formation. *Nature* **490**, 49-54 (2012).
10. Takeuchi, T. *et al.* Draft genome of the pearl oyster *Pinctada fucata*: a platform for understanding bivalve biology. *DNA Res.* **19**, 117-130 (2012).
11. Dehal, P. *et al.* The draft genome of *Ciona intestinalis*: Insights into chordate and vertebrate origins. *Science* **298**, 2157-2167 (2002).
12. Putnam, N. H. *et al.* The amphioxus genome and the evolution of the chordate karyotype. *Nature* **453**, 1064-1071 (2008).
13. Sodergren, E. *et al.* The genome of the sea urchin *Strongylocentrotus purpuratus*. *Science* **314**, 941-952 (2006).
14. Shinzato, C. *et al.* Using the *Acropora digitifera* genome to understand coral responses to environmental change. *Nature* **476**, 320-323 (2011).
15. Putnam, N. H. *et al.* Sea anemone genome reveals ancestral eumetazoan gene repertoire and genomic organization. *Science* **317**, 86-94 (2007).
16. Srivastava, M. *et al.* The *Trichoplax* genome and the nature of placozoans. *Nature* **454**, 955-960 (2008).
17. Srivastava, M. *et al.* The *Amphimedon queenslandica* genome and the evolution of animal complexity. *Nature* **466**, 720-726 (2010).
18. Ryan, J. F. *et al.* The genome of the ctenophore *Mnemiopsis leidyi* and its implications for cell type evolution. *Science* **342**, 1242592 (2013).
19. Moroz, L. L. *et al.* The ctenophore genome and the evolutionary origins of neural systems. *Nature* **510**, 109-114 (2014).
20. Dunn, C. W. *et al.* Broad phylogenomic sampling improves resolution of the animal tree of life. *Nature* **452**, 745-749 (2008).
21. Helmkampf, M., Bruchhaus, I. & Hausdorf, B. Phylogenomic analyses of lophophorates (brachiopods, phoronids and bryozoans) confirm the Lophotrochozoa concept. *Proc. Biol. Sci.* **275**, 1927-1933 (2008).
22. Paps, J., Baguna, J. & Riutort, M. Bilaterian phylogeny: a broad sampling of 13 nuclear genes provides a new Lophotrochozoa phylogeny and supports a paraphyletic basal acoelomorpha. *Mol. Biol. Evol.* **26**, 2397-2406 (2009).
23. Paps, J., Baguna, J. & Riutort, M. Lophotrochozoa internal phylogeny: new insights from an up-to-date analysis of nuclear ribosomal genes. *Proc. Biol. Sci.* **276**, 1245-1254 (2009).
24. Hausdorf, B., Helmkampf, M., Nesnidal, M. P. & Bruchhaus, I. Phylogenetic relationships within the lophophorate lineages (Ectoprocta, Brachiopoda and Phoronida). *Mol. Phylogenet. Evol.* **55**, 1121-1127 (2010).

25. Erwin, D. H. *et al.* The Cambrian conundrum: early divergence and later ecological success in the early history of animals. *Science* **334**, 1091-1097 (2011).
26. Andrade, S. C. *et al.* A transcriptomic approach to ribbon worm systematics (nemertea): resolving the pilidiophora problem. *Mol. Biol. Evol.* **31**, 3206-3215 (2014).
27. Nederbragt, A. J., van Loon, A. E. & Dictus, W. J. Expression of *Patella vulgata* orthologs of *engrailed* and *dpp-BMP2/4* in adjacent domains during molluscan shell development suggests a conserved compartment boundary mechanism. *Dev. Biol.* **246**, 341-355 (2002).
28. Chen, G., Deng, C. & Li, Y. P. TGF-beta and BMP signaling in osteoblast differentiation and bone formation. *Int. J. Biol. Sci.* **8**, 272-288 (2012).
29. Yan, F. *et al.* Molecular characterization of the BMP7 gene and its potential role in shell formation in *Pinctada martensii*. *Int. J. Mol. Sci.* **15**, 21215-21228 (2014).
30. Liu, G., Huan, P. & Liu, B. Cloning and expression patterns of two Smad genes during embryonic development and shell formation of the Pacific oyster *Crassostrea gigas*. *Chin. J. Oceanol. Limnol.* **32**, 1224-1231 (2014).
31. Iijima, M., Takeuchi, T., Sarashina, I. & Endo, K. Expression patterns of *engrailed* and *dpp* in the gastropod *Lymnaea stagnalis*. *Dev. Genes Evol.* **218**, 237-251 (2008).
32. Deckelbaum, R. A., Majithia, A., Booker, T., Henderson, J. E. & Loomis, C. A. The homeoprotein *engrailed 1* has pleiotropic functions in calvarial intramembranous bone formation and remodeling. *Development* **133**, 63-74 (2006).
33. Li, C. *et al.* Calcineurin plays an important role in the shell formation of pearl oyster (*Pinctada fucata*). *Mar. Biotechnol.* **12**, 100-110 (2010).
34. Sun, L. *et al.* Calcineurin regulates bone formation by the osteoblast. *Proc. Natl. Acad. Sci. USA* **102**, 17130-17135 (2005).
35. Shi, Y. *et al.* Characterization of the pearl oyster (*Pinctada martensii*) mantle transcriptome unravels biomineralization genes. *Mar. Biotechnol.* **15**, 175-187 (2013).
36. Su, N. *et al.* Overexpression of H1 calponin in osteoblast lineage cells leads to a decrease in bone mass by disrupting osteoblast function and promoting osteoclast formation. *J. Bone Miner. Res.* **28**, 660-671 (2013).
37. Yan, Z. *et al.* Biomineralization: functions of calmodulin-like protein in the shell formation of pearl oyster. *Biochim. Biophys. Acta* **1770**, 1338-1344 (2007).
38. Zayzafoon, M., Fulzele, K. & McDonald, J. M. Calmodulin and calmodulin-dependent kinase IIalpha regulate osteoblast differentiation by controlling c-fos expression. *J. Biol. Chem.* **280**, 7049-7059 (2005).
39. Marie, P. J. Role of N-cadherin in bone formation. *J. Cell Physiol.* **190**, 297-305 (2002).
40. Miyamoto, H. *et al.* A carbonic anhydrase from the nacreous layer in oyster pearls. *Proc. Natl. Acad. Sci. USA* **93**, 9657-9660 (1996).
41. Lehenkari, P., Hentunen, T. A., Laitala-Leinonen, T., Tuukkanen, J. & Vaananen, H. K. Carbonic anhydrase II plays a major role in osteoclast differentiation and bone resorption by effecting the steady state intracellular pH and Ca2+. *Exp. Cell Res.* **242**, 128-137 (1998).
42. Nudelman, F. *et al.* The role of collagen in bone apatite formation in the presence of hydroxyapatite nucleation inhibitors. *Nat. Mater.* **9**, 1004-1009 (2010).
43. Bentmann, A. *et al.* Circulating fibronectin affects bone matrix, whereas osteoblast fibronectin modulates osteoblast function. *J. Bone Miner. Res.* **25**, 706-715 (2010).
44. Samadi, L. & Steiner, G. Involvement of Hox genes in shell morphogenesis in the encapsulated development of a top shell gastropod (*Gibbula varia L.*). *Dev. Genes Evol.* **219**, 523-530 (2009).
45. Zhang, C., Xie, L., Huang, J., Chen, L. & Zhang, R. A novel putative tyrosinase involved in periostracum formation from the pearl oyster (*Pinctada fucata*). *Biochem. Biophys. Res. Commun.* **342**, 632-639 (2006).
46. Weiss, I. M., Schonitzer, V., Eichner, N. & Sumper, M. The chitin synthase involved in marine bivalve mollusk shell formation contains a myosin domain. *FEBS Lett.* **580**, 1846-1852 (2006).
47. Marie, B. *et al.* The shell-forming proteome of *Lottia gigantea* reveals both deep conservations and lineage-specific novelties. *FEBS J.* **280**, 214-232 (2013).
48. Mann, K., Weiss, I. M., Andre, S., Gabius, H. J. & Fritz, M. The amino-acid sequence of the abalone (*Haliotis laevigata*) nacre protein perlucin. Detection of a functional C-type lectin domain with galactose/mannose specificity. *Eur. J. Biochem.* **267**, 5257-5264 (2000).

49. Marin, F., Corstjens, P., de Gaulejac, B., de Vrind-De Jong, E. & Westbroek, P. Mucins and molluscan calcification. Molecular characterization of mucoperlin, a novel mucin-like protein from the nacreous shell layer of the fan mussel *Pinna nobilis* (Bivalvia, pteriomorphia). *J. Biol. Chem.* **275**, 20667-20675 (2000).
50. Hermanns, P. *et al.* Congenital joint dislocations caused by carbohydrate sulfotransferase 3 deficiency in recessive Larsen syndrome and humero-spinal dysostosis. *Am. J. Hum. Genet.* **82**, 1368-1374 (2008).
51. Nistala, H., Lee-Arteaga, S., Smaldone, S., Siciliano, G. & Ramirez, F. Extracellular microfibrils control osteoblast-supported osteoclastogenesis by restricting TGF{beta} stimulation of RANKL production. *J. Biol. Chem.* **285**, 34126-34133 (2010).
52. Williams, A., Cusack, M. & Mackay, S. Collagenous chitinophosphatic shell of the brachiopod *Lingula*. *Phil. Trans. R. Soc. B* **346**, 223-266 (1994).
53. Bitner, M. A. & Cohen, B. L. Brachiopoda. in *eLS* (John Wiley & Sons, Ltd, 2013).
54. Yatsu, N. On the development of *Lingula anatina*. *J. Coll. Sci. Imp. Univ. Tokyo* **17**, 1-112 (1902).
55. Emig, C. C. Ecology of the inarticulated brachiopods. in *Treatise on Invertebrate Paleontology. Part H. Brachiopoda.* Vol. 1 (ed R. L. Kaesler) 473-495 (Geological Society of America and University of Kansas, 1997).
56. Savazzi, E. Burrowing in the inarticulate brachiopod *Lingula anatina*. *Palaeogeogr. Palaeocl.* **85**, 101-106 (1991).
57. Williams, A., Carlson, S. J., Brunton, C. H. C., Holmer, L. E. & Popov, L. A supra-ordinal classification of the Brachiopoda. *Phil. Trans. R. Soc. B* **351**, 1171-1193 (1996).
58. Cook, P. J. & Shergold, J. H. Phosphorus, phosphorites and skeletal evolution at the Precambrian-Cambrian boundary. *Nature* **308**, 231-236 (1984).
59. Gould, S. J. & Calloway, C. B. Clams and brachiopods; ships that pass in the night. *Paleobiology* **6**, 383-396 (1980).
60. Darwin, C. *On the Origin of Species by Means of Natural Selection*. (Murray London, 1859).
61. Emig, C. C. Proof that *Lingula* (Brachiopoda) is not a living fossil, and amended diagnoses of the Family Lingulidae. *Carnets de Géologie / Notebooks on Geology letter*, 1-8 (2003).
62. Emig, C. C. On the history of the names *Lingula, anatina,* and on the confusion of the forms assigned them among the Brachiopoda. *Carnets de Géologie / Notebooks on Geology letter,* 1-13 (2008).
63. Williams, A. & Cusack, M. Evolution of a rhythmic lamination in the organophosphatic shells of brachiopods. *J. Struct. Biol.* **126**, 227-240 (1999).
64. Cusack, M., Williams, A. & Buckman, J. O. Chemico-structural evolution of linguloid brachiopod shells. *Palaeontology* **42**, 799-840 (1999).
65. Zhang, Z., Shu, D., Han, J. & Liu, J. Morpho-anatomical differences of the Early Cambrian Chengjiang and Recent lingulids and their implications. *Acta Zool.* **86**, 277-288 (2005).
66. Yang, S., Lai, X., Sheng, G. & Wang, S. Deep genetic divergence within a "living fossil" brachiopod *Lingula anatina*. *J. Paleontol.* **87**, 902-908 (2013).
67. Nishizawa, A., Sarashina, I., Tsujimoto, Y., IIjima, M., Endo, K. . Artificial fertilization, early development and chromosome numbers in the brachiopod *Lingula anatina*. *Palaeontology* **84**, 1-8 (2010).
68. Tagawa, K., Nishino, A., Humphreys, T. & Satoh, N. The spawning and early development of the Hawaiian acorn worm (hemichordate), *Ptychodera flava*. *Zool. Sci.* **15**, 85-91 (1998).
69. Kume, M. The spawning of *Lingula*. *Nat. Sci. Rep. Ochanomizu U.* **6**, 215-223 (1956).
70. Wheeler, D. A. *et al.* The complete genome of an individual by massively parallel DNA sequencing. *Nature* **452**, 872-876 (2008).
71. Bentley, D. R. *et al.* Accurate whole human genome sequencing using reversible terminator chemistry. *Nature* **456**, 53-59 (2008).
72. Eid, J. *et al.* Real-time DNA sequencing from single polymerase molecules. *Science* **323**, 133-138 (2009).
73. Andrews, S. *FastQC v0.11.2*, <http://www.bioinformatics.babraham.ac.uk/projects/fastqc/> (2010-2014).

74. Schmieder, R. & Edwards, R. Quality control and preprocessing of metagenomic datasets. *Bioinformatics* **27**, 863-864 (2011).
75. Bolger, A. M., Lohse, M. & Usadel, B. Trimmomatic: a flexible trimmer for Illumina sequence data. *Bioinformatics* **30**, 2114-2120 (2014).
76. Van Nieuwerburgh, F. *et al.* Illumina mate-paired DNA sequencing-library preparation using Cre-Lox recombination. *Nucleic Acids Res.* **40**, e24 (2012).
77. Leggett, R. M., Clavijo, B. J., Clissold, L., Clark, M. D. & Caccamo, M. NextClip: an analysis and read preparation tool for Nextera Long Mate Pair libraries. *Bioinformatics* **30**, 566-568 (2014).
78. Caruccio, N. Preparation of next-generation sequencing libraries using Nextera technology: simultaneous DNA fragmentation and adaptor tagging by in vitro transposition. *Methods. Mol. Biol.* **733**, 241-255 (2011).
79. Boetzer, M., Henkel, C. V., Jansen, H. J., Butler, D. & Pirovano, W. Scaffolding pre-assembled contigs using SSPACE. *Bioinformatics* **27**, 578-579 (2011).
80. Chaisson, M. J. & Tesler, G. Mapping single molecule sequencing reads using basic local alignment with successive refinement (BLASR): application and theory. *BMC Bioinformatics* **13**, 238 (2012).
81. Boetzer, M. & Pirovano, W. SSPACE-LongRead: scaffolding bacterial draft genomes using long read sequence information. *BMC Bioinformatics* **15**, 211 (2014).
82. Luo, R. *et al.* SOAPdenovo2: an empirically improved memory-efficient short-read *de novo* assembler. *Gigascience* **1**, 18 (2012).
83. Marçais, G. & Kingsford, C. A fast, lock-free approach for efficient parallel counting of occurrences of k-mers. *Bioinformatics* **27**, 764-770 (2011).
84. Price, A. L., Jones, N. C. & Pevzner, P. A. *De novo* identification of repeat families in large genomes. *Bioinformatics* **21**, i351-i358 (2005).
85. Smit, A. F. A. & Hubley, R. *RepeatModeler Open-1.0.*, <http://www.repeatmasker.org> (2008-2010).
86. Parra, G., Bradnam, K. & Korf, I. CEGMA: a pipeline to accurately annotate core genes in eukaryotic genomes. *Bioinformatics* **23**, 1061-1067 (2007).
87. Kent, W. J. BLAT--the BLAST-like alignment tool. *Genome Res.* **12**, 656-664 (2002).
88. Ryan, J. F. Baa.pl: A tool to evaluate *de novo* genome assemblies with RNA transcripts. *arXiv:1309.2087* (2013).
89. Stanke, M., Diekhans, M., Baertsch, R. & Haussler, D. Using native and syntenically mapped cDNA alignments to improve *de novo* gene finding. *Bioinformatics* **24**, 637-644 (2008).
90. Haas, B. J. *et al.* Improving the *Arabidopsis* genome annotation using maximal transcript alignment assemblies. *Nucleic Acids Res.* **31**, 5654-5666 (2003).
91. Camacho, C. *et al.* BLAST+: architecture and applications. *BMC Bioinformatics* **10**, 421 (2009).
92. Conesa, A. & Götz, S. Blast2GO: A Comprehensive Suite for Functional Analysis in Plant Genomics. *Int. J. Plant Genomics* **2008**, 619832 (2008).
93. Grabherr, M. G. *et al.* Full-length transcriptome assembly from RNA-Seq data without a reference genome. *Nat. Biotechnol.* **29**, 644-652 (2011).
94. Haas, B. J. *et al. De novo* transcript sequence reconstruction from RNA-seq using the Trinity platform for reference generation and analysis. *Nat. Protoc.* **8**, 1494-1512 (2013).
95. Langmead, B. & Salzberg, S. L. Fast gapped-read alignment with Bowtie 2. *Nat. Meth.* **9**, 357-359 (2012).
96. Li, B. & Dewey, C. N. RSEM: accurate transcript quantification from RNA-Seq data with or without a reference genome. *BMC Bioinformatics* **12**, 323 (2011).
97. Li, W. & Godzik, A. Cd-hit: a fast program for clustering and comparing large sets of protein or nucleotide sequences. *Bioinformatics* **22**, 1658-1659 (2006).
98. Rice, P., Longden, I. & Bleasby, A. EMBOSS: the European Molecular Biology Open Software Suite. *Trends Genet.* **16**, 276-277 (2000).
99. Finn, R. D. *et al.* The Pfam protein families database. *Nucleic Acids Res.* **36**, D281-D288 (2008).
100. Eddy, S. R. Accelerated Profile HMM Searches. *PLoS Comput. Biol.* **7**, e1002195 (2011).

101. UniProt-Consortium. The Universal Protein Resource (UniProt). *Nucleic Acids Res.* **35**, D193-197 (2007).
102. Yamada, L., Saito, T., Taniguchi, H., Sawada, H. & Harada, Y. Comprehensive egg coat proteome of the ascidian *Ciona intestinalis* reveals gamete recognition molecules involved in self-sterility. *Journal of Biological Chemistry* **284**, 9402-9410 (2009).
103. Araki, Y. *et al.* A surface glycoprotein indispensable for gamete fusion in the social amoeba *Dictyostelium discoideum*. *Eukaryotic Cell* **11**, 638-644 (2012).
104. Gupta, N. & Pevzner, P. A. False discovery rates of protein identifications: a strike against the two-peptide rule. *J. Proteome. Res.* **8**, 4173-4181 (2009).
105. Gasteiger, E. *et al.* Protein identification and analysis tools on the ExPASy server. in *The Proteomics Protocols Handbook* (ed JohnM Walker) Ch. 52, 571-607 (Humana Press, 2005).
106. Petersen, T. N., Brunak, S., von Heijne, G. & Nielsen, H. SignalP 4.0: discriminating signal peptides from transmembrane regions. *Nat. Methods* **8**, 785-786 (2011).
107. McGuffin, L. J., Bryson, K. & Jones, D. T. The PSIPRED protein structure prediction server. *Bioinformatics* **16**, 404-405 (2000).
108. Heger, A. & Holm, L. Rapid automatic detection and alignment of repeats in protein sequences. *Proteins* **41**, 224-237 (2000).
109. Roy, A., Kucukural, A. & Zhang, Y. I-TASSER: a unified platform for automated protein structure and function prediction. *Nat. Protocols* **5**, 725-738 (2010).
110. Field, K. G. *et al.* Molecular phylogeny of the animal kingdom. *Science* **239**, 748-753 (1988).
111. de Rosa, R. *et al.* Hox genes in brachiopods and priapulids and protostome evolution. *Nature* **399**, 772-776 (1999).
112. Cohen, B. L., Holmer, L. E. & Lüter, C. The brachiopod fold: a neglected body plan hypothesis. *Palaeontology* **46**, 59-65 (2003).
113. Vinther, J. & Nielsen, C. The Early Cambrian Halkieria is a mollusc. *Zool. Scripta* **34**, 81-89 (2005).
114. Zhang, Z. F. *et al.* An early Cambrian agglutinated tubular lophophorate with brachiopod characters. *Sci. Rep.* **4** (2014).
115. Altenburger, A., Wanninger, A. & Holmer, L. Metamorphosis in Craniiformea revisited: *Novocrania anomala* shows delayed development of the ventral valve. *Zoomorphology* **132**, 379-387 (2013).
116. Cohen, B. L. & Weydmann, A. Molecular evidence that phoronids are a subtaxon of brachiopods (Brachiopoda: Phoronata) and that genetic divergence of metazoan phyla began long before the early Cambrian. *Org. Divers. Evol.* **5**, 253-273 (2005).
117. Cohen, B. L. Rerooting the rDNA gene tree reveals phoronids to be 'brachiopods without shells'; dangers of wide taxon samples in metazoan phylogenetics (Phoronida; Brachiopoda). *Zool. J. Linn. Soc.* **167**, 82-92 (2013).
118. Nesnidal, M. *et al.* New phylogenomic data support the monophyly of Lophophorata and an Ectoproct-Phoronid clade and indicate that Polyzoa and Kryptrochozoa are caused by systematic bias. *BMC Evol. Biol.* **13**, 253 (2013).
119. Giribet, G., Dunn, C. W., Edgecombe, G. D., Hejnol, A., Martindale, M. Q., Rouse, G. W. Assembling the spiralian tree of life. in *Animal Evolution: Genomes, Fossils, and Trees* (ed M.J. Telford) Ch. 6, 52-64 (2009).
120. Cavalier-Smith, T. A revised six-kingdom system of life. *Biol. Rev. Camb. Philos. Soc.* **73**, 203-266 (1998).
121. Fischer, S. *et al.* Using OrthoMCL to assign proteins to OrthoMCL-DB groups or to cluster proteomes into new ortholog groups. *Curr. Protoc. Bioinformatics* **Chapter 6**, Unit 6 12 11-19 (2011).
122. Li, L., Stoeckert, C. J., Jr. & Roos, D. S. OrthoMCL: identification of ortholog groups for eukaryotic genomes. *Genome Res.* **13**, 2178-2189 (2003).
123. Wolf, Y. I. & Koonin, E. V. A tight link between orthologs and bidirectional best hits in bacterial and archaeal genomes. *Genome Biol. Evol.* **4**, 1286-1294 (2012).
124. Katoh, K., Misawa, K., Kuma, K. i. & Miyata, T. MAFFT: a novel method for rapid multiple sequence alignment based on fast Fourier transform. *Nucleic Acids Res.* **30**, 3059-3066 (2002).

125. Castresana, J. Selection of conserved blocks from multiple alignments for their use in phylogenetic analysis. *Mol Biol Evol* **17**, 540-552 (2000).
126. Capella-Gutiérrez, S., Silla-Martínez, J. M. & Gabaldón, T. trimAl: a tool for automated alignment trimming in large-scale phylogenetic analyses. *Bioinformatics* **25**, 1972-1973 (2009).
127. Le, S. Q. & Gascuel, O. An improved general amino acid replacement matrix. *Mol. Biol. Evol.* **25**, 1307-1320 (2008).
128. Tavaré, S. Some probabilistic and statistical problems in the analysis of DNA sequences. *Lect. Math. Life Sci.* **17**, 57-86 (1986).
129. Stamatakis, A. RAxML version 8: A tool for phylogenetic analysis and post-analysis of large phylogenies. *Bioinformatics* (2014).
130. Lartillot, N., Lepage, T. & Blanquart, S. PhyloBayes 3: a Bayesian software package for phylogenetic reconstruction and molecular dating. *Bioinformatics* **25**, 2286-2288 (2009).
131. Amemiya, C. T. *et al.* The African coelacanth genome provides insights into tetrapod evolution. *Nature* **496**, 311-316 (2013).
132. Bairoch, A. & Apweiler, R. The SWISS-PROT protein sequence database and its supplement TrEMBL in 2000. *Nucleic Acids Res.* **28**, 45-48 (2000).
133. Krylov, D. M., Wolf, Y. I., Rogozin, I. B. & Koonin, E. V. Gene loss, protein sequence divergence, gene dispensability, expression level, and interactivity are correlated in eukaryotic evolution. *Genome Res.* **13**, 2229-2235 (2003).
134. Hahn, M. W., Han, M. V. & Han, S. G. Gene family evolution across 12 *Drosophila* genomes. *PLoS Genet.* **3**, e197 (2007).
135. Rentzsch, F. *et al.* Asymmetric expression of the BMP antagonists chordin and gremlin in the sea anemone Nematostella vectensis: implications for the evolution of axial patterning. *Dev. Biol.* **296**, 375-387 (2006).
136. Kuo, D. H. & Weisblat, D. A. A new molecular logic for BMP-mediated dorsoventral patterning in the leech *Helobdella*. *Curr. Biol.* **21**, 1282-1288 (2011).
137. Pearson, J. C., Lemons, D. & McGinnis, W. Modulating Hox gene functions during animal body patterning. *Nat. Rev. Genet.* **6**, 893-904 (2005).
138. Demuth, J. P., De Bie, T., Stajich, J. E., Cristianini, N. & Hahn, M. W. The evolution of mammalian gene families. *PloS One* **1**, e85 (2006).
139. De Bie, T., Cristianini, N., Demuth, J. P. & Hahn, M. W. CAFE: a computational tool for the study of gene family evolution. *Bioinformatics* **22**, 1269-1271 (2006).
140. Zhang, Z. *et al.* ParaAT: a parallel tool for constructing multiple protein-coding DNA alignments. *Biochem. Biophys. Res. Commun.* **419**, 779-781 (2012).
141. Suyama, M., Torrents, D. & Bork, P. PAL2NAL: robust conversion of protein sequence alignments into the corresponding codon alignments. *Nucleic Acids Res.* **34**, W609-612 (2006).
142. Wang, D., Zhang, Y., Zhang, Z., Zhu, J. & Yu, J. KaKs_Calculator 2.0: a toolkit incorporating gamma-series methods and sliding window strategies. *Genomics Proteomics Bioinformatics* **8**, 77-80 (2010).
143. Kanehisa, M. *et al.* Data, information, knowledge and principle: back to metabolism in KEGG. *Nucleic Acids Res.* **42**, D199-205 (2014).
144. Guindon, S. *et al.* New algorithms and methods to estimate maximum-likelihood phylogenies: assessing the performance of PhyML 3.0. *Syst. Biol.* **59**, 307-321 (2010).
145. Saitou, N. & Nei, M. The neighbor-joining method: a new method for reconstructing phylogenetic trees. *Mol. Biol. Evol.* **4**, 406-425 (1987).
146. Jones, D. T., Taylor, W. R. & Thornton, J. M. The rapid generation of mutation data matrices from protein sequences. *Comput. Appl. Biosci.* **8**, 275-282 (1992).
147. Tamura, K., Stecher, G., Peterson, D., Filipski, A. & Kumar, S. MEGA6: Molecular Evolutionary Genetics Analysis version 6.0. *Mol. Biol. Evol.* **30**, 2725-2729 (2013).
148. Bardou, P., Mariette, J., Escudie, F., Djemiel, C. & Klopp, C. jvenn: an interactive Venn diagram viewer. *BMC Bioinformatics* **15**, 293 (2014).
149. Huang da, W., Sherman, B. T. & Lempicki, R. A. Systematic and integrative analysis of large gene lists using DAVID bioinformatics resources. *Nat. Protoc.* **4**, 44-57 (2009).

150. Mi, H., Muruganujan, A. & Thomas, P. D. PANTHER in 2013: modeling the evolution of gene function, and other gene attributes, in the context of phylogenetic trees. *Nucleic Acids Res.* **41**, D377-386 (2013).
151. Cao, Z. *et al.* The genome of *Mesobuthus martensii* reveals a unique adaptation model of arthropods. *Nat Commun* **4**, 2602 (2013).
152. Thiele, H. *et al.* Loss of chondroitin 6-O-sulfotransferase-1 function results in severe human chondrodysplasia with progressive spinal involvement. *Proc. Natl. Acad. Sci. USA* **101**, 10155-10160 (2004).
153. Rinaudo, M. Chitin and chitosan: Properties and applications. *Prog. Polym. Sci.* **31**, 603-632 (2006).
154. Kurita, K. Chitin and chitosan: functional biopolymers from marine crustaceans. *Mar. Biotechnol.* **8**, 203-226 (2006).
155. Merzendorfer, H. & Zimoch, L. Chitin metabolism in insects: structure, function and regulation of chitin synthases and chitinases. *J. Exp. Biol.* **206**, 4393-4412 (2003).
156. Weiner, S., Traub, W. & Parker, S. B. Macromolecules in mollusc shells and their functions in biomineralization. *Phil. Trans. R. Soc. Lond. B* **304**, 425-434 (1984).
157. Weaver, J. C. *et al.* Analysis of an ultra hard magnetic biomineral in chiton radular teeth. *Mater. Today* **13**, 42-52 (2010).
158. Miserez, A., Schneberk, T., Sun, C., Zok, F. W. & Waite, J. H. The transition from stiff to compliant materials in squid beaks. *Science* **319**, 1816-1819 (2008).
159. Hausen, H. Chaetae and chaetogenesis in polychaetes (Annelida). *Hydrobiologia* **535-536**, 37-52 (2005).
160. Leise, E. & Cloney, R. Chiton integument: Ultrastructure of the sensory hairs of *Mopalia muscosa* (Mollusca: Polyplacophora). *Cell Tissue Res.* **223**, 43-59 (1982).
161. Tanaka, K., Katsura, N., Saku, T. & Kasuga, S. Composite texture of chitin and keratin in an animal organ, *Lingula* seta. *Polym. J.* **20**, 119-123 (1988).
162. Hegedus, D., Erlandson, M., Gillott, C. & Toprak, U. New insights into peritrophic matrix synthesis, architecture, and function. *Annu. Rev. Entomol.* **54**, 285-302 (2009).
163. Wagner, G. P., Lo, J., Laine, R. & Almeder, M. Chitin in the epidermal cuticle of a vertebrate (*Paralipophrys trigloides*, Blenniidae, Teleostei). *Experientia* **49**, 317-319 (1993).
164. Zakrzewski, A. C. *et al.* Early divergence, broad distribution, and high diversity of animal chitin synthases. *Genome Biol. Evol.* **6**, 316-325 (2014).
165. Schonitzer, V. & Weiss, I. The structure of mollusc larval shells formed in the presence of the chitin synthase inhibitor Nikkomycin Z. *BMC Struct. Biol.* **7**, 71 (2007).
166. Guerriero, G. Putative chitin synthases from *Branchiostoma floridae* show extracellular matrix-related domains and mosaic structures. *Genomics Proteomics Bioinformatics* **10**, 197-207 (2012).
167. Richardson, J. R. Pedicle structure of articulate brachiopods. *J. R. Soc. NZ* **9**, 415-436 (1979).
168. Knoll, A. H. Biomineralization and evolutionary history. *Rev. Mineral. Geochem.* **54**, 329-356 (2003).
169. Cusack, M. & Freer, A. Biomineralization: elemental and organic influence in carbonate systems. *Chem. Rev.* **108**, 4433-4454 (2008).
170. Lowenstam, H. Minerals formed by organisms. *Science* **211**, 1126-1131 (1981).
171. Marin, F., Luquet, G., Marie, B. & Medakovic, D. Molluscan shell proteins: primary structure, origin, and evolution. *Curr. Top. Dev. Biol.* **80**, 209-276 (2008).
172. Suzuki, M. & Nagasawa, H. Mollusk shell structures and their formation mechanism. *Can. J. Zool.* **91**, 349-366 (2013).
173. Sun, J. & Bhushan, B. Hierarchical structure and mechanical properties of nacre: a review. *RSC Advances* **2**, 7617-7632 (2012).
174. Iwata, K. Ultrastructure and mineralization of the shell of *Lingula unguis* Linne, (inarticualte brachiopod). *J. Faculty Sci. Hokkaido Univ. 4, Geol. Mineral.* **20**, 35-65 (1981).
175. Clarke, F. W. & Wheeler, W. C. The composition of brachiopod shells. *Proc. Natl. Acad. Sci. USA* **1**, 262-266 (1915).

176. Merkel, C. *et al.* Mechanical properties of modern calcite- (*Mergerlia truncata*) and phosphate-shelled brachiopods (*Discradisca stella* and *Lingula anatina*) determined by nanoindentation. *J. Struct. Biol.* **168**, 396-408 (2009).
177. Jope, M. Brachiopod shell proteins: their functions and taxonomic significance. *Amer. Zool.* **17**, 133-140 (1977).
178. Weiner, S. & Dove, P. An overview of biomineralization processes and the problem of the vital effect. *Rev. Mineral. Geochem.* **54**, 1-29 (2003).
179. Golub, E. E. Role of matrix vesicles in biomineralization. *Biochim. Biophys. Acta.* **1790**, 1592-1598 (2009).
180. Boonrungsiman, S. *et al.* The role of intracellular calcium phosphate in osteoblast-mediated bone apatite formation. *Proc. Natl. Acad. Sci. USA* **109**, 14170-14175 (2012).
181. Beniash, E., Addadi, L. & Weiner, S. Cellular control over spicule formation in sea urchin embryos: A structural approach. *J. Struct. Biol.* **125**, 50-62 (1999).
182. Beniash, E., Aizenberg, J., Addadi, L. & Weiner, S. Amorphous calcium carbonate transforms into calcite during sea urchin larval spicule growth. *Proc. R. Soc. B* **264**, 461-465 (1997).
183. Politi, Y. *et al.* Transformation mechanism of amorphous calcium carbonate into calcite in the sea urchin larval spicule. *Proc. Natl. Acad. Sci. USA* **105**, 17362-17366 (2008).
184. Furuhashi, T., Schwarzinger, C., Miksik, I., Smrz, M. & Beran, A. Molluscan shell evolution with review of shell calcification hypothesis. *Comp. Biochem. Physiol. B Biochem. Mol. Biol.* **154**, 351-371 (2009).
185. Wang, X. *et al.* Oyster shell proteins originate from multiple organs and their probable transport pathway to the shell formation front. *PLoS One* **8**, e66522 (2013).
186. Mount, A. S., Wheeler, A. P., Paradkar, R. P. & Snider, D. Hemocyte-mediated shell mineralization in the eastern oyster. *Science* **304**, 297-300 (2004).
187. Jackson, D. J. *et al.* A rapidly evolving secretome builds and patterns a sea shell. *BMC Biol.* **4**, 40 (2006).
188. Jackson, D. J. *et al.* Parallel evolution of nacre building gene sets in molluscs. *Mol. Biol. Evol.* **27**, 591-608 (2010).
189. Sarashina, I. & Endo, K. Skeletal matrix proteins of invertebrate animals: Comparative analysis of their amino acid sequences. *Paleontol. Res.* **10**, 311-336 (2006).
190. Sarashina, I. *et al.* Molecular evolution and functionally important structures of molluscan Dermatopontin: implications for the origins of molluscan shell matrix proteins. *J. Mol. Evol.* **62**, 307-318 (2006).
191. Ettensohn, C. A. Horizontal transfer of the *msp130* gene supported the evolution of metazoan biomineralization. *Evol. Dev.* **16**, 139-148 (2014).
192. Jackson, D. J., Macis, L., Reitner, J. & Worheide, G. A horizontal gene transfer supported the evolution of an early metazoan biomineralization strategy. *BMC Evol. Biol.* **11**, 238 (2011).
193. Bengtson, S., Farmer, J. D., Fedonkin, M. A., Lipps, J. H. & Runnegar, B. N. The Proterozoic-Early Cambrian evolution of metaphytes and metazoans. in *The Proterozoic Biosphere: A Multidisciplinary Study* (eds Schopf J. W. & Klein C.) (Cambridge, 1992).
194. Kawasaki, K., Buchanan, A. V. & Weiss, K. M. Biomineralization in humans: making the hard choices in life. *Annu. Rev. Genet.* **43**, 119-142 (2009).
195. McConnell, D. Inorganic constituents in the shell of the linving brachiopod *Lingula*. *Geol. Soc. Am. Bull.* **74**, 363-364 (1963).
196. Neary, M. T. *et al.* Contrasts between organic participation in apatite biomineralization in brachiopod shell and vertebrate bone identified by nuclear magnetic resonance spectroscopy. *J. R. Soc. Interface* **8**, 282-288 (2011).
197. Rohanizadeh, R. & Legeros, R. Z. Mineral phase in linguloid brachiopod shell: *Lingula adamsi*. *Lethaia* **40**, 61-68 (2007).
198. Marin, F., Smith, M., Isa, Y., Muyzer, G. & Westbroek, P. Skeletal matrices, muci, and the origin of invertebrate calcification. *Proc. Natl. Acad. Sci. USA* **93**, 1554-1559 (1996).
199. Chuang, S. H. The circulation of coelomic fluid in *Lingula unguis*. *Proc. Zool. Soc. Lond.* **143**, 221-237 (1964).

200. Yoshikawa, H. *et al.* Mice lacking smooth muscle calponin display increased bone formation that is associated with enhancement of bone morphogenetic protein responses. *Genes Cells* **3**, 685-695 (1998).
201. Zhan, X. *et al.* Expressed sequence tags 454 sequencing and biomineralization gene expression for pearl sac of the pearl oyster, *Pinctada fucata martensii*. *Aquac. Res.* (2013).
202. Ramos-Silva, P. *et al.* The skeletal proteome of the coral *Acropora millepora*: the evolution of calcification by co-option and domain shuffling. *Mol. Biol. Evol.* **30**, 2099-2112 (2013).
203. Ramos-Silva, P. *et al.* The skeleton of the staghorn coral *Acropora millepora*: molecular and structural characterization. *PLoS One* **9**, e97454 (2014).
204. Mukaka, M. M. Statistics corner: A guide to appropriate use of correlation coefficient in medical research. *Malawi Med. J.* **24**, 69-71 (2012).
205. Mackay, S. & Hewitt, R. A. Ultrastructural studies on the brachiopod pedicle. *Lethaia* **11**, 331-339 (1978).
206. Stricker, S. & Reed, C. Development of the pedicle in the articulate brachiopod *Terebratalia transversa* (Brachiopoda, Terebratulida). *Zoomorphology* **105**, 253-264 (1985).
207. Orton. On ciliary mechanisms in brachiopods and some polychaetes, with a comparison of the ciliary mechanisms on the gills of molluscs, protochordata, brachiopods, and cryptocephalous polychaetes, and an account of the endostyle of *Crepidula* and its allies. *J. Mar. Biol. Ass. UK* **10**, 283-311 (1914).
208. Venkatesh, B. *et al.* Elephant shark genome provides unique insights into gnathostome evolution. *Nature* **505**, 174-179 (2014).
209. Sanggaard, K. W. *et al.* Spider genomes provide insight into composition and evolution of venom and silk. *Nat. Commun.* **5** (2014).
210. Ihaka, R. & Gentleman, R. R: a language for data analysis and graphics. *J. Comput. Graph. Stat.* **5**, 299-314 (1996).
211. Gentleman, R. C. *et al.* Bioconductor: open software development for computational biology and bioinformatics. *Genome Biol.* **5**, R80 (2004).
212. pheatmap: Pretty Heatmaps v. 077 (2014).
213. Kawasaki, K., Suzuki, T. & Weiss, K. M. Genetic basis for the evolution of vertebrate mineralized tissue. *Proc. Natl. Acad. Sci. USA* **101**, 11356-11361 (2004).
214. Livingston, B. T. *et al.* A genome-wide analysis of biomineralization-related proteins in the sea urchin *Strongylocentrotus purpuratus*. *Dev. Biol.* **300**, 335-348 (2006).
215. Hattan, S. J., Laue, T. M. & Chasteen, N. D. Purification and characterization of a novel calcium-binding protein from the extrapallial fluid of the mollusc, *Mytilus edulis*. *J. Biol. Chem.* **276**, 4461-4468 (2001).
216. Liu, H. L. *et al.* Identification and characterization of a biomineralization related gene PFMG1 highly expressed in the mantle of *Pinctada fucata*. *Biochemistry* **46**, 844-851 (2007).
217. Huan, P., Liu, G., Wang, H. & Liu, B. Identification of a tyrosinase gene potentially involved in early larval shell biogenesis of the Pacific oyster *Crassostrea gigas*. *Dev. Genes Evol.* **223**, 389-394 (2013).
218. Jackson, D., Worheide, G. & Degnan, B. Dynamic expression of ancient and novel molluscan shell genes during ecological transitions. *BMC Evol. Biol.* **7**, 160 (2007).
219. Sudo, S. *et al.* Structures of mollusc shell framework proteins. *Nature* **387**, 563-564 (1997).
220. Simmons, A. H., Michal, C. A. & Jelinski, L. W. Molecular orientation and two-component nature of the crystalline fraction of spider dragline silk. *Science* **271**, 84-87 (1996).
221. Mayumi, I., Hiroko, T., Yutaka, M. & Yoshinori, K. Difference of the organic component between the mineralized and the non-mineralized layers of *Lingula* shell. *Comp. Biochem. Physiol. A* **98**, 379-382 (1991).
222. De Robertis, E. M. & Sasai, Y. A common plan for dorsoventral patterning in Bilateria. *Nature* **380**, 37-40 (1996).
223. Saina, M., Genikhovich, G., Renfer, E. & Technau, U. BMPs and Chordin regulate patterning of the directive axis in a sea anemone. *Proc. Natl. Acad. Sci. USA* **106**, 18592-18597 (2009).
224. Hayward, D. C. *et al.* Localized expression of a *dpp/BMP2/4* ortholog in a coral embryo. *Proc. Natl. Acad. Sci. USA* **99**, 8106-8111 (2002).

225. Finnerty, J. R., Pang, K., Burton, P., Paulson, D. & Martindale, M. Q. Origins of bilateral symmetry: *Hox* and *dpp* expression in a sea anemone. *Science* **304**, 1335-1337 (2004).
226. Shimizu, K., Sarashina, I., Kagi, H. & Endo, K. Possible functions of *Dpp* in gastropod shell formation and shell coiling. *Dev. Genes Evol.* **221**, 59-68 (2011).
227. Zoccola, D. *et al.* Specific expression of BMP2/4 ortholog in biomineralizing tissues of corals and action on mouse BMP receptor. *Mar. Biotechnol.* **11**, 260-269 (2009).
228. Lu, T. M., Luo, Y. J. & Yu, J. K. BMP and Delta/Notch signaling control the development of amphioxus epidermal sensory neurons: insights into the evolution of the peripheral sensory system. *Development* **139**, 2020-2030 (2012).
229. Luo, Y. J. & Su, Y. H. Opposing Nodal and BMP signals regulate left–right asymmetry in the sea urchin larva. *PLoS Biol.* **10**, e1001402 (2012).
230. Rottinger, E., DuBuc, T. Q., Amiel, A. R. & Martindale, M. Q. Nodal signaling is required for mesodermal and ventral but not for dorsal fates in the indirect developing hemichordate, *Ptychodera flava*. *Biol. Open* (2015).
231. Genikhovich, G. *et al.* Axis patterning by BMPs: cnidarian network reveals evolutionary constraints. *Cell Rep.* (2015).
232. Hashimoto, N., Kurita, Y. & Wada, H. Developmental role of dpp in the gastropod shell plate and co-option of the dpp signaling pathway in the evolution of the operculum. *Dev. Biol.* **366**, 367-373 (2012).
233. Kin, K., Kakoi, S. & Wada, H. A novel role for *dpp* in the shaping of bivalve shells revealed in a conserved molluscan developmental program. *Dev. Biol.* **329**, 152-166 (2009).
234. Mann, K., Edsinger-Gonzales, E. & Mann, M. In-depth proteomic analysis of a mollusc shell: acid-soluble and acid-insoluble matrix of the limpet *Lottia gigantea*. *Proteome Sci.* **10**, 28 (2012).
235. Marie, B., Le Roy, N., Zanella-Cleon, I., Becchi, M. & Marin, F. Molecular evolution of mollusc shell proteins: insights from proteomic analysis of the edible mussel *Mytilus*. *J. Mol. Evol.* **72**, 531-546 (2011).
236. Marie, B. *et al.* Proteomic analysis of the organic matrix of the abalone *Haliotis asinina* calcified shell. *Proteome Sci.* **8**, 54 (2010).
237. Marin, F. & Luquet, G. Unusually acidic proteins in biomineralization. in *Handbook of Biomineralization* (ed E. Bäuerlein) Ch. 16, 273-290 (Wiley-VCH Verlag GmbH, 2008).
238. Ozbek, S., Balasubramanian, P. G., Chiquet-Ehrismann, R., Tucker, R. P. & Adams, J. C. The evolution of extracellular matrix. *Mol. Biol. Cell* **21**, 4300-4305 (2010).
239. Guerette, P. A., Ginzinger, D. G., Weber, B. H. & Gosline, J. M. Silk properties determined by gland-specific expression of a spider fibroin gene family. *Science* **272**, 112-115 (1996).
240. Takahashi, J. *et al.* A novel silk-like shell matrix gene is expressed in the mantle edge of the Pacific oyster prior to shell regeneration. *Gene* **499**, 130-134 (2012).
241. Huang, P. S. *et al.* High thermodynamic stability of parametrically designed helical bundles. *Science* **346**, 481-485 (2014).
242. Nair, A. K., Gautieri, A., Chang, S.-W. & Buehler, M. J. Molecular mechanics of mineralized collagen fibrils in bone. *Nat. Commun.* **4**, 1724 (2013).
243. Kawashima, T. *et al.* Domain shuffling and the evolution of vertebrates. *Genome Res.* **19**, 1393-1403 (2009).
244. Wu, Y. C., Rasmussen, M. D. & Kellis, M. Evolution at the subgene level: domain rearrangements in the *Drosophila* phylogeny. *Mol. Biol. Evol.* **29**, 689-705 (2012).
245. Wada, H., Okuyama, M., Satoh, N. & Zhang, S. Molecular evolution of fibrillar collagen in chordates, with implications for the evolution of vertebrate skeletons and chordate phylogeny. *Evol. Dev.* **8**, 370-377 (2006).
246. Drake, J. L. *et al.* Proteomic analysis of skeletal organic matrix from the stony coral *Stylophora pistillata*. *Proc. Natl. Acad. Sci. USA* **110**, 3788-3793 (2013).
247. Sansom, I. J., Smith, M. P., Armstrong, H. A. & Smith, M. M. Presence of the earliest vertebrate hard tissue in conodonts. *Science* **256**, 1308-1311 (1992).
248. Murdock, D. J. *et al.* The origin of conodonts and of vertebrate mineralized skeletons. *Nature* **502**, 546-549 (2013).

249. Smith, M. R. & Caron, J. B. Primitive soft-bodied cephalopods from the Cambrian. *Nature* **465**, 469-472 (2010).
250. Fedonkin, M. A. & Waggoner, B. M. The Late Precambrian fossil *Kimberella* is a mollusc-like bilaterian organism. *Nature* **388**, 868-871 (1997).
251. Caron, J. B., Scheltema, A., Schander, C. & Rudkin, D. A soft-bodied mollusc with radula from the Middle Cambrian Burgess Shale. *Nature* **442**, 159-163 (2006).
252. Tang, W. J., Fernandez, J. G., Sohn, J. J. & Amemiya, C. T. Chitin is endogenously produced in vertebrates. *Curr. Biol.* **25**, 897-900 (2015).
